# *Xanthomonas* adaptation to common bean is associated with horizontal transfers of genes encoding TAL effectors

**DOI:** 10.1186/s12864-017-4087-6

**Published:** 2017-08-30

**Authors:** Mylène Ruh, Martial Briand, Sophie Bonneau, Marie-Agnès Jacques, Nicolas W.G. Chen

**Affiliations:** IRHS, INRA, AGROCAMPUS OUEST, Université d’Angers, SFR4207 QUASAV, 42, rue Georges Morel, 49071 Beaucouzé, France

**Keywords:** *Xanthomonas*, Common bean, TAL effectors, Host adaptation, Horizontal gene transfer

## Abstract

**Background:**

Common bacterial blight is a devastating bacterial disease of common bean (*Phaseolus vulgaris*) caused by *Xanthomonas citri* pv. *fuscans* and *Xanthomonas phaseoli* pv. *phaseoli*. These phylogenetically distant strains are able to cause similar symptoms on common bean, suggesting that they have acquired common genetic determinants of adaptation to common bean. Transcription Activator-Like (TAL) effectors are bacterial type III effectors that are able to induce the expression of host genes to promote infection or resistance. Their capacity to bind to a specific host DNA sequence suggests that they are potential candidates for host adaption.

**Results:**

To study the diversity of *tal* genes from *Xanthomonas* strains responsible for common bacterial blight of bean, whole genome sequences of 17 strains representing the diversity of *X. citri* pv. *fuscans* and *X. phaseoli* pv. *phaseoli* were obtained by single molecule real time sequencing. Analysis of these genomes revealed the existence of four *tal* genes named *tal23A*, *tal20F*, *tal18G* and *tal18H*, respectively. While *tal20F* and *tal18G* were chromosomic, *tal23A* and *tal18H* were carried on plasmids and shared between phylogenetically distant strains, therefore suggesting recent horizontal transfers of these genes between *X. citri* pv. *fuscans* and *X. phaseoli* pv. *phaseoli* strains. Strikingly, *tal23A* was present in all strains studied, suggesting that it played an important role in adaptation to common bean. In silico predictions of TAL effectors targets in the common bean genome suggested that TAL effectors shared by *X. citri* pv. *fuscans* and *X. phaseoli* pv. *phaseoli* strains target the promoters of genes of similar functions. This could be a trace of convergent evolution among TAL effectors from different phylogenetic groups, and comforts the hypothesis that TAL effectors have been implied in the adaptation to common bean.

**Conclusions:**

Altogether, our results favour a model where plasmidic TAL effectors are able to contribute to host adaptation by being horizontally transferred between distant lineages.

**Electronic supplementary material:**

The online version of this article (10.1186/s12864-017-4087-6) contains supplementary material, which is available to authorized users.

## Background

Bacterial pathogens from the genus *Xanthomonas* harbour singular type III effectors called transcription activator-like (TAL) effectors that are able to mimic eukaryotic transcription factors [1–3]. After being injected into host cells via the type III secretion system, TAL effectors migrate to the nucleus where they act as transcription factors thanks to an activation domain localized in the C-terminal (C-ter) region [4, 5]. The central region of the protein is composed of a variable number of quasi-identical, 34 amino acids long repeats, except for the last repeat of the central region usually corresponding to a half-repeat of 18 amino acids. The pair of residues at positions 12 and 13 of each repeat is named repeat variable diresidue (RVD) and determines the binding specificity of a repeat to a single nucleotide. Thus, the RVD sequence of a TAL effector determines its binding specificity to a target DNA sequence called Effector Binding Element (EBE) [6, 7]. A TAL code linking each RVD to their nucleotide binding affinity has been deciphered, enabling in silico prediction of EBE for a given TAL effector in the host genome [6–11]. The EBE is usually located in the promoter region of susceptibility genes, enabling TAL effectors to promote bacterial infection [12–15]. In some cases, the EBE is located upstream of resistance genes termed *executor*, leading to the defence reaction of the host [16, 17].

Because the function of a TAL effector requires its specific binding to DNA, each EBE is a potential source of resistance that can be exploited for developing innovative disease resistance strategies [18, 19]. For instance, any modification of an EBE can lead to loss of susceptibility because the TAL effector is not able to bind to the EBE anymore. Such strategy has been successfully used to engineer resistance of rice to *X. oryzae* pv. *oryzae* by genome editing [20, 21]. Another strategy consists in trapping TAL effectors by placing EBE of conserved TAL effectors in the promoter regions of resistance genes [22–25]. This has successfully been done in rice [26, 27]. These examples are proofs of concepts showing that TAL-based resistance engineering is possible. However, the durability of such resistances and their effectiveness against more diverse pathogens in field has still to be assessed. This requires broadening our knowledge of *tal* genes diversity in *Xanthomonas* populations. However, because of the repeated nature of their central domain, *tal* genes can be misassembled after whole genome sequencing based on Illumina, and/or 454 technologies [28]. Long-read sequencing strategies such as PacBio Single Molecule, Real-Time (SMRT) sequencing have been proved efficient for getting TAL effector sequences [29, 30].

Common bacterial blight of bean (CBB) is the most devastating bacterial disease on common bean. It is due to four different genetic lineages belonging to two distinct species within the genus *Xanthomonas* [31, 32]. The fuscous lineage (fuscans) and the non-fuscous lineages 2 (NF2) and 3 (NF3) all belong to *X. citri* pv. *fuscans* (formerly *X. fuscans* subsp. *fuscans* and *X. axonopodis* pv. *phaseoli* Rep-PCR group 9.6) while the non-fuscous lineage 1 (NF1) belongs to *X. phaseoli* pv. *phaseoli* (formerly *X. axonopodis* pv. *phaseoli* Rep-PCR group 9.4) [33–35]. Pathological convergence of strains responsible for CBB is apparently linked to large events of horizontal gene transfer (HGT) involving a hundred of genes [36]. High-quality genome sequencing of *X. citri* pv. *fuscans* strain CFBP4885 (synonym 4834-R) revealed the existence of two *tal* genes in this strain: *Xfutal1* and *Xfutal2*, both located on plasmids [37]. PCR assays suggested that both *Xfutal1* and *Xfutal*2 exist in the fuscans and NF1 lineages [36]. However, the diversity of TAL effectors in *X. citri* pv. *fuscans* and *X. phaseoli* pv. *phaseoli* has not yet been described.

In order to study the diversity of TAL effectors in *Xanthomonas* strains responsible for CBB, we have generated the whole genome sequence of 17 *X. citri* pv. *fuscans* and *X. phaseoli* pv. *phaseoli* strains by using PacBio SMRT sequencing. The sequence of *tal* genes was extracted and phylogenetic analyses including the *tal* gene sequences from other *Xanthomonas* strains were performed. The comparison of the phylogeny of *tal* genes with the corresponding phylogeny of strains allowed us to predict potential HGT events between the four *X. citri* pv. *fuscans* and *X. phaseoli* pv. *phaseoli* lineages, therefore suggesting a role of *tal* genes in host adaptation.

## Results

### Pathogenicity, genome quality and *tal* genes content

**Fig. 1:**
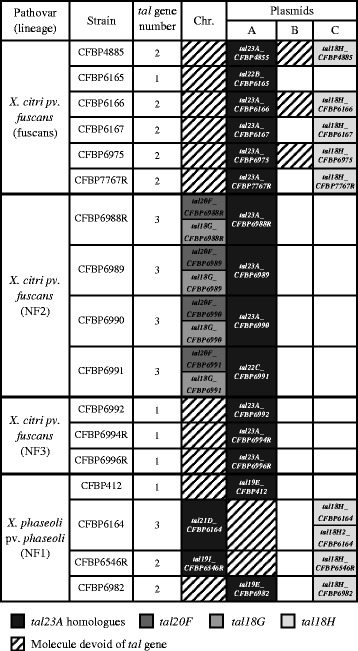
Localisation of *tal* genes within the genomes of 17 strains responsible for CBB. Presence of the corresponding molecule is represented by shades of grey depending on the presence of the different *tal* genes (see legend). Chr.: Chromosome

To estimate the diversity of *tal* genes present in strains responsible for CBB, the genome sequence of 17 strains representing the diversity of the four genetic lineages of *X. citri* pv. *fuscans* and *X. phaseoli* pv. *phaseoli* was obtained through PacBio SMRT sequencing (Table 1). All these strains were pathogenic on common bean, although with different degrees of aggressiveness (Table 1). The majority of strains (12/17) were highly aggressive on common bean while the other (5/17) were less aggressive. Overall, the average genome size and gene content (5.24 +/− 0.08 Mbp; 4596 +/− 160 genes) were quite classical for *Xanthomonas* strains [36–42]. Each genome sequence consisted of two to six contigs, with a maximum contig size corresponding to the expected chromosome size (5.1 Mbp) and additional circularized contigs corresponding to extrachromosomal plasmids. Plasmids A, B and C were named based on similarity with corresponding plasmids previously described in strain CFBP4885, also known as 4834-R [37] (Fig. 1).

**Table 1:** Genome features of the 17 bacterial strains responsible for CBB sequenced in this study

Pathovar (lineage)	Strain	Country (date of isolation)	Disease index^a^	Contig number	Genome size (bp)	Max. contig length (bp)	% GC	Gene number	GenBank Accession numbers
*X. citri* pv. *fuscans* (fuscans)	CFBP4885	France (1998)	3	6	5,173,315	5,012,288	64.66	4519	CP020992 to CP020997
	CFBP6165	Canada (1957)	3	3	5,118,412	5,054,301	64.80	4412	CP020998 to CP021000
	CFBP6166	South Africa (1963)	3	5	5,200,310	5,025,712	64.67	4566	CP021001 to CP021005
	CFBP6167	USA (1954)	2	5	5,388,310	5,176,780	64.54	4755	CP021018 to CP021022
	CFBP6975	France (1994)	3	5	5,244,382	5,092,018	64.64	4634	CP021006 to CP021010
	CFBP7767R	Cameroon (2009)	3	3	5,332,674	5,227,850	64.56	4893	CP021012 to CP021014
*X. citri* pv. *fuscans* (NF2)	CFBP6988R	La Réunion (2000)	3	2	5,199,272	5,122,265	64.62	4526	CP020979 to CP020980
	CFBP6989	La Réunion (2000)	3	2	5,179,726	5,111,201	64.62	4498	CP020981 to CP020982
	CFBP6990	La Réunion (2000)	3	2	5,193,607	5,123,413	64.64	4512	CP020983 to CP020984
	CFBP6991	La Réunion (2000)	3	3	5,309,897	5,107,678	64.59	4673	CP021015 to CP021017
*X. citri* pv. *fuscans* (NF3)	CFBP6992	La Réunion (2000)	1	2	5,299,787	5,232,548	64.57	4478	CP020985 to CP020986
	CFBP6994R	Tanzania (1990)	3	2	5,191,148	5,124,593	64.70	4399	CP020987 to CP020988
	CFBP6996R	La Réunion (2000)	2	2	5,145,832	5,078,988	64.73	4352	CP020989 to CP020990
*X. phaseoli* pv. *phaseoli* (NF1)	CFBP412	USA (NA)	2	3	5,186,811	5,071,497	64.89	4568	CP020964 to CP020966
	CFBP6164	Romania (1966)	2	4	5,399,156	5,254,225	64.63	4833	CP020967 to CP020970
	CFBP6546R	USA (NA)	3	4	5,258,370	5,081,709	64.78	4801	CP020971 to CP020974
	CFBP6982	La Réunion (2000)	3	4	5,291,156	5,100,676	64.75	4712	CP020975 to CP020978
			Average	3	5,241,892	5,117,514	64.67	4596	

^a^Disease symptoms were scored 11 days after bathing the first trifoliate leaf into bacterial suspensions at 1 × 10^7^ CFU/mL according to the following scale: 0 = no symptoms, 1 = 1 to 50 spots per leaf, 2 = 51 to 200 spots per leaf, necrosis and sagging and 3 = more than 200 spots per leaf, necrosis, sagging or leaf death. The average of pathogenicity scores were calculated from the values of three plants per strain

Following BLASTN searches in the 17 SMRT-sequenced genomes, a set of 26 complete *tal* genes and 14 putative *tal* pseudogenes were retrieved. Four pseudogenes corresponded to partial N-ter-encoding sequences, two corresponded to degenerated *tal* gene sequences bearing several frameshifts leading to premature stop codons, and eight corresponded to full-length *tal* genes containing a deletion of one nucleotide in either the N-ter- or the C-ter-encoding region (Additional file 1). Given the high rate of indels observed after SMRT sequencing [43], we sequenced the PCR products corresponding to these eight full-length *tal* pseudogenes with Sanger sequencing technology and all were validated as complete *tal* genes. This led to a total of 34 complete *tal* genes and six pseudogenes. Only complete *tal* genes were retained for further analyses (Additional file 2).

Strain CFBP4885 was previously sequenced using a combination of Illumina, 454 and Sanger technologies [37]. This high-quality genome sequencing revealed the existence of two *tal* genes (*Xfutal1* and *Xfutal2*) in this strain, therefore allowing comparison of *Xfutal1* and *Xfutal2* to the *tal* sequences obtained here by SMRT sequencing for the same strain. Unexpectedly, the distinct sequencing technologies differed in the predicted length of *tal* genes. The lengths of *Xfutal1* and *Xfutal2* in strain CFBP4885 have been previously estimated at 4935 bp and 3405 bp, respectively [37]. However, lengths of 4017 bp and 3507 bp were found for *tal* genes obtained after SMRT sequencing. These sequences differed only by their number of repeats, indicating that the observed differences in length were due to assembly errors in their repeat regions. To validate *Xfutal1* and *Xfutal2* sequence lengths, we designed PCR primers on conserved sequences corresponding to the N-ter- and C-ter-encoding regions of XfuTAL1 and XfuTAL2 proteins. PCR profiles were consistent with the lengths obtained after SMRT sequencing, and Sanger sequencing of the PCR products further confirmed that *Xfutal1* and *Xfutal2* sequences have been successfully sequenced and assembled in the SMRT-sequenced genomes (Additional file 3). These results corroborate previous analyses showing that SMRT sequencing led to accurate assembly of *tal* genes [29, 30].

**Fig. 2:**
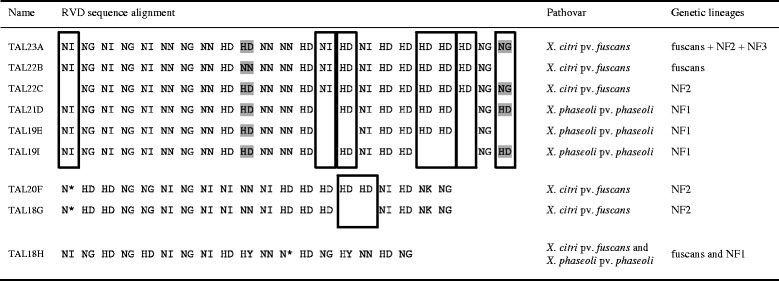
Alignment of the nine unique RVD sequences retrieved in TAL effectors from 17 strains responsible for CBB. TAL effectors that could be aligned are grouped together, indels are represented by black rectangles and putative substitutions are marked with grey colour

The length of the 34 complete *tal* genes found in our 17 genome sequences ranged between 3501 bp and 4017 bp, which corresponded to TAL effectors possessing from 18 to 23 repeats. The overall RVD composition was similar to that of previously sequenced *tal* genes from other *Xanthomonas* pathovars [4, 29] with more than 97% of RVD being HD, NN, NG, NI, N* or NK (Additional file 4). The only unusual RVD was HY, retrieved in nine TAL effectors and previously reported in only six other TAL effectors from *X. oryzae* pv. *oryzicola* and *Ralstonia solanacearum* [29, 44]. Nine unique RVD combinations ranging from 18 to 23 RVD were identified (Fig. 2). We named these nine unique RVD combinations using their number of repeats followed by a letter corresponding to each unique RVD combination, from A to I (Fig. 2). For example, for strain CFBP4885, corrected sequences of XfuTAL1 with 23 RVD and XfuTAL2 with 18 RVD corresponded to RVD combinations TAL23A and TAL18H, respectively. Following this nomenclature, *Xfutal1* and *Xfutal2* genes were renamed as *tal23A_CFBP488*5 and *tal18H*_*CFBP4885*, respectively (Figs. 1, 2).

### *tal* genes from *X. citri* pv. *fuscans* and *X. phaseoli* pv. *phaseoli* lineages originate from a single ancestor

**Fig. 3:**
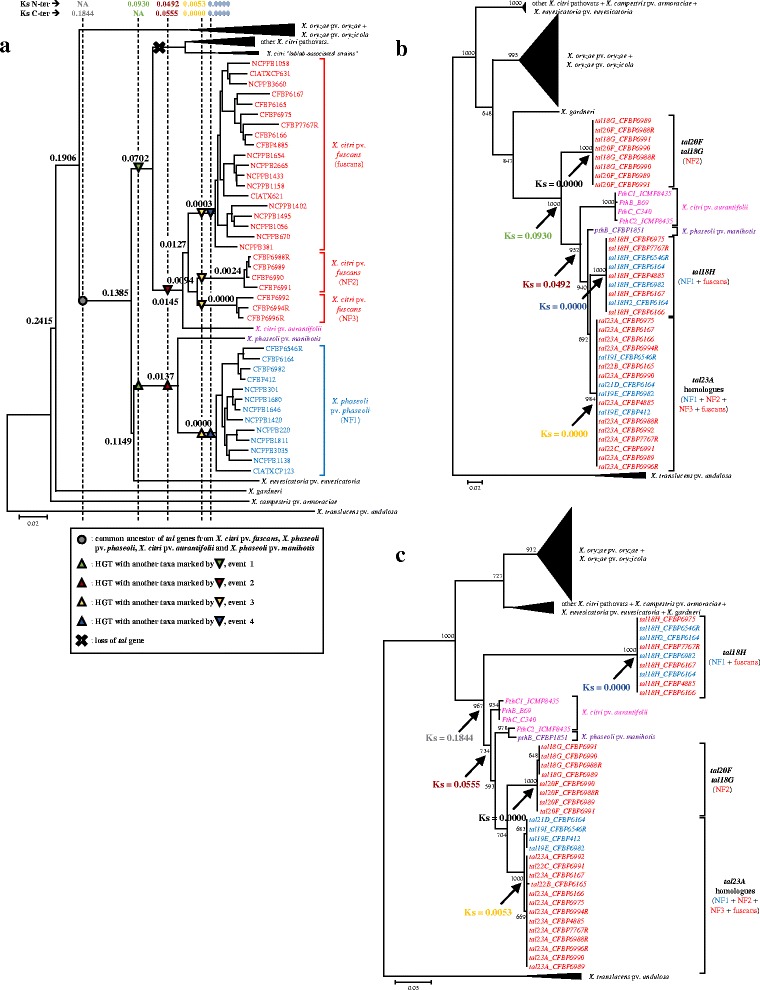
Phylogeny of *Xanthomonas* strains and *tal* genes. Strains or *tal* genes from the *X. citri* pv. *fuscans* genetic lineages fuscans, NF2 and NF3 are indicated in red, *X. phaseoli* pv. *phaseoli* NF1 lineage in blue, *X. citri* pv. *aurantifolii* in pink and *X. phaseoli* pv. *manihotis* in purple. Ks values corresponding to the divergence of *tal* genes are indicated in the different trees using the same colours. **a** Phylogenetic tree representing the evolution of *Xanthomonas* strains constructed using CVtree on the whole proteome of strains. Numbers at node correspond to mean Ks values calculated on seven housekeeping genes. Vertical dotted lines represent time of *tal* gene divergence estimated by Ks values written above. The most parsimonious course of events for the evolution of *tal* genes is highlighted according to the legend. NA: Not applicable. **b** ML tree constructed on the nucleotide sequences of N-ter-encoding region of *tal* genes. **c** ML tree constructed on the nucleotide sequences of C-ter-encoding region of *tal* genes. For (**b)** and (**c**), bootstrap values greater than 50% are shown for 1000 replicates and horizontal scale bars represent number of nucleotide substitutions per site

To study the evolution of *tal* genes from strains responsible for CBB, we compared the phylogeny of *tal* genes to the phylogeny of *X. phaseoli* pv. *phaseoli* and *X. citri* pv. *fuscans* strains. For this, we first reconstructed the phylogeny of organisms using the whole predicted proteomes of the 17 *X. phaseoli* pv. *phaseoli* and *X. citri* pv. *fuscans* strains from our study plus 31 strains representing 10 *Xanthomonas* pathovars from which complete TAL effectors have previously been found, and 26 additional strains responsible for CBB sequenced previously (Additional file 5). The CVTree topology (Fig. 3a) was congruent with previously published *Xanthomonas* phylogenetic trees [33, 35, 45]. As previously shown, strains responsible for CBB split into four distinct genetic lineages [31, 46]. Three lineages, fuscans, NF2 and NF3 belonged to the *X. citri* species and one lineage, NF1, belonged to the *X. phaseoli* species (Fig. 3a). These groupings were confirmed by an average nucleotide identity above 99% within each genetic lineage, and under 95% between *X. phaseoli* pv. *phaseoli* and the three other lineages (Additional file 6).

In evolutionary studies of *tal* genes, the central region is usually discarded because it is composed of a variable number of highly-conserved repeats that are able to recombine with each other, which can obscure alignments and phylogeny [29, 30]. Therefore, we reconstructed two phylogenies of *tal* genes using DNA sequences corresponding either to the N-ter- or to the C-ter-encoding region of complete TAL effectors (Figs. 3b, 3c). Both trees had a topology different from the organism CVTree, suggesting that recombination and/or horizontal transfers of *tal* genes occurred during the diversification of xanthomonads. In particular, *tal* genes from *X. citri* pv. *fuscans* and *X. phaseoli* pv. *phaseoli* clustered into a monophyletic group, together with *tal* genes from *X. citri* pv. *aurantifolii* and *X. phaseoli* pv. *manihotis*. For both trees, *tal* genes from *X. citri* pv. *fuscans* and *X. phaseoli* pv. *phaseoli*, were divided in three clades (Fig. 3b, c).

The CDS corresponding to RVD combinations TAL23A, TAL22B, TAL22C, TAL21D, TAL19E and TAL19I appeared to be derived from a recent common ancestor (Fig. 3b, c). Interestingly, every strain responsible for CBB possessed a single CDS corresponding to one of these six RVD combinations. These CDS were located on plasmid A for a majority of strains (15/17) except for strains CFBP6164 and CFBP6546R where it was located on the chromosome (Fig. 1). Also, all these CDS were surrounded by a conserved 17 kbp region whatever their location (see detailed explanation in the fifth section below). Together, these observations indicate that these CDS corresponded to allelic versions of a single gene, thus they were all considered as *tal23A* homologues. These *tal23A* homologues diverged by indels of complete internal repeats and/or by substitutions or recombinations affecting their RVD content (Fig. 2). Two additional genes, *tal20F* and *tal18G*, were only found in the four strains belonging to the NF2 lineage and had a chromosomic location (Fig. 1). These two *tal* genes were very similar to each other (Fig. 3b and c) and diverged by an indel involving two contiguous RVD (Fig. 2). Both *tal* genes were retrieved in every NF2 strain, indicating that they corresponded to paralogues resulting from a duplication predating the diversification of the NF2 lineage. Finally, *tal18H* was retrieved in most strains belonging to the fuscans (5/6) or NF1 (3/4) lineages, and was always located on plasmid C (Fig. 1). Notably, the three strains from the NF3 lineage did not present any additional *tal* gene other than *tal23A* (Fig. 1).

Partial *tal* sequences retrieved in previously sequenced genomes from the NF1 and fuscans lineages [36] confirmed that *tal* genes found in both lineages were all related to *tal23A* or *tal18H* genes (Additional file 7). Also, strains responsible for CBB isolated on lablab bean (*Dolichos lablab*), a legume plant phylogenetically close to common bean, have previously been described [36]. These strains clustered with *X. citri* pathovars other than *X. citri* pv. *fuscans* and *X. citri* pv. *aurantifolii* (Fig. 3a). Partial *tal* sequences were retrieved in the genomes from these lablab-associated strains. Strikingly, these *tal* genes did not cluster with any of the three aforementioned clades, further suggesting that TAL effectors from *X. phaseoli* pv. *phaseoli* and *X. citri* pv. *fuscans* were specific for strains having common bean as natural host.

### Recombination occurred between *tal* genes from *X. citri* pv. *fuscans* and *X. phaseoli* pv. *phaseoli*

We observed discrepancies between the trees generated with the N-ter- or C-ter-encoding sequences. Indeed, in the tree generated using the C-ter-encoding sequence, *tal18G* and *tal20F* formed a clade external to the other *tal* genes from strains responsible for CBB, while in the tree generated using the N-ter-encoding sequence, *tal18H* clustered outside from the others (Fig. 3b, c). Tree topologies were significantly different (*P* < 0.05) based on the Kishino-Hasegawa-Templeton test [47]. This suggested that a recombination event occurred between the N-ter and C-ter encoding regions of these *tal* genes. To confirm this observation, we estimated recombination using seven nonparametric detection programs from the RDP4 package [48]. Two recombination events were retrieved on an alignment of the concatenated N-ter- and C-ter-encoding regions of *tal* genes from *X. citri* pv. *fuscans*, *X. citri* pv. *aurantifolii*, *X. phaseoli* pv. *phaseoli* and *X. phaseoli* pv. *manihotis* strains, but none were retrieved on the separated N-ter- or C-ter-encoding regions, suggesting that recombination breakpoints were located within the central repeat region of *tal* genes (data not shown). The first event was detected in *tal18H*, predicted as recombinants between the common ancestor of *tal18G* and *tal20F*, and *pthB* from the *X. phaseoli* pv. *manihotis* strain. The second event was detected within *pthB*, predicted as a recombinant between *PthC2* and other *tal* genes from *X. citri* pv. *aurantifolii*. Both recombination events were in accordance with the differences observed between the tree topologies (Fig. 3b, c).

### *tal* genes have undergone multiple horizontal transfers between *X. citri* pv. *fuscans* and *X. phaseoli* pv. *phaseoli* lineages

To estimate the time of divergence between *tal* genes, we compared the values of pairwise nucleotide synonymous substitution rates at silent sites (Ks) from the N-ter or C-ter-encoding regions of *tal* genes to Ks values from seven housekeeping genes (*atpD*, *dnaK*, *efp*, *glnA1*, *gyrB*, *rpoD* and *suxA*) [46] after removal of recombinant regions. In absence of codon usage bias, and for genes under constant evolutionary rates, Ks is an estimation of neutral evolution because it does not take into account the nonsynonymous sites that can be under selection pressure [49, 50]. Here, codon usage was constant among the different studied taxa, and no significant divergence from neutrality was retrieved (*P* > 0.05) after Tajima’s or Fu and Li’s relative rate tests (data not shown). Therefore, Ks could be used as an approximation of the time of divergence between genes or taxa [51], with higher Ks meaning longer time of evolution between two sequences.

The mean Ks for *tal* genes from *X. citri* pv. *fuscans*, *X. phaseoli* pv. *phaseoli*, *X. citri* pv. *aurantifolii* and *X. phaseoli* pv. *manihotis* strains were 0.0930 and 0.1844 for the N-ter- and C-ter-encoding regions, respectively (Fig. 3b, c). Thus, Ks value for the C-ter-encoding region was approximately twice as high as the Ks value for the N-ter-encoding region, indicating that the C-ter-encoding region has accumulated more synonymous substitutions at silent sites than the N-ter-encoding region did. This suggested that the C-ter-encoding region had a more ancient origin than the N-ter-encoding region in these strains. The Ks value for the C-ter-encoding region (0.1844) was in the same range as the Ks value calculated for housekeeping genes corresponding to the split between the *X. oryzae* species and the *X. axonopodis* species complex (0.1906), suggesting that the C-ter-encoding region has been transmitted vertically from the ancestor of *X. axonopodis* to the present days (Fig. 3a). In contrast, Ks value for the N-ter-encoding region (0.0930) was lower than the Ks value for housekeeping genes corresponding to the split between *X. euvesicatoria* and *X. phaseoli* species (Ks = 0.1149) and higher than the Ks value corresponding to the emergence of the *X. citri* species (Ks = 0.0702). This suggested that the N-ter-encoding region of *tal* genes from *X. citri* pv. *fuscans*, *X. citri* pv. *aurantifolii*, *X. phaseoli* pv. *phaseoli* and *X. phaseoli* pv. *manihotis* diverged after the split between *X. euvesicatoria* and *X. phaseoli* species and before the emergence of the *X. citri* species. This can be explained by a HGT between the ancestors of the *X. phaseoli* and *X. citri* species (event 1 on Fig. 3). Then, absence of orthologous *tal* genes in other *X. citri* pathovars would be due to a *tal* gene loss (Fig. 3a).

For both N-ter- and C-ter-encoding regions, *tal* genes from *X. citri* pv. *aurantifolii*, *X. citri* pv. *fuscans*, *X. phaseoli* pv. *manihotis* and *X. phaseoli* pv. *phaseoli* strains were grouped in a clade having *tal18G* and *tal20F* or *tal18H* as direct outgroups, respectively (Fig. 3b and c). In absence of HGT, the time of divergence for this clade should reflect the time of divergence between the *X. citri* and *X. phaseoli* species (Ks = 0.1385). However, the N-ter- and C-ter-encoding regions diverged at Ks values of 0.0492 and 0.0555, respectively (Fig. 3b and c). These Ks values were similar to each other, and lower than the Ks at the split between *X. citri* pathovars *aurantifolii* plus *fuscans* and the other *X. citri* pathovars (0.0702), but higher than Ks at the split between *X. citri* pv. *aurantifolii* and *X. citri* pv. *fuscans* (0.0145) and at the split between *X. phaseoli* pv. *manihotis* and *X. phaseoli* pv. *phaseoli* (0.0137; Fig. 3a). This suggested that a HGT of *tal* genes occurred between the ancestor of *X. citri* pv. *aurantifolii* and *X. citri* pv. *fuscans*, and the ancestor of *X. phaseoli* pv. *manihotis* and *X. phaseoli* pv. *phaseoli* (event 2 on Fig. 3a).

Presence of *tal23A* homologues in the phylogenetically distant *X. citri* pv. *fuscans* and *X. phaseoli* pv. *phaseoli* strains suggested that these *tal* genes were horizontally transferred between the ancestor of the NF1 lineage and the ancestor of one of the three *X. citri* pv. *fuscans* lineages (event 3 on Fig. 3a). Ks values for *tal23A* homologues between *X. citri* pv. *fuscans* and *X. phaseoli* pv. *phaseoli* lineages were zero for the N-ter-encoding region and 0.0053 for the C-ter-encoding region, indicating that *tal23A homologues* accumulated very few synonymous substitutions between *X. citri* pv. *fuscans* and *X. phaseoli* pv. *phaseoli* lineages, and indicating that this HGT was very recent. These low Ks values were similar to the values found within each lineage for housekeeping genes (NF1: 0.0000; NF2: 0.0024; NF3: 0.0000; fuscans: 0.0003), further confirming that this HGT occurred in the same time frame as the divergence of strains within each lineage. Moreover, most *tal23A* homologues were located on plasmid A. This plasmid was present in all *X. citri* pv. *fuscans* and *X. phaseoli* pv. *phaseoli* strains but not in *X. citri* pv. *aurantifolii* and *X. phaseoli* pv. *manihotis* strains [52, 53] (Fig. 1). Plasmid A was highly conserved with, on average, more than 97% nucleotide identity on more than 36% of the total plasmid length between the strains from the three *X. citri* pv. *fuscans* lineages and the strains from the NF1 lineage (Additional file 8, Additional file 9). This suggested that the event responsible for the horizontal transfer of *tal23A* homologues corresponded in fact to the horizontal transfer of a large portion of plasmid A. The largest conserved portion of plasmid A between *X. citri* pv. *fuscans* and *X. phaseoli* pv. *phaseoli* strains corresponded to 76% of plasmid A from strain CFBP6546R (NF1 lineage) and 77% of plasmid A from strain CFBP6988R (NF2 lineage) (Additional file 9). Thus, the most probable event would correspond to a HGT between the NF2 and NF1 lineages. The region between strains CFBP6988R and CFBP6546R corresponded to 39 kbp comprising 38 predicted protein-encoding genes (Additional file 10a, Additional file 11). These predicted proteins included 11 hypothetical proteins and 27 proteins with predicted functions including seven proteins involved in the type IV conjugal transfer system, three transposases, and two DNA topoisomerases (Additional file 11). In strain CFBP6546R, the *tal23A* homologue was located on the chromosome. Therefore, it was not retrieved in the 39 kbp region shared by both strains. In strain CFBP6988R, this *tal23A* homologue was located contiguous to the 39kbp region for a total of ~44 kbp (Additional file 10a). This suggests that a region of ~44 kbp from plasmid A was horizontally transferred between the NF2 and NF1 lineages, followed by the integration of *tal23A* homologues in the chromosome of some strains belonging to the NF1 lineage (this event is explained with more details in the next section).

Finally, *tal18H* was common to most strains from the NF1 and fuscans lineages but absent in the other lineages, suggesting that *tal18H* has been horizontally transferred between the ancestors of NF1 and fuscans strains (event 4 on Fig. 3a). In accordance with this, Ks values between NF1 and fuscans lineages were zero for both N-ter and C-ter-encoding regions, indicating that *tal18H* did not accumulate any nonsynonymous substitutions between the NF1 and fuscans lineages. Moreover, *tal18H* was located on plasmid C, and its presence was directly correlated with the presence of plasmid C (Fig. 1). Plasmid C was highly conserved with, on average, more than 99% nucleotide identity on more than 59% of the total plasmid length between NF1 and fuscans strains (Additional file 8, Additional file 12), suggesting that *tal18H* has been horizontally transferred along with a large portion of plasmid C. The largest conserved portion between *X. citri* pv. *fuscans* (fuscans) and *X. phaseoli* pv. *phaseoli* (NF1) strains corresponded to 68% of plasmid C from strain CFBP6982 (NF1 lineage) and 76% of plasmid C from strain CFBP6166 (fuscans lineage) (Additional file 12). The horizontally transferred region corresponded to ~30 kbp that were split in different smaller regions and/or duplicated depending on the strain, suggesting that recombination and/or local duplications occurred after the HGT event (Additional file 10b, Additional file 13). In addition to the *tal18H* gene, these regions comprised 26 predicted protein-encoding genes including eight hypothetical proteins and 18 proteins with predicted functions including the type III effector XopC1, two proteins involved in the type IV conjugal transfer system, three ribonucleases and two type II-like restriction endonucleases (Additional file 13). Strains CFBP412 and CFBP6165 were the only strains from the NF1 and fuscans lineages not possessing plasmid C, suggesting that they recently lost this plasmid, together with the *tal18H* gene (Fig. 1).

### Recent *tal* gene duplications and movements are associated with the presence of insertion sequences (IS) and transposons

Presence of IS and/or Tn3-like transposons has been proved as a hallmark of *tal* gene movements and duplication [54]. Here, all *tal* genes from *X. citri* pv. *fuscans* and *X. phaseoli* pv. *phaseoli* were associated with IS3 and/or Tn3-like transposons. To estimate if these IS3 and/or Tn3-like transposons were responsible for *tal* gene movements or duplications, we analysed the genomic environment of relocated or duplicated *tal* genes.

As described above, our analyses indicated that *tal23A* homologues have been transmitted by a HGT between *X. citri* pv. *fuscans* and *X. phaseoli* pv. *phaseoli* strains involving plasmid A. The majority of *tal23A* homologues (15/17) was located on plasmid A except for two *X. phaseoli* pv. *phaseoli* strains, CFBP6164 and CFBP6546R, where *tal21D_CFBP6164* and *tal19I_CFBP6546R* had a chromosomic location (Fig. 1). Strains CFBP6164 and CFBP6546R were closer to each other than any other strains (Fig. 3a). This suggested that the *tal* gene moved from the plasmid to the chromosome in the common ancestor of strains CFBP6164 and CFBP6546R. Analysis of the genomic environment indicated that this movement was associated with the relocation of a 17 kbp region surrounding this *tal* gene (Additional file 14a). Presence of intact IS3 sequences framing the receiving region of the chromosome of CFBP6164 and CFBP6546R strains, and presence of an IS3 remnant at the donor site in plasmid A in these strains suggested that recombination between IS3 sequences was involved in this movement, although we could not define the breakpoints precisely (Additional file 14a).

In each NF2 strain, *tal* genes *tal20F* and *tal18G* had nearly-identical sequences*.* These *tal* genes were not retrieved outside from the NF2 lineage, and the Ks value between these two copies was zero for both N-ter- and C-ter-encoding regions, indicating that these two genes resulted from a duplication in the direct ancestor of the NF2 lineage (Figs. 1 and 3). These *tal* genes were oriented back to back on the chromosome, and separated by around 720 kb. Both *tal* genes were surrounded by two Tn3-like transposons that were intact except for the region downstream *tal18G* (Additional file 14b). This suggested that this duplication involved a recombination between Tn3-like transposons, and indicated that *tal18G* was a copy of *tal20F*.

In plasmid C, *tal18H* was present as a single copy except for the CFBP6164 strain where two copies were found on plasmid C (*tal18H_CFBP6164* and *tal18H2_CFBP6164*). This duplication corresponded to the tandem duplication of a seven kbp region including the *tal* gene and flanked by two IS3 sequences, suggesting that this duplication was the consequence of an unequal crossing-over between these two IS3 (Additional file 14c). Interestingly, the N-ter-encoding region of *tal18H2_CFBP6164* comprised the insertion of a cytosine 88 bp after the start codon. This led to a frameshift resulting in the relocation of the start codon 27 aminoacids upstream of the classical N-ter-encoding region and modifying the first 30 aminoacids of the TAL18H2_CFBP6164 effector.

### In silico predictions suggest that *tal18H and tal23A* homologues were involved in *X. citri* pv. *fuscans* and *X. phaseoli* pv. *phaseoli* adaptation to common bean

**Fig. 4:**
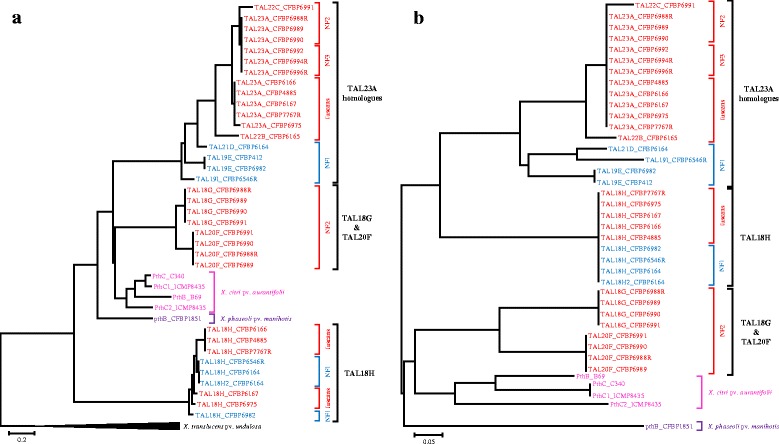
Distance trees of repeat regions or RVD sequences of TAL effectors. TAL effectors from the *X. citri* pv. *fuscans* genetic lineages fuscans, NF2 and NF3 are indicated in red, *X. phaseoli* pv. *phaseoli* NF1 lineage in blue, *X. citri* pv. *aurantifolii* in pink and *X. phaseoli* pv. *manihotis* in purple. **a** Phylogenetic classification of the repeat regions of TAL effectors constructed with the DisTAL program (Pérez-Quintero et al., 2015). Analysis was performed on amino acid sequences of the repeat region (excluding RVD) of TAL effectors from *X. citri* pv. *fuscans, X. citri* pv. *aurantifolii, X. phaseoli* pv. *phaseoli* and *X. phaseoli* pv. *manihotis*, using eight TAL effectors from *X. translucens* pv. *undulosa* XT4699 as outgroups. **b** Neighbour Joining tree representing the distance between RVD sequences, constructed using the FuncTAL program. Analysis was performed on the RVD sequences of TAL effectors from *X. citri* pv. *fuscans, X. citri* pv. *aurantifolii, X. phaseoli* pv. *phaseoli* and *X. phaseoli* pv. *manihotis*

The repeat region of TAL effectors is important because it is involved in the binding specificity of TAL effectors to the promoters of targeted host plant genes [6, 7]. Previous studies suggested that recombination, duplication and deletion events within repeats were involved in *tal* gene evolution [54–56]. To study the evolution of the repeat regions of *tal* genes from *X. citri* pv. *fuscans* and *X. phaseoli* pv. *phaseoli*, we used the DisTAL program that is dedicated to the analysis of the evolution of TAL repeats excluding the RVD [57]. TAL23A homologues were distributed according to their genetic lineages (Fig. 4a). An important diversification was observed for the NF1 lineage that comprised genes encoding RVD combinations TAL19E, TAL19I and TAL21D (Figs. 2, 4a). In fuscans*,* NF2 and NF3 lineages, repeats were less diversified and TAL23A RVD combination was retrieved in 11 strains out of 13. An interesting point is that genes encoding TAL23A RVD combination presented a lineage-dependant diversification of their repeats outside of the RVD, while keeping the same RVD combination. Indeed, repeats encoding TAL23A in the NF2 and NF3 lineages were divergent from those found in the fuscans lineage (Fig. 4a). Also, within genes encoding TAL23A RVD combination in the fuscans lineage, *tal23A_CFBP6975* presented a repeat region with a slight divergence from its orthologues in other strains from the fuscans lineage. Similarly, diversification occurred in the repeats of *tal18H*, leading to two different repeat profiles in the NF1 lineage and three in the fuscans lineage (Figs. 2, 4a). Thus, for TAL23A and TAL18H RVD combinations, RVD were retained despite repeat diversification, suggesting that these RVD combinations were functionally important for CBB causal agents.

We performed a comparative analysis of the 772 individual repeats from the 39 TAL effectors retrieved in *X. citri* pv. *fuscans*, *X. phaseoli* pv. *phaseoli*, *X. citri* pv. *aurantifolii* and *X. phaseoli* pv. *manihotis* stains after removal of the RVD. These repeats were distributed in 48 unique repeat sequences (Additional file 15). All repeats comprised 34 amino-acids, or 18 amino-acids for the last half-repeat, and no repeat of unusual length was retrieved [58]. One predominant repeat was found 259 times (in red in Additional file 15, Additional file 16), while 14 repeats were retrieved only once. Interestingly, the predominant repeat was conserved in all TAL effectors whatever the associated RVD. Also, this repeat differed by at least one amino acid from any other repeat found in other pathovars described so far (data not shown), further highlighting that *tal* genes from *X. citri* pv. *fuscans*, *X. phaseoli* pv. *phaseoli*, *X. citri* pv. *aurantifolii* and *X. phaseoli* pv. *manihotis* strains share a common evolutionary history different from the evolutionary history of *tal* genes found in other *Xanthomonas* strains. A second repeat found 33 times was shared by all TAL effectors whatever the associated RVD, except for *X. phaseoli* pv. *manihotis* (in green in Additional file 15, Additional file 16). A third repeat found 31 times was shared among TAL23A homologues, TAL20F, TAL18G, and PthC from *X. citri* pv. *aurantifolii* strain (in purple in Additional file 15, Additional file 16). Two additional repeats were specific for TAL effectors from *X. citri* pv. *aurantifolii* and *X. phaseoli* pv. *manihotis* strains (in blue and yellow in Additional file 15, Additional file 16). This is in agreement with the DisTAL tree general topology (Fig. 4a) and suggested that TAL effectors of *X. citri* pv. *aurantifolii* and *X. phaseoli* pv. *manihotis* strains had a specific evolutionary history compared to *X. citri* pv. *fuscans* and *X. phaseoli* pv. *phaseoli* strains.

As mentioned before, RVD diversification of TAL effectors from CBB causal agents led to nine different RVD combinations (Fig. 2). To analyse the functional relationships between these TAL effectors, we used the FuncTAL program that compares DNA binding specificities using RVD sequences [57]. Despite the diversification of RVD sequences observed among TAL23A homologues, or between TAL20F and TAL18G, the FuncTAL tree showed that TAL effectors coming from a same clade clustered together (Fig. 4b). Interestingly, TAL23A homologues were closer to TAL18H than to TAL20F and TAL18G, while their repeats were more distant from TAL18H according to the DisTAL tree. This could be a trace of functional convergence between TAL23A homologues and TAL18H.

To search for putative common targets of TAL effectors within and between the three phylogenetic groups of *tal* genes, we used the TALVEZ EBE prediction tool on the promoterome (3 kb-promoter regions) of the common bean genome sequence [11, 59]. For each single RVD combination found in TAL effectors from *X. phaseoli* pv. *phaseoli* and *X. citri* pv. *fuscans* strains, the top 10 predicted targets according to the scores calculated by TALVEZ did not correspond to any other previously described target of TAL effectors from other *Xanthomonas* pathovars [12, 18, 60–67] (Additional file 17). However, several top 10 predicted targets were shared by TAL effectors coming from a same phylogenetic group. Indeed, eight predicted targets were shared by at least two of the six TAL23A homologues, including two targets predicted for four RVD combinations (Additional file 17). This was the case for a peptidase (Phvul.006G032900) predicted for TAL23A, TAL22B, TAL19E and TAL19I, and a 40S ribosomal subunit (Phvul.002G052200) predicted for TAL23A, TAL22C, TAL21D and TAL19I. In addition, two different genes encoding nucleolar ribonucleoprotein subunits (Phvul.008G022975 and Phvul.003G201700) were predicted as targets for TAL23A, TAL22B and TAL21D (Additional file 17). Also, four out of the first 10 predicted targets were shared by TAL20F and TAL18G. This included a protein of unknown function (Phvul.005G097300), a carboxyl methyltransferase (Phvul.006G048600), a nucleic acid deaminase (Phvul.003G292800) and an oligopeptide transporter-related protein (Phvul.010G009400). Interestingly, two genes encoding UDP-glycosyl transferases (Phvul.003G097300 and Phvul.009G148800) were predicted as targets for TAL23A, TAL22C and TAL21D as well as for TAL18H, further suggesting that a functional convergence exist between TAL23A homologues and TAL18H (Additional file 17).

## Discussion

To study the diversity of TAL effectors from *Xanthomonas* strains responsible for CBB, we generated whole genome sequences for 17 strains representing the diversity of the four *X. citri* pv. *fuscans* and *X. phaseoli* pv. *phaseoli* genetic lineages. PacBio SMRT sequencing assemblies allowed us to retrieve 40 sequences of complete *tal* genes and pseudogenes. Previously, only two *tal* genes, *Xfutal1* and *Xfutal2* from CFBP4885 strain were reported for CBB agents [37]. Here, we show that *Xfutal1* and *Xfutal2* repeat regions contained assembly errors despite having been sequenced using a combination of Sanger, Illumina and 454 sequencing strategies. We thus confirmed that SMRT sequencing is a good methodology to obtain the sequence of TAL effector repeats [29, 30, 68, 69], which is essential for good prediction of their potential targets in host genomes [6, 7, 11]. However, corrections had to be made for deletions observed in the N-ter- and/or C-ter-encoding regions. These deletions were observed only in G- or C-rich stretches, which could correspond to error hotspots for SMRT sequencing.

Recombination and HGT are important driving forces of ecological adaptation and diversification in bacteria such as xanthomonads [45, 70–72]. Here, phylogenetic analyses revealed that the overall topology of *tal* genes differed from the topology of strains, indicating that the evolution of *tal* genes has been impacted by different events of recombination and/or HGT (Fig. 3). It was particularly interesting to observe that whether for the repeats, the N-ter-, or the C-ter-encoding regions, *tal* genes from *X. citri* pv. *fuscans*, *X. citri* pv. *aurantifolii, X. phaseoli* pv. *phaseoli* and *X. phaseoli* pv. *manihotis* strains clustered in a single clade (Figs. 3b, c, 4a). This strongly supports a scenario where *tal* genes from these four pathovars share a common ancestor different from *tal* genes found in other *Xanthomonas* species. Ks values for the N-ter- and C-ter-encoding regions suggested that two HGT involving *tal* genes occurred between the ancestors of *X. citri* and *X. phaseoli* species, and between the ancestors of *X. citri* pv. *aurantifolii* and *X. citri* pv. *fuscans* strains and the ancestor of *X. phaseoli* pv. *manihotis* and *X. phaseoli* pv. *phaseoli* strains (events 1 and 2 on Fig. 3a). Absence of orthologues in *X. citri* pathovars other than *X. citri* pv. *aurantifolii* and *X. citri* pv. *fuscans* certainly should be a result of a specific gene loss in these other *X. citri* pathovars. In particular, *X. citri* strains that were isolated on lablab and able to cause CBB on common bean [36, 73] did not possess any *tal* gene orthologous to the *tal* genes found in strains isolated on common bean. This further strengthens that the *tal* genes described here are specific to *Xanthomonas* strains having common bean as a natural host. Also, it showed that these lablab-associated strains were able to cause CBB symptoms on common bean without any contribution of the TAL effectors found in the genuine common bean pathogens, suggesting that these TAL effectors were not fully needed for pathogenicity and would rather contribute to aggressiveness. It would be interesting to obtain the RVD combination of TAL effectors from lablab-associated strains to analyse the contribution of these TAL effectors to the aggressiveness of *Xanthomonas* strains on common bean.

In strains responsible for CBB, *tal* gene diversification led to three distinct clades of *tal* genes. Subsequent to this diversification, we observed *tal* gene duplications and movements associated with IS and Tn3-like transposons, in accordance with previous observations in *X. citri* subsp. *citri* and other xanthomonads [54]. IS and transposons appear to have facilitated recombination, thus contributing to *tal* gene duplications and movements in strains responsible for CBB. It is tempting to speculate that the transfer of *tal23A* homologues that was observed from the plasmid A to the chromosome in strains CFBP6164 and CFBP6546 (Additional file 14) would allow a stable maintenance of these *tal* genes in the genome, and thus would underline the primary importance of *tal23A* homologues in adaptation of *Xanthomonas* to common bean.

Combination of phylogeny and Ks analyses unveiled events of recombination and HGT that shaped the evolution of *tal* genes in strains responsible for CBB. First, these analyses showed that the different *X. citri* pv. *fuscans* and *X. phaseoli* pv. *phaseoli* lineages bear different combinations of *tal* genes, suggesting that these lineages evolved in different ways for manipulating the common bean transcriptome. Strains from *X. citri* pv. *fuscans* NF3 lineage contained *tal23A* homologues only. Strains from *X. citri* pv. *fuscans* NF2 lineage also contained *tal23A* homologues, and were the only strains carrying *tal20F* and *tal18G*, suggesting that these genes emerged specifically in the *X. citri* pv. *fuscans* NF2 lineage. Finally, most strains from the *X. citri* pv. *fuscans* fuscans and *X. phaseoli* pv. *phaseoli* NF1 lineages, despite being phylogenetically distant, contained *tal23A* homologues and *tal18H* which is highly suggestive of HGT (Fig. 1). Ks analyses confirmed that *tal18H* has been horizontally transferred between these two lineages and that *tal23A* homologues have been transmitted by HGT between the NF1 lineage and the NF2 lineage. Moreover, plasmid conservation among phylogenetically distant lineages showed that HGT involving both *tal18H* and *tal23A* homologues were not restricted to these *tal* genes alone, and highlight the role of plasmids for these two HGT. These results are reminiscent of studies showing that numerous genes including type three effectors have been horizontally transferred between *X. citri* pv. *fuscans* and *X. phaseoli* pv. *phaseoli* lineages [36, 74]. This also suggests that *tal18H* and *tal23A* homologues have been involved in *Xanthomonas* adaptation to common bean. In particular, *tal23A* homologues were conserved in all strains responsible for CBB, suggesting that they could play a crucial role in *X. citri* pv. *fuscans* and *X. phaseoli* pv. *phaseoli* pathogenicity.

The study of repeats and RVD evolution shed new light on the importance of *tal* genes for the interaction between *Xanthomonas* strains and common bean. The DisTAL tree revealed that TAL23A and TAL18H presented variations in their repeats for aminoacids other than the RVD, resulting in three variants of TAL23A and five variants of TAL18H (Fig. 4a). As RVD sequence is responsible for DNA-binding specificity, these observations suggested that selection pressure acted against RVD diversification in both TAL23A and TAL18H, highlighting the importance of TAL23A and TAL18H functional maintenance in different *X. citri* pv. *fuscans* and *X. phaseoli* pv. *phaseoli* lineages. In particular, TAL18H RVD combination was completely conserved in both NF1 and fuscans lineages, which is in line with TAL18H being involved in *Xanthomonas* adaptation to common bean. To our knowledge, this is the first case of RVD sequence conservation across distant *Xanthomonas* lineages described so far.

FuncTAL analysis and EBE predictions showed that the function of TAL effectors appeared relatively conserved within each *tal* clade (Fig. 4b, Additional file 17). Another interesting observation was that predicted targets of TAL23A homologues and TAL18H both comprised genes encoding an UDP-glycosyltransferase. So far, UDP-glycosyltransferases have not been described as TAL effectors targets [12, 18, 60–67]. UDP-glycosyltransferase transcription has previously been associated with plant resistance to fungi and bacteria [75–77]. Further analyses including mutant construction, phenotyping and transcriptomics are underway to functionally validate the targets of TAL effectors of strains responsible for CBB.

The origins of the strains used for this study gave additional information on *tal* gene evolution in the different *X. citri* pv. *fuscans* and *X. phaseoli* pv. *phaseoli* lineages (Table 1). Indeed, all four lineages have been found in La Réunion Island in 2000 (http://catalogue-cfbp.inra.fr/recherche.php), indicating that sympatry exists among all these lineages in this place, rendering possible further HGT events between strains from these four lineages. Also, in contrast to the NF2 and NF3 lineages, fuscans and NF1 strains have been collected across very distant geographical areas spanning American, European, and African continents. This widespread distribution could have been facilitated by transport of contaminated seeds [78, 79], in frame with agricultural intensification and expansion [46]. Finally, two fuscans strains collected in 1954 and 2009 and two NF1 strains collected in 1966 and 2000 all contained *tal18H* and *tal23A* homologues, indicating that these *tal* genes have been maintained in both lineages for dozens of years. This further highlights the importance of these TAL effectors in these two lineages.

Our finding that plasmidic localisation has played a role in *tal* gene dissemination across phylogenetically distant *Xanthomonas* lineages adapted to common bean shed new light in *tal* genes potential of evolution. Indeed, it appears that two different ways of evolution exist for *tal* genes in *Xanthomonas*. First, it is striking that *X. oryzae* pv. *oryzae* and *X. oryzae* pv. *oryzicola* pathovars rely on numerous (up to 28) chromosomal *tal* genes [29, 68, 80–82]. Also, several cases of convergent targeting of *SWEET* family genes by *X. oryzae* pv. *oryzae* and *X. oryzae* pv. *oryzicola* TAL effectors have been described [12, 58, 61, 83, 84]. Moreover, polymorphism in EBE, or localisation of EBE upstream of *executor* genes led to the resistance of several rice accession [15, 22–25]. Together, these observations in rice suggested that consecutive events of coevolution occurred between rice and *X. oryzae* pv. *oryzae* / *X. oryzae* pv. *oryzicola* TAL effectors. An interpretation to this would be that presence of numerous *tal* genes localised in the chromosome would be an evidence of long-termed co-evolution between TAL effectors and the host plant genome. In sharp contrast with what was observed in *X. oryzae* pv. *oryzae* and *X. oryzae* pv. *oryzicola*, other pathovars rely solely on few *tal* genes localised on plasmids. For example, *X. euvesicatoria* contains only two *tal* genes, *AvrBs3* and *AvrBs4*, both encoded on plasmids [85], while *X. citri* subsp. *citri* bears up to four *pthA* homologues on two different plasmids [86]. In *X. phaseoli* pv. *phaseoli* and *X. citri* pv. *fuscans*, we observed only one to three *tal* genes per strain and the majority was plasmidic. The *tal* genes with chromosomic localisation were the result of a recent duplication in the NF2 lineage, which could be a trace of recent coevolution with common bean. On the other hand, plasmidic TAL effectors were conserved in different lineages and some of them bore the exact same RVD sequence, arguing for a recent horizontal transfer of these *tal* genes. It is tempting to speculate that RVD conservation in horizontally-transferred *tal* genes reflects the fact that the host plant did not have enough time to evolve any resistance against these TAL effectors. An interpretation to this would be that a short number of *tal* genes with plasmidic localisation would be an evidence of short-termed evolution without coevolution between the TAL effector and the host genome. As such, horizontal transfers of *tal* genes can lead to the rapid modification of TAL repertoires, and could participate to the acquisition of novel traits for host adaptation.

## Conclusion

Together, our observations favour a model where plasmidic *tal* genes are able to contribute to host adaptation by being horizontally transferred between distant lineages. Such horizontal gene transfer can take place either on a host plant or on another asymptomatic plant where a broader *Xanthomonas* diversity is expected to live [45]. Because we are not completely aware of this diversity, we may still underestimate the evolutionary potential of TAL effector to circumvent TAL-based resistance engineering strategies. To anticipate such potential and elaborate durable resistances against diseases caused by xanthomonads, it is important to make use of third-generation sequencing strategies on a much broader diversity of *Xanthomonas* strains than what has been made so far, and to explore more in-depth the whole TAL repertoire of xanthomonads.

## Methods

### Bacterial strains, plant material and growing conditions

*Xanthomonas* strains used and sequenced for this study are listed in Table 1. Strains were grown 48 h at 28 °C on TSA medium (17.0 g/L pancreatic digest of casein; 3.0 g/L enzymatic digest of soya bean; 5.0 g/L NaCl; 2.5 g/L K_2_HPO_4_; 2.5 g/L glucose; 15 g/L agar), then 24 h at 28 °C on a 1/10 dilution of TSA medium to obtain fresh grown bacteria. When needed, rifamycin was added at a final concentration of 50 mg/L.

The seeds from common bean cultivar Flavert used in this study were kindly provided by Vilmorin (La Ménitré, France) and were considered free of *X. citri* pv. *fuscans* and *X. phaseoli* pv. *phaseoli* following the analysis of approximately 100,000 seed per lot with standard tests (International Seed Testing Association 2007). Bean plants were grown in plastic square pots (8 cm) containing compost (Neuhaus humin substrat S NF 44–551) in a controlled climatic room under 95% relative humidity with an alternation of 16 h of light at 23 °C and 8 h of darkness at 20 °C until the first trifoliate leaf was fully expanded. Plants were watered every 2 days and supplemented with N-P-K (18:14:18) at 0.3 g/L once a week.

### Pathogenicity assays

The day before inoculation, and for the duration of pathogenicity assays, plants were incubated under 95% relative humidity with an alternation of 16 h of light at 28 °C and 8 h of darkness at 25 °C. Pathogenicity assays were performed with bacterial suspensions calibrated at 1 × 10^7^ CFU/mL in sterile distilled H_2_O. Pathogenicity assays were performed by bathing the first trifoliate leave for 30 s into bacterial suspensions. One trifoliate per plant and three plants per strain were inoculated. Symptoms were scored on the 11th day following inoculation according to the following scale: 0 = no symptoms, 1 = 1 to 50 spots per leaf, 2 = 51 to 200 spots per leaf, necrosis and sagging and 3 = more than 200 spots per leaf, necrosis, sagging or leaf death. Average scores were calculated from the values of three plants per strain. Pathogenicity assays were carried out under quarantine at UMR1345 IRHS, Centre INRA, Beaucouzé, France.

### Genomic DNA extraction, genome sequencing, assembly and annotation

Bacterial cells were scraped from agar plates, suspended in sterile distilled H_2_O and collected by centrifugation. Genomic DNA was extracted with The Wizard® Genomic DNA Purification Kit (Promega) according to manufacturer’s recommendations. Whole genomes were sequenced using the PacBio SMRT technology [87] at Icahn School of Medicine at Mount Sinai (New York, NY). One SMRT cell was used per strain to achieve ~100× coverage. De novo assembling was performed using HGAP assembler [88] version 3.0 (Pacific Biosciences, Menlo Park, CA). Annotation of whole genome assemblies was performed with EuGene-PP version 1.2 automated pipeline [89], using SWISS-PROT as protein and training protein databases (http://www.uniprot.org/). The sequences of *tal* genes were manually retrieved using BLASTN search with the N-ter- and C-ter-encoding regions of *Xfutal1* and *Xfutal2* as queries [37]. In case of indels leading to *tal* pseudogenes, sequence verifications were performed by PCR amplifications using specific primers on genomic DNA, followed by Sanger sequencing of PCR products (Additional file 18).

### Phylogenetic analyses and detection of recombination

The phylogeny of organisms was performed using CVTree version 4.2.1 [90] on the whole predicted proteome of strains listed in Table 1, plus 31 proteomes available in public database that belong to *Xanthomonas* pathovars where *tal* genes have previously been described (Additional file 5).

For *tal* genes phylogeny, we used the *tal* genes from strains listed in Table 1, plus 151 *tal* genes encoding unique RVD sequences, available in public databases and sequenced by SMRT sequencing or functionally validated (Additional file 5). The amino acid sequences of the N-ter and C-ter regions of these TAL effectors were aligned with MAFFT version 7 using the L-ins-I strategy [91], then this alignment was used as template for generating a codon-based nucleotide alignment with RevTrans 2.0 [92]. For both alignments, the best model of evolution determined using ModelTest 3.7 in Paup was the TVM + I + G model. Under this model, Maximum Likelihood (ML) phylogenetic trees were generated using PhyML 3.0 version 20,110,919 [93] and bootstraps analyses were done with 1000 iterations. Trees were visualised and manually edited with Mega 7.0.14 [94].

For detecting recombination, two additional trees constructed using nucleotide alignments of the N-ter- or C-ter-encoding regions of *tal* genes from *X. citri* pv. *fuscans*, *X. citri* pv. *aurantifolii*, *X. phaseoli* pv. *phaseoli*, *X. phaseoli* pv. *manihotis* strains and a *tal* gene from *X. translucens* pv. *undulosa* as outgroup were produced using the aforementioned method. For the N-ter -encoding region tree, the model of evolution was K81uf + I and for the C-ter-encoding region tree, the model of evolution was TVM + G (Additional file 19). The Kishino-Hasegawa-Templeton test [47] implemented in the DNAML program from PHYLIP version 3.69 [95] was used to test the congruence of tree topologies. Also, nucleotide alignments from both N-ter- and C-ter-encoding regions of *tal* genes from *X. citri* pv. *fuscans*, *X. citri* pv. *aurantifolii*, *X. phaseoli* pv. *phaseoli* and *X. phaseoli* pv. *manihotis* strains were concatenated. Detection of potential recombination events were searched for on the three nucleotide alignments (separated N-ter- and C-ter-encoding regions, and concatenated) using a set of seven nonparametric detection programs implemented in RDP version 4 beta 69 [48]: RDP [96]; Geneconv [97]; Bootscan [98]; MaxChi [99]; Chimera [100]; SiScan [101] and 3Seq [102]. Default parameter settings were used for each method except as follows: RDP (internal reference sequence); Bootscan (window size = 150, neighbour joining trees, 200 bootstrap replicates, 95% cutoff, Jin and Nei model, variation coefficient = 2). For general settings, the maximum *P* value for accepting recombination was set at 0.001 after a Bonferroni correction. Only recombination events retrieved by at least three programs with a *P* value <0.001 were considered.

### Average nucleotide identity calculations

Pairwise comparisons of *X. citri* pv. *fuscans* and *X. phaseoli* pv. *phaseoli* genome and plasmids nucleotide identities were performed by calculating the Average Nucleotide Identity by using BLASTn with JSpecies using default parameters [103] (Additional file 6, Additional file 8, Additional file 9).

### Ks calculations

Ks was estimated on nucleotide alignments of N-ter- and C-ter-encoding regions of *tal* genes from *X. citri* pv. *aurantifolii*, *X. citri* pv. *fuscans*, *X. phaseoli* pv. *manihotis* and *X. phaseoli* pv. *phaseoli* strains, as well as on a concatenated alignment of seven previously described housekeeping genes from *X. oryzae*, *X. citri*, *X. phaseoli*, and *X. euvesicatoria* species [46]. Alignments and recombination analyses were done as described in the “Phylogenetic analyses and detection of recombination” section. After removal of recombinant regions, Ks was determined using DNAsp version 5 with default parameters [104]. On the same alignment, codon usage bias was calculated using DNAsp version 5 and no major difference was observed between the different *Xanthomonas* species (data not shown). Also Tajima’s and Fu and Li’s relative rate test were performed with DNAsp version 5 and no significant divergence from neutrality was retrieved (*P* > 0.05).

### Repeats and RVD analyses

Amino acid sequences of every repeats from TAL effectors of *X. citri* pv. *fuscans*, *X. citri* pv. *aurantifolii*, *X. phaseoli* pv. *phaseoli* and *X. phaseoli* pv. *manihotis* strains were manually extracted and aligned using MAFFT version 7 [91], after RVD exclusion. Then, unique repeats were searched, and repeats found multiple times were counted using Geneious version 9.1.3 [105]. For Fig. 1, amino acid RVD sequence of TAL effectors from *X. citri* pv. *fuscans* and *X. phaseoli* pv. *phaseoli* strains were extracted and an alignment was performed with MAFFT version 7 [91].

The evolution of TAL effector repeat regions and RVD sequences were analysed using the QueTAL suite [57]. First, amino acid sequences of repeat regions from TAL effectors of *X. citri* pv. *fuscans*, *X. citri* pv. *aurantifolii*, *X. phaseoli* pv. *phaseoli*, *X. phaseoli* pv. *manihotis* strains were extracted and aligned manually according to the QueTAL’s instructions, using TAL effectors from *X. translucens* pv. *undulosa* as outgroup for the DisTAL analysis and no outgroup for the FuncTAL analysis. Then, these alignments were used to construct a distance tree of TAL effector repeat regions using DisTAL version 1.1 and a distance tree of RVD sequences using FuncTAL version 1.1 [57]. DisTAL program considers repeat sequences without RVD whereas FuncTAL program only considers RVD sequences.

With the nine unique RVD combinations, predictions of EBE were performed using TALVEZ version 3.2 with correction matrix starting from the 14th RVD [11] on the common bean promoterome consisting of the 3 kb region upstream of the transcriptional start of each transcript annotated on the genome of common bean genotype G19833 version 2.1 [59] available on Phytozome 12 (https://phytozome.jgi.doe.gov/).

## Additional files

Additional file 1: Table S1. Correction of sequences from *tal* pseudogenes containing indels in the SMRT sequence. (XLSX 10 kb)

Additional file 2: Table S2. Amino acid sequences of TAL effector from the 17 strains responsible for CBB obtained in this study. (DOCX 19 kb)

Additional file 3: Figure S1. Gel photograph showing the sizes obtained for *tal23A_CFBP4885* (*Xfutal1*) and *tal18H_CFBP4885* (*Xfutal2*) after PCR amplification. Amplification was performed using XapF2 and XapR primers on plasmidic DNA from the CFBP4885 strain (Additional file 18: **Table S11**). Expected PCR product sizes for *tal23A_CFBP4885* and *tal18H_CFBP4885* were 4604 bp and 3074 bp for Sanger sequencing of the entire genome, respectively, and 3686 bp and 3176 bp for SMRT sequencing of the entire genome, respectively. (DOCX 2788 kb)

Additional file 4: Figure S2. RVD frequencies in TAL effectors from the 17 *X. citri* pv. *fuscans* and *X. phaseoli* pv. *phaseoli* strains used in this study. (DOCX 20 kb)

Additional file 5: Table S3. References for previously published *tal* genes and whole genome sequences from *Xanthomonas* spp. strains used in this study. (XLSX 19 kb)

Additional file 6: Table S4. Average nucleotide identity and percentage of alignment between whole genome sequences from 17 *X. citri* pv. *fuscans* and *X. phaseoli* pv. *phaseoli* strains. BLAST calculation of average nucleotide identity (ANIb) and alignment percentages were estimated using Jspecies (Richter and Rossello-Mora, 2009). (XLSX 15 kb)

Additional file 7: Figure S3. Phylogenetic trees of N-ter and C-ter-encoding regions of *tal* genes with sequences published by Aritua et al. (2015). Bootstrap values greater than 50% (100 replicates) are shown and horizontal scale bars represent the number of nucleotide substitutions per site. The *tal* genes from the *X. citri* pv. *fuscans* genetic lineages fuscans, NF2 and NF3 are indicated in red, *X. phaseoli* pv. *phaseoli* NF1 lineage in blue, *X. citri* pv. *aurantifolii* in pink and *X. phaseoli* pv. *manihotis* in purple. The sequences published by Aritua et al. (2015) are in bold. Both ML trees were constructed using *tal* genes from *X. translucens* pv. *undulosa* XT4699 as outgroups. **a** ML tree constructed on a nucleotide alignment of the N-ter-encoding region of *tal* genes. **b** ML tree constructed on a nucleotide alignment of the C-ter-encoding region of *tal* genes. (PPTX 137 kb)

Additional file 8: Figure S4. Circular representation of plasmids A and C. Genomic sequences were compared and converted in a graphical map using CGView (Grant et al., 2012) with strain CFBP4885 as reference. Colours differ according to identity percentage (see legend). Strains order, ANIb and alignment percentages are indicated in the center of each graphical map. Localisation of *tal* genes are highlighted by green zones. Absence of *tal* genes in plasmid A from strains CFBP6164 and CFBP6546R is indicated by a black cross. (PDF 444 kb)

Additional file 9: Table S5. Average nucleotide identity and percentage of alignment between sequences of plasmid A from 17 *X. citri* pv. *fuscans* and *X. phaseoli* pv. *phaseoli* strains. BLAST calculation of average nucleotide identity (ANIb) and alignment percentages were estimated using Jspecies (Richter and Rossello-Mora, 2009). (XLSX 14 kb)

Additional file 10: Figure S5. Circular representation of the regions shared by phylogenetically distant lineages in plasmids A and C. Genomic sequences were compared and converted in a graphical map using CGView (Grant et al., 2012). Colours differ according to identity percentage (see legend). Regions shared by both strains are surrounded by a black line. Numbers represent genes conserved in both strains (see Additional files 11 and 13 for more details). **a** Plasmids A from strains CFBP6988R (outer circle = reference) and CFBP6546R (inner circle). **b** Plasmids C from strains CFBP6166 (outer circle = reference) and CFBP6982 (inner circle). (PDF 168 kb)

Additional file 11: Table S6. Genes localized in Plasmid A shared by strains CFBP6988R and CFBP6546R. Please refer to Additional file 10a for graphic representation of the data. (XLSX 15 kb)

Additional file 12: Table S7. Average nucleotide identity and percentage of alignment between sequences of plasmid C from 8 *X. citri* pv. *fuscans* fuscans and *X. phaseoli* pv. *phaseoli* NF1 strains. BLAST calculation of average nucleotide identity (ANIb) and alignment percentages were estimated using Jspecies (Richter and Rossello-Mora, 2009). (XLSX 11 kb)

Additional file 13: Table S8. Genes localized in Plasmid C shared by strains CFBP6982 and CFBP6166. Please refer to Additional file 10b for graphic representation of the data. (XLSX 15 kb)

Additional file 14: Figure S6. Duplications and movements of *tal* genes associated with IS3 and Tn3-like transposons. Arrows represent *tal* genes (yellow), IS3 sequences (orange) or Tn3-like transposons (dark blue). Arrowheads correspond to truncated IS3 (orange) or Tn3-like transposon (dark blue). Striped areas correspond to conserved regions. Dashed lines represent the borders of duplication events between different regions of a single molecule. **a** Movement of *tal23A* homologues from the plasmid A to the chromosome associated with IS3 sequences. **b** Duplication of *tal20F* and *tal18G* associated with Tn3-like transposons. **c.** Duplication of *tal18H_CFBP6164* associated with IS3 sequences in strain CFBP6164. (PPTX 74 kb)

Additional file 15: Table S9. Repeats found within all TAL effectors from *X. citri* pv. *fuscans*, *X. phaseoli* pv. *phaseoli*, *X. citri* pv. *aurantifolii* and *X. phaseoli* pv. *manihotis* strains. Sequences were compared after removal of RVD. Xcf = *X. citri* pv. *fuscans*; Xpp = *X. phaseoli* pv. *phaseoli*; Xca = *X. citri* pv. *aurantifolii*; Xpm = *X. phaseoli* pv. *manihotis*. A coloured line indicates that the repeat is shared between TAL effectors from different groups, one colour being associated with a single repeat and corresponding to the colour of repeats in Additional file 16: **Figure S7**. (XLSX 12 kb)

Additional file 16: Figure S7. Repeats conserved and shared between different TAL effectors from *X. citri* pv. *fuscans*, *X. phaseoli* pv. *phaseoli*, *X. citri* pv. *aurantifolii* and *X. phaseoli* pv. *manihotis* strains. Repeats conserved between TAL effectors from different groups are indicated by the same colour (red, purple, green, blue or yellow). Half-coloured boxes indicate that polymorphism exist among TAL effectors homologues from different strains. Please see Additional file 15: **Table S9** for more detailed information. (PPTX 69 kb)

Additional file 17: Table S10. Top 10 predicted targets for the nine TAL effectors with unique RVD sequences retrieved in 17 *X. citri* pv. *fuscans* and *X. phaseoli* pv. *phaseoli* strains. Target names correspond to locus tags from the *P. vulgaris* genome available at https://phytozome.jgi.doe.gov/. Similar targets predicted for different TAL effectors are highlighted by colours corresponding to the predicted function of the target (see the legend below). (XLSX 17 kb)

Additional file 18: Table S11. Primers used in this study. (DOCX 19 kb)

Additional file 19: Figure S8. Phylogenetic trees of N-ter- and C-ter-encoding regions of *tal* genes used for the Kishino-Hasegawa-Templeton test. Bootstrap values greater than 50% are shown for 1000 replicates and horizontal scale bars represent the number of nucleotide substitutions per site. **a** ML tree constructed on a nucleotide alignment of the N-ter-encoding region of *tal* genes from *X. citri* pv. *fuscans*, *X. citri* pv. *aurantifolii*, *X. phaseoli* pv. *phaseoli* and *X. phaseoli* pv. *manihotis* strains using a *tal* gene from *X. translucens* pv. *undulosa* XT4699 as outgroup. **b** ML tree constructed on a nucleotide alignment of the C-ter-encoding region of *tal* genes from *X. citri* pv. *fuscans*, *X. citri* pv. *aurantifolii*, *X. phaseoli* pv. *phaseoli* and *X. phaseoli* pv. *manihotis* strains using a *tal* gene from *X. translucens* pv. *undulosa* XT4699 as outgroup. (PPTX 56 kb)

## Abbreviations

ANI: Average nucleotide identity
CBB: Common bacterial blight
CFBP: Collection Française de Bactéries associées aux Plantes
CFU: Colony forming unit
EBE: Effector binding element
HGT: Horizontal gene transfer
IS: Insertion sequence
ML: Maximum likelihood
NF: Non-fuscous
PCR: Polymerase chain reaction
RVD: Repeat variable diresidue
SMRT: Single molecule, real-time
TAL: Transcription activator-like
