# Supplementary material for: Xanthomonas adaptation to common bean is associated with horizontal transfers of genes encoding TAL effectors
Source: BMC Genomics. 2017 Aug 30;18:670. doi: 10.1186/s12864-017-4087-6 (PMC5577687; doi:10.1186/s12864-017-4087-6)
Supplement: Supplementary file 2 — Amino acid sequences of TAL effector from the 17 strains responsible for CBB obtained in this study. (DOCX 19 kb) [file 12864_2017_4087_MOESM2_ESM.docx]

**Additional file 2: Table S2**. Amino acid sequences of TAL effectors from the 17 strains responsible for CBB obtained in this study.

| TAL23A_CFBP4885 |
| --- |
| MDPIRPRTPSPAHELLAGPQPDRVQPQPTADRGGAPPAGSPLDGLPARRTMSRTRLPSPPAPLPAFSAGSFSDLLRQFDPSLLDTSLFDSMSAFGAPHTEAASGEGDEVQSGLRAADDPQATVQVAVTAARPPRAKPVPRRRAAHTSDASPAGQVDLCTLGYSQQQQEKIKLKARSTVAQHHEALIGHGFTRAQIVALSQHPAALGTVAVKYQVMIAALPEATHEDIVGVGKQWSGARALEALLTVSGELRGPPLQLDTGQLLKIAKRGGVTAVEAVHAWRNALTGAPLNLTPDQVVAIASNIGGKQALETVQRLLPVLCEQHGLTPDQVVAIASNGGGKQALETVQRLLPVLCEQHGLTPDQVVAIASNIGGKQALETVQRLLPVLCEQHGLTPEQVVAIASNGGGKQALETVQRLLPVLCQAHGLTPDQVVAIASNIGGKQALETVQRLLPVLCEQHGLTPDQVVAIASNNGGKQALETVQRLLPVLCEQHGLTPDQVVAIASNGGGKQALETVQRLLPVLCEQHGLTREQVVAIASNNGGKQALETVQRLLPVLCQAHGLTPEQVVAIASHDGGKQALETLQRLLPVLCEQHGLTREQVVAIASHDGGKQALETLQRLLPVLCEQHGLTREQVVAIASNNGGKQALETVQRLLPVLCEQHGLTPAQVVAIASNNGGKQALETVQRLLPVLCEQHGLTPEQVVAIASHDGGKQALETVQRLLPVLCEQHGLTPEQVVAIASNIGGKQALETVQRLLPVLCEQHGLTPDQVVAIASHDGGKQALETVQRLLPVLCEQHGLTREQVVAIASNIGGKQALETVQRLLPVLCEQHGLTPEQVVAIASHDGGKQALETVQRLLPVLCEQHGLTPEQVVAIASHDGGKQALETVQRLLPVLCQAHGLTPEQVVAIASHDGGKQALETVQRLLPVLCQAHGLTPEQVVAIASHDGGKQALETVQRLLPVLCEQHGLTREQVVAIASHDGGKQALETVQRLLPVLCQAHGLTPEQVVAIASNGGGKQALETVQRLLPVLCEQHGLTPAQVVAIASNGGGKQALESIFAQLSRPDQALAALTNDHLVALACLGGRPALEAVKKGLPHAPTLIKRTNRRLPERTSHRVADHAQVARVLGFFQCHSHPAQAFDEAMTQFGMSRHGLLQLFRRVGVTELEARSGTLPPAPQRWHRILQASGMKRAEPSGASAQTPDQASLHAFADALERELDAPSPIDRAGQALASSSRKRSRSESSVTGSFAQQAVEVRVPEQHEGLHFPPLSWGVKRPRTRIGGGLPDPGTPMDADLAASSTMIWEQDAAPFVGEEDDFPAFNEEEMAWLMELFPQ |
| TAL18H_CFBP4885 |
| MDPIRPRAPSPAHELLAEPQPDRVQPQPTADRGGSPPAGSPLDGLPARRTMSRTRLPSPPAPLPAFSAGSFRDLLRQFDPSLLDTSLFDSMSAFGAPHTEAASGEGDEVQSGLRAADDPQATVQVAVTAARPPRAKPAPRRRAAHTSDASPAGQVDLCTLGYSQQQQEKIKLKARPIVAQHHEALIGHGFTRAHIVALSQHPAALGTVAVKYQAMIAALPEATHEDIVGVGKQWSGARALEALLTVSGELRGPPLQLDTGQLLKIAKRGGVTAVEAVHAWRNALTGVPLNLTPDQVVAIASNIGGKQALETVQLLLPVLCEQHGLTPDQVVAIASNGGGKQALETVQRLLPVLCKDHGLTPAQVVAIANHDGGKQALETVQRLLPVLCKDHGLTPAQVVAIASNGGGKQALETVEQLLPVLCKDHGLTPDQVVAIANHDGGKPALETVQRLLPVLCQELGLTPDQVVAIASNIGGKQALETVQRLLPVLCQELGLTPDQVVAIASNGGAKQALETVQRLLPVLCQELGLTPAQVVAIASNIGGKQALETVQRLLPVLCEQHGLTPDQVVAIASHDGGKQALETVERLLPVLCQELGMTLAQVVAIASHYGGKQALEAVQRLLPVLCQELGLTPDQVVAIASNNGGKQALETVQRLLPVLCEQHGLTPDQVVAIASNGGKQALETVQRLLPVLCEQHGLTPDQVVAIASHDGGKQALETVERLLPVLCQELGMTLAQVVAIASNGGGKQALETVERLLPVLCQELGMTLAQVVAIASHYGGKQALEAVQRLLPVLCQDLGLTPDQVVAIASNNGGKQALETVQRLLPVLCEQHGLTPDQVVAIASHDGGKQALEAVQRLLPVLCQDHGLNPDQVVAIASNGGGRQALESIFAQLSRPDQALAALTNDHLVALACLGGCPALEAVKKGLPHALTLIKRTSRRLPERTSHRVADHAQVARVLGFFQCHSHPAQAFDEAMAQFAMSRHGLLQLFRRVGVTELEACNGTLPPASQRWHRILQASGVRTATPSRASAQTPDQASLDAFADALERGLDALSPIDQAVQAQASSRRKRSRSESSVTRSSAHYAVEVPVREQHAALDSLPPSWGAKRPRTRIGGGLADPGTPMHGDLAASSTVAWEHDAAPFAAAEAGDFPAFNDEEIAWLMELFPQ |

| TAL22B_CFBP6165 |
| --- |
| MDPIRPRTPSPAHELLAGPQPDRVQPQPTADRGGAPPAGSPLDGLPARRTMSRTRLPSPPAPLPAFSAGSFSDLLRQFDPSLLDTSLFDSMSAFGAPHTEAASGEGDEVQSGLRAADDPQATVQVAVTAARPPRAKPVPRRRAAHTSDASPAGQVDLCTLGYSQQQQEKIKLKARSTVAQHHEALIGHGFTRAQIVALSQHPAALGTVAVKYQVMIAALPEATHEDIVGVGKQWSGARALEALLTVSGELRGPPLQLDTGQLLKIAKRGGVTAVEAVHAWRNALTGAPLNLTPDQVVAIASNIGGKQALETVQRLLPVLCEQHGLTPDQVVAIASNGGGKQALETVQRLLPVLCEQHGLTPDQVVAIASNIGGKQALETVQRLLPVLCEQHGLTPDQVVAIASNGGGKQALETVQRLLPVLCEQHGLTPDQVVAIASNIGGKQALETVQRLLPVLCEQHGLTPDQVVAIASNNGGKQALETVQRLLPVLCEQHGLTPDQVVAIASNGGGKQALETVQRLLPVLCEQHGLTREQVVAIASNNGGKQALETVQRLLPVLCQAHGLTPEQVVAIASHDGGKQALETVQRLLPVLCEQHGLTREQVVAIASNNGGKQALETLQRLLPVLCEQHGLTREQVVAIASNNGGKQALETVQRLLPVLCEQHGLTPAQVVAIASNNGGKQALETVQRLLPVLCEQHGLTPEQVVAIASHDGGKQALETVQRLLPVLCEQHGLTPEQVVAIASNIGGKQALETVQRLLPVLCEQHGLTPDQVVAIASHDGGKQALETVQRLLPVLCEQHGLTREQVVAIASNIGGKQALETVQRLLPVLCQAHGLTPEQVVAIASHDGGKQALETVQRLLPVLCQAHGLTPEQVVAIASHDGGKQALETVQRLLPVLCQAHGLTPEQVVAIASHDGGKQALETVQRLLPVLCQAHGLTPEQVVAIASHDGGKQALETVQRLLPVLCEQHGLTREQVVAIASHDGGKQALETVQRLLPVLCQAHGLTPEQVVAIASNGGGKQALESIFAQLSRPDQALAALTNDHLVALACLGGRPALEAVKKGLPHAPTLIKRTNRRLPERTSHRVADHAQVARVLGFFQCHSHPAQAFDEAMTQFGMSRHGLLQLFRRVGVTELEARSGTLPPAPQRWHRILQASGMKRAEPSGASAQTPDQASLHAFADALERELDAPSPIDRAGQALASSSRKRSRSESSVTGSFAQQAVEVRVPEQHEGLHFPPLSWGVKRPRTRIGGGLPDPGTPMDADLAASSTMIWEQDAAPFVGEEDDFPAFNEEEMAWLMELFPQ |

| TAL23A_CFBP6166 |
| --- |
| MDPIRPRTPSPAHELLAGPQPDRVQPQPTADRGGAPPAGSPLDGLPARRTMSRTRLPSPPAPLPAFSAGSFSDLLRQFDPSLLDTSLFDSMSAFGAPHTEAASGEGDEVQSGLRAADDPQATVQVAVTAARPPRAKPVPRRRAAHTSDASPAGQVDLCTLGYSQQQQEKIKLKARSTVAQHHEALIGHGFTRAQIVALSQHPAALGTVAVKYQVMIAALPEATHEDIVGVGKQWSGARALEALLTVSGELRGPPLQLDTGQLLKIAKRGGVTAVEAVHAWRNALTGAPLNLTPDQVVAIASNIGGKQALETVQRLLPVLCEQHGLTPDQVVAIASNGGGKQALETVQRLLPVLCEQHGLTPDQVVAIASNIGGKQALETVQRLLPVLCEQHGLTPEQVVAIASNGGGKQALETVQRLLPVLCQAHGLTPDQVVAIASNIGGKQALETVQRLLPVLCEQHGLTPDQVVAIASNNGGKQALETVQRLLPVLCEQHGLTPDQVVAIASNGGGKQALETVQRLLPVLCEQHGLTREQVVAIASNNGGKQALETVQRLLPVLCQAHGLTPEQVVAIASHDGGKQALETLQRLLPVLCEQHGLTREQVVAIASHDGGKQALETLQRLLPVLCEQHGLTREQVVAIASNNGGKQALETVQRLLPVLCEQHGLTPAQVVAIASNNGGKQALETVQRLLPVLCEQHGLTPEQVVAIASHDGGKQALETVQRLLPVLCEQHGLTPEQVVAIASNIGGKQALETVQRLLPVLCEQHGLTPDQVVAIASHDGGKQALETVQRLLPVLCEQHGLTREQVVAIASNIGGKQALETVQRLLPVLCEQHGLTPEQVVAIASHDGGKQALETVQRLLPVLCEQHGLTPEQVVAIASHDGGKQALETVQRLLPVLCQAHGLTPEQVVAIASHDGGKQALETVQRLLPVLCQAHGLTPEQVVAIASHDGGKQALETVQRLLPVLCEQHGLTREQVVAIASHDGGKQALETVQRLLPVLCQAHGLTPEQVVAIASNGGGKQALETVQRLLPVLCEQHGLTPAQVVAIASNGGGKQALESIFAQLSRPDQALAALTNDHLVALACLGGRPALEAVKKGLPHAPTLIKRTNRRLPERTSHRVADHAQVARVLGFFQCHSHPAQAFDEAMTQFGMSRHGLLQLFRRVGVTELEARSGTLPPAPQRWHRILQASGMKRAEPSGASAQTPDQASLHAFADALERELDAPSPIDRAGQALASSSRKRSRSESSVTGSFAQQAVEVRVPEQHEGLHFPPLSWGVKRPRTRIGGGLPDPGTPMDADLAASSTMIWEQDAAPFVGEEDDFPAFNEEEMAWLMELFPQ |
| TAL18H_CFBP6166 |
| MDPIRPRAPSPAHELLAEPQPDRVQPQPTADRGGSPPAGSPLDGLPARRTMSRTRLPSPPAPLPAFSAGSFRDLLRQFDPSLLDTSLFDSMSAFGAPHTEAASGEGDEVQSGLRAADDPQATVQVAVTAARPPRAKPAPRRRAAHTSDASPAGQVDLCTLGYSQQQQEKIKLKARPIVAQHHEALIGHGFTRAHIVALSQHPAALGTVAVKYQAMIAALPEATHEDIVGVGKQWSGARALEALLTVSGELRGPPLQLDTGQLLKIAKRGGVTAVEAVHAWRNALTGVPLNLTPDQVVAIASNIGGKQALETVQLLLPVLCEQHGLTPDQVVAIASNGGGKQALETVQRLLPVLCKDHGLTPAQVVAIANHDGGKQALETVQRLLPVLCKDHGLTPAQVVAIASNGGGKQALETVEQLLPVLCKDHGLTPDQVVAIANHDGGKPALETVQRLLPVLCQELGLTPDQVVAIASNIGGKQALETVQRLLPVLCQELGLTPDQVVAIASNGGAKQALETVQRLLPVLCQELGLTPAQVVAIASNIGGKQALETVQRLLPVLCEQHGLTPDQVVAIASHDGGKQALETVERLLPVLCQELGMTLAQVVAIASHYGGKQALEAVQRLLPVLCQELGLTPDQVVAIASNNGGKQALETVQRLLPVLCEQHGLTPDQVVAIASNGGKQALETVQRLLPVLCEQHGLTPDQVVAIASHDGGKQALETVERLLPVLCQELGMTLAQVVAIASNGGGKQALETVERLLPVLCQELGMTLAQVVAIASHYGGKQALEAVQRLLPVLCQDLGLTPDQVVAIASNNGGKQALETVQRLLPVLCEQHGLTPDQVVAIASHDGGKQALEAVQRLLPVLCQDHGLNPDQVVAIASNGGGRQALESIFAQLSRPDQALAALTNDHLVALACLGGCPALEAVKKGLPHALTLIKRTSRRLPERTSHRVADHAQVARVLGFFQCHSHPAQAFDEAMAQFAMSRHGLLQLFRRVGVTELEACNGTLPPASQRWHRILQASGVRTATPSRASAQTPDQASLDAFADALERGLDALSPIDQAVQAQASSRRKRSRSESSVTRSSAHYAVEVPVREQHAALDSLPPSWGAKRPRTRIGGGLADPGTPMHGDLAASSTVAWEHDAAPFAAAEAGDFPAFNDEEIAWLMELFPQ |

| TAL23A_CFBP6167 |
| --- |
| MDPIRPRTPSPAHELLAGPQPDRVQPQPTADRGGAPPAGSPLDGLPARRTMSRTRLPSPPAPLPAFSAGSFSDLLRQFDPSLLDTSLFDSMSAFGAPHTEAASGEGDEVQSGLRAADDPQATVQVAVTAARPPRAKPVPRRRAAHTSDASPAGQVDLCTLGYSQQQQEKIKLKARSTVAQHHEALIGHGFTRAQIVALSQHPAALGTVAVKYQVMIAALPEATHEDIVGVGKQWSGARALEALLTVSGELRGPPLQLDTGQLLKIAKRGGVTAVEAVHAWRNALTGAPLNLTPDQVVAIASNIGGKQALETVQRLLPVLCEQHGLTPDQVVAIASNGGGKQALETVQRLLPVLCEQHGLTPDQVVAIASNIGGKQALETVQRLLPVLCEQHGLTPEQVVAIASNGGGKQALETVQRLLPVLCQAHGLTPDQVVAIASNIGGKQALETVQRLLPVLCEQHGLTPDQVVAIASNNGGKQALETVQRLLPVLCEQHGLTPDQVVAIASNGGGKQALETVQRLLPVLCEQHGLTREQVVAIASNNGGKQALETVQRLLPVLCQAHGLTPEQVVAIASHDGGKQALETLQRLLPVLCEQHGLTREQVVAIASHDGGKQALETLQRLLPVLCEQHGLTREQVVAIASNNGGKQALETVQRLLPVLCEQHGLTPAQVVAIASNNGGKQALETVQRLLPVLCEQHGLTPEQVVAIASHDGGKQALETVQRLLPVLCEQHGLTPEQVVAIASNIGGKQALETVQRLLPVLCEQHGLTPDQVVAIASHDGGKQALETVQRLLPVLCEQHGLTREQVVAIASNIGGKQALETVQRLLPVLCEQHGLTPEQVVAIASHDGGKQALETVQRLLPVLCEQHGLTPEQVVAIASHDGGKQALETVQRLLPVLCQAHGLTPEQVVAIASHDGGKQALETVQRLLPVLCQAHGLTPEQVVAIASHDGGKQALETVQRLLPVLCEQHGLTREQVVAIASHDGGKQALETVQRLLPVLCQAHGLTPEQVVAIASNGGGKQALETVQRLLPVLCEQHGLTPAQVVAIASNGGGKQALESIFAQLSRPDQALAALTNDHLVALACLGGRPALEAVKKGLPHAPTLIKRTNRRLPERTSHRVADHAQVARVLGFFQCHSHPAQAFDEAMTQFGMSRHGLLQLFRRVGVTELEARSGTLPPAPQRWHRILQASGMKRAEPSGASAQTPDQASLHAFADALERELDAPSPIDRAGQALASSSRKRSRSESSVTGSFAQQAVEVRVPEQHEGLHFPPLSWGVKRPRTRIGGGLPDPGTPMDADLAASSTMIWEQDAAPFVGEEDDFPAFNEEEMAWLMELFPQ |
| TAL18H_CFBP6167 |
| MDPIRPRAPSPAHELLAEPQPDRVQPQPTADRGGSPPAGSPLDGLPARRTMSRTRLPSPPAPLPAFSAGSFRDLLRQFDPSLLDTSLFDSMSAFGAPHTEAASGEGDEVQSGLRAADDPQATVQVAVTAARPPRAKPAPRRRAAHTSDASPAGQVDLCTLGYSQQQQEKIKLKARPIVAQHHEALIGHGFTRAHIVALSQHPAALGTVAVKYQAMIAALPEATHEDIVGVGKQWSGARALEALLTVSGELRGPPLQLDTGQLLKIAKRGGVTAVEAVHAWRNALTGVPLNLTPDQVVAIASNIGGKQALETVQLLLPVLCEQHGLTPDQVVAIASNGGGKQALETVQRLLPVLCKDHGLTPAQVVAIANHDGGKQALETVQRLLPVLCKDHGLTPAQVVAIASNGGGKQALETVEQLLPVLCKDHGLTPDQVVAIANHDGGKPALETVQRLLPVLCQELGLTPDQVVAIASNIGGKQALETVQRLLPVLCEQHGLTPDQVVAIASNGGAKQALETVQRLLPVLCQELGLTPAQVVAIASNIGGKQALETVQRLLPVLCEQHGLTPEQVVAIASHDGGKQALETVERLLPVLCQELGMTLAQVVAIASHYGGKQALEAVQRLLPVLCQELGLTPDQVVAIASNNGGKQALETVQRLLPVLCEQHGLTPDQVVAIASNGGKQALETVQRLLPVLCEQHGLTPDQVVAIASHDGGKQALETVERLLPVLCQELGMTLAQVVAIASNGGGKQALETVERLLPVLCQELGMTLAQVVAIASHYGGKQALEAVQRLLPVLCQDLGLTPDQVVAIASNNGGKQALETVQRLLPVLCEQHGLTPDQVVAIASHDGGKQALEAVQRLLPVLCQDHGLNPDQVVAIASNGGGRQALESIFAQLSRPDQALAALTNDHLVALACLGGCPALEAVKKGLPHALTLIKRTSRRLPERTSHRVADHAQVARVLGFFQCHSHPAQAFDEAMAQFAMSRHGLLQLFRRVGVTELEACNGTLPPASQRWHRILQASGVRTATPSRASAQTPDQASLDAFADALERGLDALSPIDQAVQAQASSRRKRSRSESSVTRSSAHYAVEVPVREQHAALDSLPPSWGAKRPRTRIGGGLADPGTPMHGDLAASSTVAWEHDAAPFAAAEAGDFPAFNDEEIAWLMELFPQ |

| TAL23A_CFBP6975 |
| --- |
| MDPIRPRTPSPAHELLAGPQPDRVQPQPTADRGGAPPAGSPLDGLPARRTMSRTRLPSPPAPLPAFSAGSFSDLLRQFDPSLLDTSLFDSMSAFGAPHTEAASGEGDEVQSGLRAADDPQATVQVAVTAARPPRAKPVPRRRAAHTSDASPAGQVDLCTLGYSQQQQEKIKLKARSTVAQHHEALIGHGFTRAQIVALSQHPAALGTVAVKYQVMIAALPEATHEDIVGVGKQWSGARALEALLTVSGELRGPPLQLDTGQLLKIAKRGGVTAVEAVHAWRNALTGAPLNLTPDQVVAIASNIGGKQALETVQRLLPVLCEQHGLTPDQVVAIASNGGGKQALETVQRLLPVLCEQHGLTPDQVVAIASNIGGKQALETVQRLLPVLCEQHGLTPEQVVAIASNGGGKQALETVQRLLPVLCQAHGLTPDQVVAIASNIGGKQALETVQRLLPVLCEQHGLTPDQVVAIASNNGGKQALETVQRLLPVLCEQHGLTPDQVVAIASNGGGKQALETVQRLLPVLCEQHGLTREQVVAIASNNGGKQALETVQRLLPVLCQAHGLTPEQVVAIASHDGGKQALETLQRLLPVLCEQHGLTREQVVAIASHDGGKQALETLQRLLPVLCEQHGLTREQVVAIASNNGGKQALETVQRLLPVLCEQHGLTPAQVVAIASNNGGKQALETVQRLLPVLCQAHGLTPEQVVAIASHDGGKQALETVQRLLPVLCEQHGLTPEQVVAIASNIGGKQALETVQRLLPVLCEQHGLTPDQVVAIASHDGGKQALETVQRLLPVLCEQHGLTREQVVAIASNIGGKQALETVQRLLPVLCEQHGLTPEQVVAIASHDGGKQALETVQRLLPVLCEQHGLTPEQVVAIASHDGGKQALETVQRLLPVLCQAHGLTPEQVVAIASHDGGKQALETVQRLLPVLCQAHGLTPEQVVAIASHDGGKQALETVQRLLPVLCEQHGLTREQVVAIASHDGGKQALETVQRLLPVLCQAHGLTPEQVVAIASNGGGKQALETVQRLLPVLCEQHGLTPAQVVAIASNGGGKQALESIFAQLSRPDQALAALTNDHLVALACLGGRPALEAVKKGLPHAPTLIKRTNRRLPERTSHRVADHAQVARVLGFFQCHSHPAQAFDEAMTQFGMSRHGLLQLFRRVGVTELEARSGTLPPAPQRWHRILQASGMKRAEPSGASAQTPDQASLHAFADALERELDAPSPIDRAGQALASSSRKRSRSESSVTGSFAQQAVEVRVPEQHEGLHFPPLSWGVKRPRTRIGGGLPDPGTPMDADLAASSTMIWEQDAAPFVGEEDDFPAFNEEEMAWLMELFPQ |
| TAL18H_CFBP6975 |
| MDPIRPRAPSPAHELLAEPQPDRVQPQPTADRGGSPPAGSPLDGLPARRTMSRTRLPSPPAPLPAFSAGSFRDLLRQFDPSLLDTSLFDSMSAFGAPHTEAASGEGDEVQSGLRAADDPQATVQVAVTAARPPRAKPAPRRRAAHTSDASPAGQVDLCTLGYSQQQQEKIKLKARPIVAQHHEALIGHGFTRAHIVALSQHPAALGTVAVKYQAMIAALPEATHEDIVGVGKQWSGARALEALLTVSGELRGPPLQLDTGQLLKIAKRGGVTAVEAVHAWRNALTGVPLNLTPDQVVAIASNIGGKQALETVQLLLPVLCEQHGLTPDQVVAIASNGGGKQALETVQRLLPVLCKDHGLTPAQVVAIANHDGGKQALETVQRLLPVLCKDHGLTPAQVVAIASNGGGKQALETVEQLLPVLCKDHGLTPDQVVAIANHDGGKPALETVQRLLPVLCQELGLTPDQVVAIASNIGGKQALETVQRLLPVLCEQHGLTPDQVVAIASNGGAKQALETVQRLLPVLCQELGLTPAQVVAIASNIGGKQALETVQRLLPVLCEQHGLTPDQVVAIASHDGGKQALETVERLLPVLCQELGMTLAQVVAIASHYGGKQALEAVQRLLPVLCQELGLTPDQVVAIASNNGGKQALETVQRLLPVLCEQHGLTPDQVVAIASNGGKQALETVQRLLPVLCEQHGLTPDQVVAIASHDGGKQALETVERLLPVLCQELGMTLAQVVAIASNGGGKQALETVERLLPVLCQELGMTLAQVVAIASHYGGKQALETVQRLLPVLCQDLGLTPDQVVAIASNNGGKQALETVQRLLPVLCEQHGLTPDQVVAIASHDGGKQALEAVQRLLPVLCQDHGLNPDQVVAIASNGGGRQALESIFAQLSRPDQALAALTNDHLVALACLGGCPALEAVKKGLPHALTLIKRTSRRLPERTSHRVADHAQVARVLGFFQCHSHPAQAFDEAMAQFAMSRHGLLQLFRRVGVTELEACNGTLPPASQRWHRILQASGVRTATPSRASAQTPDQASLDAFADALERGLDALSPIDQAVQAQASSRRKRSRSESSVTRSSAHYAVEVPVREQHAALDSLPPSWGAKRPRTRIGGGLADPGTPMHGDLAASSTVAWEHDAAPFAAAEAGDFPAFNDEEIAWLMELFPQ |

| TAL23A_CFBP7767R |
| --- |
| MDPIRPRTPSPAHELLAGPQPDRVQPQPTADRGGAPPAGSPLDGLPARRTMSRTRLPSPPAPLPAFSAGSFSDLLRQFDPSLLDTSLFDSMSAFGAPHTEAASGEGDEVQSGLRAADDPQATVQVAVTAARPPRAKPVPRRRAAHTSDASPAGQVDLCTLGYSQQQQEKIKLKARSTVAQHHEALIGHGFTRAQIVALSQHPAALGTVAVKYQVMIAALPEATHEDIVGVGKQWSGARALEALLTVSGELRGPPLQLDTGQLLKIAKRGGVTAVEAVHAWRNALTGAPLNLTPDQVVAIASNIGGKQALETVQRLLPVLCEQHGLTPDQVVAIASNGGGKQALETVQRLLPVLCEQHGLTPDQVVAIASNIGGKQALETVQRLLPVLCEQHGLTPEQVVAIASNGGGKQALETVQRLLPVLCQAHGLTPDQVVAIASNIGGKQALETVQRLLPVLCEQHGLTPDQVVAIASNNGGKQALETVQRLLPVLCEQHGLTPDQVVAIASNGGGKQALETVQRLLPVLCEQHGLTREQVVAIASNNGGKQALETVQRLLPVLCQAHGLTPEQVVAIASHDGGKQALETLQRLLPVLCEQHGLTREQVVAIASHDGGKQALETLQRLLPVLCEQHGLTREQVVAIASNNGGKQALETVQRLLPVLCEQHGLTPAQVVAIASNNGGKQALETVQRLLPVLCEQHGLTPEQVVAIASHDGGKQALETVQRLLPVLCEQHGLTPEQVVAIASNIGGKQALETVQRLLPVLCEQHGLTPDQVVAIASHDGGKQALETVQRLLPVLCEQHGLTREQVVAIASNIGGKQALETVQRLLPVLCEQHGLTPEQVVAIASHDGGKQALETVQRLLPVLCEQHGLTPEQVVAIASHDGGKQALETVQRLLPVLCQAHGLTPEQVVAIASHDGGKQALETVQRLLPVLCQAHGLTPEQVVAIASHDGGKQALETVQRLLPVLCEQHGLTREQVVAIASHDGGKQALETVQRLLPVLCQAHGLTPEQVVAIASNGGGKQALETVQRLLPVLCEQHGLTPAQVVAIASNGGGKQALESIFAQLSRPDQALAALTNDHLVALACLGGRPALEAVKKGLPHAPTLIKRTNRRLPERTSHRVADHAQVARVLGFFQCHSHPAQAFDEAMTQFGMSRHGLLQLFRRVGVTELEARSGTLPPAPQRWHRILQASGMKRAEPSGASAQTPDQASLHAFADALERELDAPSPIDRAGQALASSSRKRSRSESSVTGSFAQQAVEVRVPEQHEGLHFPPLSWGVKRPRTRIGGGLPDPGTPMDADLAASSTMIWEQDAAPFVGEEDDFPAFNEEEMAWLMELFPQ |
| TAL18H_CFBP7767R |
| MDPIRPRAPSPAHELLAEPQPDRVQPQPTADRGGSPPAGSPLDGLPARRTMSRTRLPSPPAPLPAFSAGSFRDLLRQFDPSLLDTSLFDSMSAFGAPHTEAASGEGDEVQSGLRAADDPQATVQVAVTAARPPRAKPAPRRRAAHTSDASPAGQVDLCTLGYSQQQQEKIKLKARPIVAQHHEALIGHGFTRAHIVALSQHPAALGTVAVKYQAMIAALPEATHEDIVGVGKQWSGARALEALLTVSGELRGPPLQLDTGQLLKIAKRGGVTAVEAVHAWRNALTGVPLNLTPDQVVAIASNIGGKQALETVQLLLPVLCEQHGLTPDQVVAIASNGGGKQALETVQRLLPVLCKDHGLTPAQVVAIANHDGGKQALETVQRLLPVLCKDHGLTPAQVVAIASNGGGKQALETVEQLLPVLCKDHGLTPDQVVAIANHDGGKPALETVQRLLPVLCQELGLTPDQVVAIASNIGGKQALETVQRLLPVLCQELGLTPDQVVAIASNGGAKQALETVQRLLPVLCQELGLTPAQVVAIASNIGGKQALETVQRLLPVLCEQHGLTPDQVVAIASHDGGKQALETVERLLPVLCQELGMTLAQVVAIASHYGGKQALEAVQRLLPVLCQELGLTPDQVVAIASNNGGKQALETVQRLLPVLCEQHGLTPDQVVAIASNGGKQALETVQRLLPVLCEQHGLTPDQVVAIASHDGGKQALETVERLLPVLCQELGMTLAQVVAIASNGGGKQALETVERLLPVLCQELGMTLAQVVAIASHYGGKQALEAVQRLLPVLCQDLGLTPDQVVAIASNNGGKQALETVQRLLPVLCEQHGLTPDQVVAIASHDGGKQALEAVQRLLPVLCQDHGLNPDQVVAIASNGGGRQALESIFAQLSRPDQALAALTNDHLVALACLGGCPALEAVKKGLPHALTLIKRTSRRLPERTSHRVADHAQVARVLGFFQCHSHPAQAFDEAMAQFAMSRHGLLQLFRRVGVTELEACNGTLPPASQRWHRILQASGVRTATPSRASAQTPDQASLDAFADALERGLDALSPIDQAVQAQASSRRKRSRSESSVTRSSAHYAVEVPVREQHAALDSLPPSWGAKRPRTRIGGGLADPGTPMHGDLAASSTVAWEHDAAPFAAAEAGDFPAFNDEEIAWLMELFPQ |

| TAL23A_CFBP6988R |
| --- |
| MDPIRPRTPSPAHELLAGPQPDRVQPQPTADRGGAPPAGSPLDGLPARRTMSRTRLPSPPAPLPAFSAGSFSDLLRQFDPSLLDTSLFDSMSAFGAPHTEAASGEGDEVQSGLRAADDPQATVQVAVTAARPPRAKPVPRRRAAHTSDASPAGQVDLCTLGYSQQQQEKIKLKARSTVAQHHEALIGHGFTRAQIVALSQHPAALGTVAVKYQVMIAALPEATHEDIVGVGKQWSGARALEALLTVSGELRGPPLQLDTGQLLKIAKRGGVTAVEAVHAWRNALTGAPLNLTPDQVVAIASNIGGKQALETVQRLLPVLCEQHGLTPDQVVAIASNGGGKQALETVQRLLPVLCEQHGLTPDQVVAIASNIGGKQALETVQRLLPVLCQAHGLTPDQVVAIASNGGGKQALETVQRLLPVLCQAHGLTPDQVVAIASNIGGKQALETVQRLLPVLCEQHGLTPDQVVAIASNNGGKQALETVQRLLPVLCEQHGLTPDQVVAIASNGGGKQALETVQRLLPVLCEQHGLTREQVVAIASNNGGKQALETVQRLLPVLCQAHGLTPEQVVAIASHDGGKQALETLQRLLPVLCEQHGLTREQVVAIASHDGGKQALETLQRLLPVLCEQHGLTREQVVAIASNNGGKQALETVQRLLPVLCEQHGLTPAQVVAIASNNGGKQALETVQRLLPVLCEQHGLTPEQVVAIASHDGGKQALETVQRLLPVLCEQHGLTPEQVVAIASNIGGKQALETVQRLLPVLCEQHGLTPDQVVAIASHDGGKQALETVQRLLPVLCEQHGLTREQVVAIASNIGGKQALETVQRLLPVLCEQHGLTPEQVVAIASHDGGKQALETVQRLLPVLCEQHGLTPEQVVAIASHDGGKQALETVQRLLPVLCQAHGLTPEQVVAIASHDGGKQALETVQRLLPVLCQAHGLTPEQVVAIASHDGGKQALETVQRLLPVLCEQHGLTREQVVAIASHDGGKQALETVQRLLPVLCQAHGLTPEQVVAIASNGGGKQALETVQRLLPVLCEQHGLTPAQVVAIASNGGGKQALESIFAQLSRPDQALAALTNDHLVALACLGGRPALEAVKKGLPHAPTLIKRTNRRLPERTSHRVADHAQVARVLGFFQCHSHPAQAFDEAMTQFGMSRHGLLQLFRRVGVTELEARSGTLPPAPQRWHRILQASGMKRAEPSGASAQTPDQASLHAFADALERELDAPSPIDRAGQALASSSRKRSRSESSVTGSFAQQAVEVRVPEQHEGLHFPPLSWGVKRPRTRIGGGLPDPGTPMDADLAASSTMIWEQDAAPFVGEEDDFPAFNEEEMAWLMELFPQ |
| TAL20F_CFBP6988R |
| MDPIRPRTPSPAHELLAEPQPDRVQPQPTADRGGAPPAGSPLDGLPARRTMSRTRLPSPPTPLPAFSAGSFSDLLRQFDPSLLDTSLFDSMPAFGAHTEAAPGEADEVQSGLRAVDDPHPTVQVAVTAARPPRAKPAPRRRAAHPPDASPAAQVDLCTLGYSQQQQEKIKPKVRSTVAQHHEALVGHGFTHAHIVALSQHPAALGTVAVKYQAMIAALPEATHEDIVGVGKQWSGARALEALLTVSGELRGLPLQLDTGQLLKIAKRGGVTAVEAVHAWRNALTGAPLNLTPDQVVAIASNGGKQALETVQRLLPVLCEQHGLTPDQVVAIASHDGGKQALETVQRLLPVLCEQHGLTPDQVVAIASHDGGKQALETVQRLLPVLCEQHGLTPDQVVAIASNGGGTQALETVQRLLPVLCEQHGLTPDQVVAIASNGGGKQALETVQRLLPVLCQAHGLTPAQVVAIASNIGGKQALETVQRLLPVLCEQHGLTPDQVVAIASNGGGRQALETVQRLLPVLCEQHGLTPDQVVAIASNIGGKQALETVQRLLPVLCQAHGLTPDQVVAIASNIGGKQALETVQRLLPVLCEQHGLTPDQVVAIASNNGGRQALETVHRLLPVLCQAHGLTPDQVVAIASNIGGKQALETVQRLLPVLCEQHGLTPDQVVAIASHDGGRQALETVHRLLPVLCQAHGLTPDQVVAIASHDGGKQALETVQRLLPVLCEQHGLTPDQVVAIASHDGGRQALETVHRLLPVLCQAHGLTPDQVVAIASHDGGKQALETVQRLLPVLCEQHGLTPDQVVAIASHDGGRQALETVQRLLPVLCQAHGLTPDQVVAIASNIGGKQALETVQRLLPVLCEQHGLTPDQVVAIASHDGGRQALETVQRLLPVLCQAHGLTPDQVVTIASNKGGKQALETVHRLLPVLCEQHGLTPDQVVAIASNGGGRQALESIFAQLSRPDQALAALTNDHLVALACLGGRPALEAVKKGLPQAPTLIKRTNRRLPERTSHRVADHAQVARVLGFFQCHSRPAQAFDDAMTQFEMSRHGLLQLFRRAGVTELEARSGALPPASQRWHRILQASGMKRAEPSRASAQTPDQASLHAFADALERELDAPSPIDRAGQALASSSRKRSRSESSVTGSFAQQAVEVRVPEQRDALHLPPLSWGVKRPRTRIGGGLPDPGTPMDADLAASSTMIWEQDAAPFAGAADDFTAFNEEEMAWLMELFHQ |
| TAL18G_CFBP6988R |
| MDPIRPRTPSPAHELLAEPQPDRVQPQPTADRGGAPPAGSPLDGLPARRTMSRTRLPSPPTPLPAFSAGSFSDLLRQFDPSLLDTSLFDSMPAFGAHTEAAPGEADEVQSGLRAVDDPHPTVQVAVTAARPPRAKPAPRRRAAHPPDASPAAQVDLCTLGYSQQQQEKIKPKVRSTVAQHHEALVGHGFTHAHIVALSQHPAALGTVAVKYQAMIAALPEATHEDIVGVGKQWSGARALEALLTVSGELRGLPLQLDTGQLLKIAKRGGVTAVEAVHAWRNALTGAPLNLTPDQVVAIASNGGKQALETVQRLLPVLCEQHGLTPDQVVAIASHDGGKQALETVQRLLPVLCEQHGLTPDQVVAIASHDGGKQALETVQRLLPVLCEQHGLTPDQVVAIASNGGGTQALETVQRLLPVLCEQHGLTPDQVVAIASNGGGKQALETVQRLLPVLCQAHGLTPAQVVAIASNIGGKQALETVQRLLPVLCEQHGLTPDQVVAIASNGGGRQALETVQRLLPVLCEQHGLTPDQVVAIASNIGGKQALETVQRLLPVLCQAHGLTPDQVVAIASNIGGKQALETVQRLLPVLCEQHGLTPDQVVAIASNNGGRQALETVHRLLPVLCQAHGLTPDQVVAIASNIGGKQALETVQRLLPVLCEQHGLTPDQVVAIASHDGGRQALETVHRLLPVLCQAHGLTPDQVVAIASHDGGKQALETVQRLLPVLCEQHGLTPDQVVAIASHDGGRQALETVQRLLPVLCQAHGLTPDQVVAIASNIGGKQALETVQRLLPVLCEQHGLTPDQVVAIASHDGGRQALETVQRLLPVLCQAHGLTPDQVVTIASNKGGKQALETVHRLLPVLCEQHGLTPDQVVAIASNGGGRQALESIFAQLSRPDQALAALTNDHLVALACLGGRPALEAVKKGLPQAPTLIKRTNRRLPERTSHRVADHAQVARVLGFFQCHSRPAQAFDDAMTQFEMSRHGLLQLFRRAGVTELEARSGALPPASQRWHRILQASGMKRAEPSRASAQTPNQASLHAFADALERELDAPSPIDRAGQALASSSRKRSRSESSVTGSFAQQAVEVRVPEQRDALHLPPLSWGVKRPRTRIGGGLPDPGTPMDADLAASSTMIWEQDAAPFAGAADDFTAFNEEEMAWLMELFHQ |
| TAL23A_CFBP6989 |
| MDPIRPRTPSPAHELLAGPQPDRVQPQPTADRGGAPPAGSPLDGLPARRTMSRTRLPSPPAPLPAFSAGSFSDLLRQFDPSLLDTSLFDSMSAFGAPHTEAASGEGDEVQSGLRAADDPQATVQVAVTAARPPRAKPVPRRRAAHTSDASPAGQVDLCTLGYSQQQQEKIKLKARSTVAQHHEALIGHGFTRAQIVALSQHPAALGTVAVKYQVMIAALPEATHEDIVGVGKQWSGARALEALLTVSGELRGPPLQLDTGQLLKIAKRGGVTAVEAVHAWRNALTGAPLNLTPDQVVAIASNIGGKQALETVQRLLPVLCEQHGLTPDQVVAIASNGGGKQALETVQRLLPVLCEQHGLTPDQVVAIASNIGGKQALETVQRLLPVLCQAHGLTPDQVVAIASNGGGKQALETVQRLLPVLCQAHGLTPDQVVAIASNIGGKQALETVQRLLPVLCEQHGLTPDQVVAIASNNGGKQALETVQRLLPVLCEQHGLTPDQVVAIASNGGGKQALETVQRLLPVLCEQHGLTREQVVAIASNNGGKQALETVQRLLPVLCQAHGLTPEQVVAIASHDGGKQALETLQRLLPVLCEQHGLTREQVVAIASHDGGKQALETLQRLLPVLCEQHGLTREQVVAIASNNGGKQALETVQRLLPVLCEQHGLTPAQVVAIASNNGGKQALETVQRLLPVLCEQHGLTPEQVVAIASHDGGKQALETVQRLLPVLCEQHGLTPEQVVAIASNIGGKQALETVQRLLPVLCEQHGLTPDQVVAIASHDGGKQALETVQRLLPVLCEQHGLTREQVVAIASNIGGKQALETVQRLLPVLCEQHGLTPEQVVAIASHDGGKQALETVQRLLPVLCEQHGLTPEQVVAIASHDGGKQALETVQRLLPVLCQAHGLTPEQVVAIASHDGGKQALETVQRLLPVLCQAHGLTPEQVVAIASHDGGKQALETVQRLLPVLCEQHGLTREQVVAIASHDGGKQALETVQRLLPVLCQAHGLTPEQVVAIASNGGGKQALETVQRLLPVLCEQHGLTPAQVVAIASNGGGKQALESIFAQLSRPDQALAALTNDHLVALACLGGRPALEAVKKGLPHAPTLIKRTNRRLPERTSHRVADHAQVARVLGFFQCHSHPAQAFDEAMTQFGMSRHGLLQLFRRVGVTELEARSGTLPPAPQRWHRILQASGMKRAEPSGASAQTPDQASLHAFADALERELDAPSPIDRAGQALASSSRKRSRSESSVTGSFAQQAVEVRVPEQHEGLHFPPLSWGVKRPRTRIGGGLPDPGTPMDADLAASSTMIWEQDAAPFVGEEDDFPAFNEEEMAWLMELFPQ |
| TAL20F_CFBP6989 |
| MDPIRPRTPSPAHELLAEPQPDRVQPQPTADRGGAPPAGSPLDGLPARRTMSRTRLPSPPTPLPAFSAGSFSDLLRQFDPSLLDTSLFDSMPAFGAHTEAAPGEADEVQSGLRAVDDPHPTVQVAVTAARPPRAKPAPRRRAAHPPDASPAAQVDLCTLGYSQQQQEKIKPKVRSTVAQHHEALVGHGFTHAHIVALSQHPAALGTVAVKYQAMIAALPEATHEDIVGVGKQWSGARALEALLTVSGELRGLPLQLDTGQLLKIAKRGGVTAVEAVHAWRNALTGAPLNLTPDQVVAIASNGGKQALETVQRLLPVLCEQHGLTPDQVVAIASHDGGKQALETVQRLLPVLCEQHGLTPDQVVAIASHDGGKQALETVQRLLPVLCEQHGLTPDQVVAIASNGGGTQALETVQRLLPVLCEQHGLTPDQVVAIASNGGGKQALETVQRLLPVLCQAHGLTPAQVVAIASNIGGKQALETVQRLLPVLCEQHGLTPDQVVAIASNGGGRQALETVQRLLPVLCEQHGLTPDQVVAIASNIGGKQALETVQRLLPVLCQAHGLTPDQVVAIASNIGGKQALETVQRLLPVLCEQHGLTPDQVVAIASNNGGRQALETVHRLLPVLCQAHGLTPDQVVAIASNIGGKQALETVQRLLPVLCEQHGLTPDQVVAIASHDGGRQALETVHRLLPVLCQAHGLTPDQVVAIASHDGGKQALETVQRLLPVLCEQHGLTPDQVVAIASHDGGRQALETVHRLLPVLCQAHGLTPDQVVAIASHDGGKQALETVQRLLPVLCEQHGLTPDQVVAIASHDGGRQALETVQRLLPVLCQAHGLTPDQVVAIASNIGGKQALETVQRLLPVLCEQHGLTPDQVVAIASHDGGRQALETVQRLLPVLCQAHGLTPDQVVTIASNKGGKQALETVHRLLPVLCEQHGLTPDQVVAIASNGGGRQALESIFAQLSRPDQALAALTNDHLVALACLGGRPALEAVKKGLPQAPTLIKRTNRRLPERTSHRVADHAQVARVLGFFQCHSRPAQAFDDAMTQFEMSRHGLLQLFRRAGVTELEARSGALPPASQRWHRILQASGMKRAEPSRASAQTPDQASLHAFADALERELDAPSPIDRAGQALASSSRKRSRSESSVTGSFAQQAVEVRVPEQRDALHLPPLSWGVKRPRTRIGGGLPDPGTPMDADLAASSTMIWEQDAAPFAGAADDFTAFNEEEMAWLMELFHQ |
| TAL18G_CFBP6989 |
| MDPIRPRTPSPAHELLAEPQPDRVQPQPTADRGGAPPAGSPLDGLPARRTMSRTRLPSPPTPLPAFSAGSFSDLLRQFDPSLLDTSLFDSMPAFGAHTEAAPGEADEVQSGLRAVDDPHPTVQVAVTAARPPRAKPAPRRRAAHPPDASPAAQVDLCTLGYSQQQQEKIKPKVRSTVAQHHEALVGHGFTHAHIVALSQHPAALGTVAVKYQAMIAALPEATHEDIVGVGKQWSGARALEALLTVSGELRGLPLQLDTGQLLKIAKRGGVTAVEAVHAWRNALTGAPLNLTPDQVVAIASNGGKQALETVQRLLPVLCEQHGLTPDQVVAIASHDGGKQALETVQRLLPVLCEQHGLTPDQVVAIASHDGGKQALETVQRLLPVLCEQHGLTPDQVVAIASNGGGTQALETVQRLLPVLCEQHGLTPDQVVAIASNGGGKQALETVQRLLPVLCQAHGLTPAQVVAIASNIGGKQALETVQRLLPVLCEQHGLTPDQVVAIASNGGGRQALETVQRLLPVLCEQHGLTPDQVVAIASNIGGKQALETVQRLLPVLCQAHGLTPDQVVAIASNIGGKQALETVQRLLPVLCEQHGLTPDQVVAIASNNGGRQALETVHRLLPVLCQAHGLTPDQVVAIASNIGGKQALETVQRLLPVLCEQHGLTPDQVVAIASHDGGRQALETVHRLLPVLCQAHGLTPDQVVAIASHDGGKQALETVQRLLPVLCEQHGLTPDQVVAIASHDGGRQALETVQRLLPVLCQAHGLTPDQVVAIASNIGGKQALETVQRLLPVLCEQHGLTPDQVVAIASHDGGRQALETVQRLLPVLCQAHGLTPDQVVTIASNKGGKQALETVHRLLPVLCEQHGLTPDQVVAIASNGGGRQALESIFAQLSRPDQALAALTNDHLVALACLGGRPALEAVKKGLPQAPTLIKRTNRRLPERTSHRVADHAQVARVLGFFQCHSRPAQAFDDAMTQFEMSRHGLLQLFRRAGVTELEARSGALPPASQRWHRILQASGMKRAEPSRASAQTPNQASLHAFADALERELDAPSPIDRAGQALASSSRKRSRSESSVTGSFAQQAVEVRVPEQRDALHLPPLSWGVKRPRTRIGGGLPDPGTPMDADLAASSTMIWEQDAAPFAGAADDFTAFNEEEMAWLMELFHQ |
| TAL23A_CFBP6990 |
| MDPIRPRTPSPAHELLAGPQPDRVQPQPTADRGGAPPAGSPLDGLPARRTMSRTRLPSPPAPLPAFSAGSFSDLLRQFDPSLLDTSLFDSMSAFGAPHTEAASGEGDEVQSGLRAADDPQATVQVAVTAARPPRAKPVPRRRAAHTSDASPAGQVDLCTLGYSQQQQEKIKLKARSTVAQHHEALIGHGFTRAQIVALSQHPAALGTVAVKYQVMIAALPEATHEDIVGVGKQWSGARALEALLTVSGELRGPPLQLDTGQLLKIAKRGGVTAVEAVHAWRNALTGAPLNLTPDQVVAIASNIGGKQALETVQRLLPVLCEQHGLTPDQVVAIASNGGGKQALETVQRLLPVLCEQHGLTPDQVVAIASNIGGKQALETVQRLLPVLCQAHGLTPDQVVAIASNGGGKQALETVQRLLPVLCQAHGLTPDQVVAIASNIGGKQALETVQRLLPVLCEQHGLTPDQVVAIASNNGGKQALETVQRLLPVLCEQHGLTPDQVVAIASNGGGKQALETVQRLLPVLCEQHGLTREQVVAIASNNGGKQALETVQRLLPVLCQAHGLTPEQVVAIASHDGGKQALETLQRLLPVLCEQHGLTREQVVAIASHDGGKQALETLQRLLPVLCEQHGLTREQVVAIASNNGGKQALETVQRLLPVLCEQHGLTPAQVVAIASNNGGKQALETVQRLLPVLCEQHGLTPEQVVAIASHDGGKQALETVQRLLPVLCEQHGLTPEQVVAIASNIGGKQALETVQRLLPVLCEQHGLTPDQVVAIASHDGGKQALETVQRLLPVLCEQHGLTREQVVAIASNIGGKQALETVQRLLPVLCEQHGLTPEQVVAIASHDGGKQALETVQRLLPVLCEQHGLTPEQVVAIASHDGGKQALETVQRLLPVLCQAHGLTPEQVVAIASHDGGKQALETVQRLLPVLCQAHGLTPEQVVAIASHDGGKQALETVQRLLPVLCEQHGLTREQVVAIASHDGGKQALETVQRLLPVLCQAHGLTPEQVVAIASNGGGKQALETVQRLLPVLCEQHGLTPAQVVAIASNGGGKQALESIFAQLSRPDQALAALTNDHLVALACLGGRPALEAVKKGLPHAPTLIKRTNRRLPERTSHRVADHAQVARVLGFFQCHSHPAQAFDEAMTQFGMSRHGLLQLFRRVGVTELEARSGTLPPAPQRWHRILQASGMKRAEPSGASAQTPDQASLHAFADALERELDAPSPIDRAGQALASSSRKRSRSESSVTGSFAQQAVEVRVPEQHEGLHFPPLSWGVKRPRTRIGGGLPDPGTPMDADLAASSTMIWEQDAAPFVGEEDDFPAFNEEEMAWLMELFPQ |
| TAL20F_CFBP6990 |
| MDPIRPRTPSPAHELLAEPQPDRVQPQPTADRGGAPPAGSPLDGLPARRTMSRTRLPSPPTPLPAFSAGSFSDLLRQFDPSLLDTSLFDSMPAFGAHTEAAPGEADEVQSGLRAVDDPHPTVQVAVTAARPPRAKPAPRRRAAHPPDASPAAQVDLCTLGYSQQQQEKIKPKVRSTVAQHHEALVGHGFTHAHIVALSQHPAALGTVAVKYQAMIAALPEATHEDIVGVGKQWSGARALEALLTVSGELRGLPLQLDTGQLLKIAKRGGVTAVEAVHAWRNALTGAPLNLTPDQVVAIASNGGKQALETVQRLLPVLCEQHGLTPDQVVAIASHDGGKQALETVQRLLPVLCEQHGLTPDQVVAIASHDGGKQALETVQRLLPVLCEQHGLTPDQVVAIASNGGGTQALETVQRLLPVLCEQHGLTPDQVVAIASNGGGKQALETVQRLLPVLCQAHGLTPAQVVAIASNIGGKQALETVQRLLPVLCEQHGLTPDQVVAIASNGGGRQALETVQRLLPVLCEQHGLTPDQVVAIASNIGGKQALETVQRLLPVLCQAHGLTPDQVVAIASNIGGKQALETVQRLLPVLCEQHGLTPDQVVAIASNNGGRQALETVHRLLPVLCQAHGLTPDQVVAIASNIGGKQALETVQRLLPVLCEQHGLTPDQVVAIASHDGGRQALETVHRLLPVLCQAHGLTPDQVVAIASHDGGKQALETVQRLLPVLCEQHGLTPDQVVAIASHDGGRQALETVHRLLPVLCQAHGLTPDQVVAIASHDGGKQALETVQRLLPVLCEQHGLTPDQVVAIASHDGGRQALETVQRLLPVLCQAHGLTPDQVVAIASNIGGKQALETVQRLLPVLCEQHGLTPDQVVAIASHDGGRQALETVQRLLPVLCQAHGLTPDQVVTIASNKGGKQALETVHRLLPVLCEQHGLTPDQVVAIASNGGGRQALESIFAQLSRPDQALAALTNDHLVALACLGGRPALEAVKKGLPQAPTLIKRTNRRLPERTSHRVADHAQVARVLGFFQCHSRPAQAFDDAMTQFEMSRHGLLQLFRRAGVTELEARSGALPPASQRWHRILQASGMKRAEPSRASAQTPDQASLHAFADALERELDAPSPIDRAGQALASSSRKRSRSESSVTGSFAQQAVEVRVPEQRDALHLPPLSWGVKRPRTRIGGGLPDPGTPMDADLAASSTMIWEQDAAPFAGAADDFTAFNEEEMAWLMELFHQ |
| TAL18G_CFBP6990 |
| MDPIRPRTPSPAHELLAEPQPDRVQPQPTADRGGAPPAGSPLDGLPARRTMSRTRLPSPPTPLPAFSAGSFSDLLRQFDPSLLDTSLFDSMPAFGAHTEAAPGEADEVQSGLRAVDDPHPTVQVAVTAARPPRAKPAPRRRAAHPPDASPAAQVDLCTLGYSQQQQEKIKPKVRSTVAQHHEALVGHGFTHAHIVALSQHPAALGTVAVKYQAMIAALPEATHEDIVGVGKQWSGARALEALLTVSGELRGLPLQLDTGQLLKIAKRGGVTAVEAVHAWRNALTGAPLNLTPDQVVAIASNGGKQALETVQRLLPVLCEQHGLTPDQVVAIASHDGGKQALETVQRLLPVLCEQHGLTPDQVVAIASHDGGKQALETVQRLLPVLCEQHGLTPDQVVAIASNGGGTQALETVQRLLPVLCEQHGLTPDQVVAIASNGGGKQALETVQRLLPVLCQAHGLTPAQVVAIASNIGGKQALETVQRLLPVLCEQHGLTPDQVVAIASNGGGRQALETVQRLLPVLCEQHGLTPDQVVAIASNIGGKQALETVQRLLPVLCQAHGLTPDQVVAIASNIGGKQALETVQRLLPVLCEQHGLTPDQVVAIASNNGGRQALETVHRLLPVLCQAHGLTPDQVVAIASNIGGKQALETVQRLLPVLCEQHGLTPDQVVAIASHDGGRQALETVHRLLPVLCQAHGLTPDQVVAIASHDGGKQALETVQRLLPVLCEQHGLTPDQVVAIASHDGGRQALETVQRLLPVLCQAHGLTPDQVVAIASNIGGKQALETVQRLLPVLCEQHGLTPDQVVAIASHDGGRQALETVQRLLPVLCQAHGLTPDQVVTIASNKGGKQALETVHRLLPVLCEQHGLTPDQVVAIASNGGGRQALESIFAQLSRPDQALAALTNDHLVALACLGGRPALEAVKKGLPQAPTLIKRTNRRLPERTSHRVADHAQVARVLGFFQCHSRPAQAFDDAMTQFEMSRHGLLQLFRRAGVTELEARSGALPPASQRWHRILQASGMKRAEPSRASAQTPNQASLHAFADALERELDAPSPIDRAGQALASSSRKRSRSESSVTGSFAQQAVEVRVPEQRDALHLPPLSWGVKRPRTRIGGGLPDPGTPMDADLAASSTMIWEQDAAPFAGAADDFTAFNEEEMAWLMELFHQ |
| TAL22C_CFBP6991 |
| MDPIRPRTPSPAHELLAGPQPDRVQPQPTADRGGAPPAGSPLDGLPARRTMSRTRLPSPPAPLPAFSAGSFSDLLRQFDPSLLDTSLFDSMSAFGAPHTEAASGEGDEVQSGLRAADDPQATVQVAVTAARPPRAKPVPRRRAAHTSDASPAGQVDLCTLGYSQQQQEKIKLKARSTVAQHHEALIGHGFTRAQIVALSQHPAALGTVAVKYQVMIAALPEATHEDIVGVGKQWSGARALEALLTVSGELRGPPLQLDTGQLLKIAKRGGVTAVEAVHAWRNALTGAPLNLTPDQVVAIASNGGGKQALETVQRLLPVLCEQHGLTPDQVVAIASNIGGKQALETVQRLLPVLCQAHGLTPDQVVAIASNGGGKQALETVQRLLPVLCQAHGLTPDQVVAIASNIGGKQALETVQRLLPVLCEQHGLTPDQVVAIASNNGGKQALETVQRLLPVLCEQHGLTPDQVVAIASNGGGKQALETVQRLLPVLCEQHGLTREQVVAIASNNGGKQALETVQRLLPVLCQAHGLTPEQVVAIASHDGGKQALETLQRLLPVLCEQHGLTREQVVAIASHDGGKQALETLQRLLPVLCEQHGLTREQVVAIASNNGGKQALETVQRLLPVLCEQHGLTPAQVVAIASNNGGKQALETVQRLLPVLCEQHGLTPEQVVAIASHDGGKQALETVQRLLPVLCEQHGLTPEQVVAIASNIGGKQALETVQRLLPVLCEQHGLTPDQVVAIASHDGGKQALETVQRLLPVLCEQHGLTREQVVAIASNIGGKQALETVQRLLPVLCEQHGLTPEQVVAIASHDGGKQALETVQRLLPVLCEQHGLTPEQVVAIASHDGGKQALETVQRLLPVLCQAHGLTPEQVVAIASHDGGKQALETVQRLLPVLCQAHGLTPEQVVAIASHDGGKQALETVQRLLPVLCEQHGLTREQVVAIASHDGGKQALETVQRLLPVLCQAHGLTPEQVVAIASNGGGKQALETVQRLLPVLCEQHGLTPAQVVAIASNGGGKQALESIFAQLSRPDQALAALTNDHLVALACLGGRPALEAVKKGLPHAPTLIKRTNRRLPERTSHRVADHAQVARVLGFFQCHSHPAQAFDEAMTQFGMSRHGLLQLFRRVGVTELEARSGTLPPAPQRWHRILQASGMKRAEPSGASAQTPDQASLHAFADALERELDAPSPIDRAGQALASSSRKRSRSESSVTGSFAQQAVEVRVPEQHEGLHFPPLSWGVKRPRTRIGGGLPDPGTPMDADLAASSTMIWEQDAAPFVGEEDDFPAFNEEEMAWLMELFPQ |
| TAL20F_CFBP6991 |
| MDPIRPRTPSPAHELLAEPQPDRVQPQPTADRGGAPPAGSPLDGLPARRTMSRTRLPSPPTPLPAFSAGSFSDLLRQFDPSLLDTSLFDSMPAFGAHTEAAPGEADEVQSGLRAVDDPHPTVQVAVTAARPPRAKPAPRRRAAHPPDASPAAQVDLCTLGYSQQQQEKIKPKVRSTVAQHHEALVGHGFTHAHIVALSQHPAALGTVAVKYQAMIAALPEATHEDIVGVGKQWSGARALEALLTVSGELRGLPLQLDTGQLLKIAKRGGVTAVEAVHAWRNALTGAPLNLTPDQVVAIASNGGKQALETVQRLLPVLCEQHGLTPDQVVAIASHDGGKQALETVQRLLPVLCEQHGLTPDQVVAIASHDGGKQALETVQRLLPVLCEQHGLTPDQVVAIASNGGGTQALETVQRLLPVLCEQHGLTPDQVVAIASNGGGKQALETVQRLLPVLCQAHGLTPAQVVAIASNIGGKQALETVQRLLPVLCEQHGLTPDQVVAIASNGGGRQALETVQRLLPVLCEQHGLTPDQVVAIASNIGGKQALETVQRLLPVLCQAHGLTPDQVVAIASNIGGKQALETVQRLLPVLCEQHGLTPDQVVAIASNNGGRQALETVHRLLPVLCQAHGLTPDQVVAIASNIGGKQALETVQRLLPVLCEQHGLTPDQVVAIASHDGGRQALETVHRLLPVLCQAHGLTPDQVVAIASHDGGKQALETVQRLLPVLCEQHGLTPDQVVAIASHDGGRQALETVHRLLPVLCQAHGLTPDQVVAIASHDGGKQALETVQRLLPVLCEQHGLTPDQVVAIASHDGGRQALETVQRLLPVLCQAHGLTPDQVVAIASNIGGKQALETVQRLLPVLCEQHGLTPDQVVAIASHDGGRQALETVQRLLPVLCQAHGLTPDQVVTIASNKGGKQALETVHRLLPVLCEQHGLTPDQVVAIASNGGGRQALESIFAQLSRPDQALAALTNDHLVALACLGGRPALEAVKKGLPQAPTLIKRTNRRLPERTSHRVADHAQVARVLGFFQCHSRPAQAFDDAMTQFEMSRHGLLQLFRRAGVTELEARSGALPPASQRWHRILQASGMKRAEPSRASAQTPDQASLHAFADALERELDAPSPIDRAGQALASSSRKRSRSESSVTGSFAQQAVEVRVPEQRDALHLPPLSWGVKRPRTRIGGGLPDPGTPMDADLAASSTMIWEQDAAPFAGAADDFTAFNEEEMAWLMELFHQ |
| TAL18G_CFBP6991 |
| MDPIRPRTPSPAHELLAEPQPDRVQPQPTADRGGAPPAGSPLDGLPARRTMSRTRLPSPPTPLPAFSAGSFSDLLRQFDPSLLDTSLFDSMPAFGAHTEAAPGEADEVQSGLRAVDDPHPTVQVAVTAARPPRAKPAPRRRAAHPPDASPAAQVDLCTLGYSQQQQEKIKPKVRSTVAQHHEALVGHGFTHAHIVALSQHPAALGTVAVKYQAMIAALPEATHEDIVGVGKQWSGARALEALLTVSGELRGLPLQLDTGQLLKIAKRGGVTAVEAVHAWRNALTGAPLNLTPDQVVAIASNGGKQALETVQRLLPVLCEQHGLTPDQVVAIASHDGGKQALETVQRLLPVLCEQHGLTPDQVVAIASHDGGKQALETVQRLLPVLCEQHGLTPDQVVAIASNGGGTQALETVQRLLPVLCEQHGLTPDQVVAIASNGGGKQALETVQRLLPVLCQAHGLTPAQVVAIASNIGGKQALETVQRLLPVLCEQHGLTPDQVVAIASNGGGRQALETVQRLLPVLCEQHGLTPDQVVAIASNIGGKQALETVQRLLPVLCQAHGLTPDQVVAIASNIGGKQALETVQRLLPVLCEQHGLTPDQVVAIASNNGGRQALETVHRLLPVLCQAHGLTPDQVVAIASNIGGKQALETVQRLLPVLCEQHGLTPDQVVAIASHDGGRQALETVHRLLPVLCQAHGLTPDQVVAIASHDGGKQALETVQRLLPVLCEQHGLTPDQVVAIASHDGGRQALETVQRLLPVLCQAHGLTPDQVVAIASNIGGKQALETVQRLLPVLCEQHGLTPDQVVAIASHDGGRQALETVQRLLPVLCQAHGLTPDQVVTIASNKGGKQALETVHRLLPVLCEQHGLTPDQVVAIASNGGGRQALESIFAQLSRPDQALAALTNDHLVALACLGGRPALEAVKKGLPQAPTLIKRTNRRLPERTSHRVADHAQVARVLGFFQCHSRPAQAFDDAMTQFEMSRHGLLQLFRRAGVTELEARSGALPPASQRWHRILQASGMKRAEPSRASAQTPNQASLHAFADALERELDAPSPIDRAGQALASSSRKRSRSESSVTGSFAQQAVEVRVPEQRDALHLPPLSWGVKRPRTRIGGGLPDPGTPMDADLAASSTMIWEQDAAPFAGAADDFTAFNEEEMAWLMELFHQ |
| TAL23A_CFBP6992 |
| MDPIRPRTPSPAHELLAGPQPDRVQPQPTADRGGAPPAGSPLDGLPARRTMSRTRLPSPPAPLPAFSAGSFSDLLRQFDPSLLDTSLFDSMSAFGAPHTEAASGEGDEVQSGLRAADDPQATVQVAVTAARPPRAKPVPRRRAAHTSDASPAGQVDLCTLGYSQQQQEKIKLKARSTVAQHHEALIGHGFTRAQIVALSQHPAALGTVAVKYQVMIAALPEATHEDIVGVGKQWSGARALEALLTVSGELRGPPLQLDTGQLLKIAKRGGVTAVEAVHAWRNALTGAPLNLTPDQVVAIASNIGGKQALETVQRLLPVLCEQHGLTPDQVVAIASNGGGKQALETVQRLLPVLCEQHGLTPDQVVAIASNIGGKQALETVQRLLPVLCQAHGLTPDQVVAIASNGGGKQALETVQRLLPVLCQAHGLTPDQVVAIASNIGGKQALETVQRLLPVLCEQHGLTPDQVVAIASNNGGKQALETVQRLLPVLCEQHGLTPDQVVAIASNGGGKQALETVQRLLPVLCEQHGLTREQVVAIASNNGGKQALETVQRLLPVLCQAHGLTPEQVVAIASHDGGKQALETLQRLLPVLCEQHGLTREQVVAIASHDGGKQALETLQRLLPVLCEQHGLTREQVVAIASNNGGKQALETVQRLLPVLCEQHGLTPAQVVAIASNNGGKQALETVQRLLPVLCEQHGLTPEQVVAIASHDGGKQALETVQRLLPVLCEQHGLTPEQVVAIASNIGGKQALETVQRLLPVLCEQHGLTPDQVVAIASHDGGKQALETVQRLLPVLCEQHGLTREQVVAIASNIGGKQALETVQRLLPVLCEQHGLTPEQVVAIASHDGGKQALETVQRLLPVLCEQHGLTPEQVVAIASHDGGKQALETVQRLLPVLCQAHGLTPEQVVAIASHDGGKQALETVQRLLPVLCQAHGLTPEQVVAIASHDGGKQALETVQRLLPVLCEQHGLTREQVVAIASHDGGKQALETVQRLLPVLCQAHGLTPEQVVAIASNGGGKQALETVQRLLPVLCEQHGLTPAQVVAIASNGGGKQALESIFAQLSRPDQALAALTNDHLVALACLGGRPALEAVKKGLPHAPTLIKRTNRRLPERTSHRVADHAQVARVLGFFQCHSHPAQAFDEAMTQFGMSRHGLLQLFRRVGVTELEARSGTLPPAPQRWHRILQASGMKRAEPSGASAQTPDQASLHAFADALERELDAPSPIDRAGQALASSSRKRSRSESSVTGSFAQQAVEVRVPEQHEGLHFPPLSWGVKRPRTRIGGGLPDPGTPMDADLAASSTMIWEQDAAPFVGEEDDFPAFNEEEMAWLMELFPQ |

| TAL23A_CFBP6994R |
| --- |
| MDPIRPRTPSPAHELLAGPQPDRVQPQPTADRGGAPPAGSPLDGLPARRTMSRTRLPSPPAPLPAFSAGSFSDLLRQFDPSLLDTSLFDSMSAFGAPHTEAASGEGDEVQSGLRAADDPQATVQVAVTAARPPRAKPVPRRRAAHTSDASPAGQVDLCTLGYSQQQQEKIKLKARSTVAQHHEALIGHGFTRAQIVALSQHPAALGTVAVKYQVMIAALPEATHEDIVGVGKQWSGARALEALLTVSGELRGPPLQLDTGQLLKIAKRGGVTAVEAVHAWRNALTGAPLNLTPDQVVAIASNIGGKQALETVQRLLPVLCEQHGLTPDQVVAIASNGGGKQALETVQRLLPVLCEQHGLTPDQVVAIASNIGGKQALETVQRLLPVLCQAHGLTPDQVVAIASNGGGKQALETVQRLLPVLCQAHGLTPDQVVAIASNIGGKQALETVQRLLPVLCEQHGLTPDQVVAIASNNGGKQALETVQRLLPVLCEQHGLTPDQVVAIASNGGGKQALETVQRLLPVLCEQHGLTREQVVAIASNNGGKQALETVQRLLPVLCQAHGLTPEQVVAIASHDGGKQALETLQRLLPVLCEQHGLTREQVVAIASHDGGKQALETLQRLLPVLCEQHGLTREQVVAIASNNGGKQALETVQRLLPVLCEQHGLTPAQVVAIASNNGGKQALETVQRLLPVLCEQHGLTPEQVVAIASHDGGKQALETVQRLLPVLCEQHGLTPEQVVAIASNIGGKQALETVQRLLPVLCEQHGLTPDQVVAIASHDGGKQALETVQRLLPVLCEQHGLTREQVVAIASNIGGKQALETVQRLLPVLCEQHGLTPEQVVAIASHDGGKQALETVQRLLPVLCEQHGLTPEQVVAIASHDGGKQALETVQRLLPVLCQAHGLTPEQVVAIASHDGGKQALETVQRLLPVLCQAHGLTPEQVVAIASHDGGKQALETVQRLLPVLCEQHGLTREQVVAIASHDGGKQALETVQRLLPVLCQAHGLTPEQVVAIASNGGGKQALETVQRLLPVLCEQHGLTPAQVVAIASNGGGKQALESIFAQLSRPDQALAALTNDHLVALACLGGRPALEAVKKGLPHAPTLIKRTNRRLPERTSHRVADHAQVARVLGFFQCHSHPAQAFDEAMTQFGMSRHGLLQLFRRVGVTELEARSGTLPPAPQRWHRILQASGMKRAEPSGASAQTPDQASLHAFADALERELDAPSPIDRAGQALASSSRKRSRSESSVTGSFAQQAVEVRVPEQHEGLHFPPLSWGVKRPRTRIGGGLPDPGTPMDADLAASSTMIWEQDAAPFVGEEDDFPAFNEEEMAWLMELFPQ |

| TAL23A_CFBP6996R |
| --- |
| MDPIRPRTPSPAHELLAGPQPDRVQPQPTADRGGAPPAGSPLDGLPARRTMSRTRLPSPPAPLPAFSAGSFSDLLRQFDPSLLDTSLFDSMSAFGAPHTEAASGEGDEVQSGLRAADDPQATVQVAVTAARPPRAKPVPRRRAAHTSDASPAGQVDLCTLGYSQQQQEKIKLKARSTVAQHHEALIGHGFTRAQIVALSQHPAALGTVAVKYQVMIAALPEATHEDIVGVGKQWSGARALEALLTVSGELRGPPLQLDTGQLLKIAKRGGVTAVEAVHAWRNALTGAPLNLTPDQVVAIASNIGGKQALETVQRLLPVLCEQHGLTPDQVVAIASNGGGKQALETVQRLLPVLCEQHGLTPDQVVAIASNIGGKQALETVQRLLPVLCQAHGLTPDQVVAIASNGGGKQALETVQRLLPVLCQAHGLTPDQVVAIASNIGGKQALETVQRLLPVLCEQHGLTPDQVVAIASNNGGKQALETVQRLLPVLCEQHGLTPDQVVAIASNGGGKQALETVQRLLPVLCEQHGLTREQVVAIASNNGGKQALETVQRLLPVLCQAHGLTPEQVVAIASHDGGKQALETLQRLLPVLCEQHGLTREQVVAIASHDGGKQALETLQRLLPVLCEQHGLTREQVVAIASNNGGKQALETVQRLLPVLCEQHGLTPAQVVAIASNNGGKQALETVQRLLPVLCEQHGLTPEQVVAIASHDGGKQALETVQRLLPVLCEQHGLTPEQVVAIASNIGGKQALETVQRLLPVLCEQHGLTPDQVVAIASHDGGKQALETVQRLLPVLCEQHGLTREQVVAIASNIGGKQALETVQRLLPVLCEQHGLTPEQVVAIASHDGGKQALETVQRLLPVLCEQHGLTPEQVVAIASHDGGKQALETVQRLLPVLCQAHGLTPEQVVAIASHDGGKQALETVQRLLPVLCQAHGLTPEQVVAIASHDGGKQALETVQRLLPVLCEQHGLTREQVVAIASHDGGKQALETVQRLLPVLCQAHGLTPEQVVAIASNGGGKQALETVQRLLPVLCEQHGLTPAQVVAIASNGGGKQALESIFAQLSRPDQALAALTNDHLVALACLGGRPALEAVKKGLPHAPTLIKRTNRRLPERTSHRVADHAQVARVLGFFQCHSHPAQAFDEAMTQFGMSRHGLLQLFRRVGVTELEARSGTLPPAPQRWHRILQASGMKRAEPSGASAQTPDQASLHAFADALERELDAPSPIDRAGQALASSSRKRSRSESSVTGSFAQQAVEVRVPEQHEGLHFPPLSWGVKRPRTRIGGGLPDPGTPMDADLAASSTMIWEQDAAPFVGEEDDFPAFNEEEMAWLMELFPQ |

| TAL19E_CFBP412 |
| --- |
| MDPIRPRTPSPAHELLAGPQPDRVQPQPTADRGGAPPAGSPLDGLPARRTMSRTRLPSPPAPLPAFSAGSFSDLLRQFDPSLLDTSLFDSMSAFGAPHTEAASGEGDEVQSGLRAADDPQATVQVAVTAARPPRAKPVPRRRAAHTSDASPAGQVDLCTLGYSQQQQEKIKLKARSTVAQHHEALIGHGFTRAQIVALSQHPAALGTVAVKYQVMIAALPEATHEDIVGVGKQWSGARALEALLTVSGELRGPPLQLDTGQLLKIAKRGGVTAVEAVHAWRNALTGAPLNLTPDQVVAIASNIGGKQALETVQRLLPVLCEQHGLTPDQVVAIASNGGGKQALETVQRLLPVLCEQHGLTPDQVVAIASNIGGKQALETVQRLLPVLCEQHGLTPEQVVAIASNGGGKQALETVQRLLPVLCQAHGLTPDQVVAIASNIGGKQALETVQRLLPVLCEQHGLTPDQVVAIASNNGGKQALETVQRLLPVLCEQHGLTPDQVVAIASNGGGKQALETVQRLLPVLCEQHGLTREQVVAIASNNGGKQALETVQRLLPVLCQAHGLTPEQVVAIASHDGGKQALETVQRLLPVLCEQHGLTREQVVAIASHDGGKQALETLQRLLPVLCEQHGLTREQVVAIASNNGGKQALETVQRLLPVLCEQHGLTPDQVVAIASNNGGKQALETVQRLLPVLCEQHGLTPDQVVAIASHDGGKQALETLQRLLPVLCEQHGLTREQVVAIASNIGGKQALETVQRLLPVLCEQHGLTPEQVVAIASHDGGKQALETVQRLLPVLCEQHGLTPEQVVAIASHDGGKQALETVQRLLPVLCQAHGLTPEQVVAIASHDGGKQALETVQRLLPVLCQAHGLTPEQVVAIASHDGGKQALETVQRLLPVLCEQHGLTPAQVVAIASNGGGKQALESIFAQLSRPDQALAALTNDHLVALACLGGRPALEAVKKGLPHAPTLIKRTNRRLPERTSHRVADHAQVARVLGFFQCHSHPAQAFDEAMTQFGMSRHGLLQLFRRVGVTELEARSGTLPPAPQRWHRILQASGMKRAEPSGASAQTPDQASLHAFADALERELDAPSPIDRAGQALASSSRKRSRSESSVTGSFAQQAVEVRVPEQHEGLHFPPLSWGVKRPRTRIGGGLPDPGTPMDADLAASSTMIWEQDAAPFVGEADDFPAFNEEEMAWLMELFPQ |

| TAL21D_CFBP6164 |
| --- |
| MDPIRPRTPSPAHELLAGPQPDRVQPQPTADRGGAPPAGSPLDGLPARRTMSRTRLPSPPAPLPAFSAGSFSDLLRQFDPSLLDTSLFDSMSAFGAPHTEAASGEGDEVQSGLRAADDPQATVQVAVTAARPPRAKPVPRRRAAHTSDASPAGQVDLCTLGYSQQQQEKIKLKARSTVAQHHEALIGHGFTRAQIVALSQHPAALGTVAVKYQVMIAALPEATHEDIVGVGKQWSGARALEALLTVSGELRGPPLQLDTGQLLKIAKRGGVTAVEAVHAWRNALTGAPLNLTPDQVVAIASNIGGKQALETVQRLLPVLCEQHGLTPDQVVAIASNGGGKQALETVQRLLPVLCEQHGLTPDQVVAIASNIGGKQALETVQRLLPVLCEQHGLTPEQVVAIASNGGGKQALETVQRLLPVLCQAHGLTPDQVVAIASNIGGKQALETVQRLLPVLCEQHGLTPDQVVAIASNNGGKQALETVQRLLPVLCEQHGLTPDQVVAIASNGGGKQALETVQRLLPVLCEQHGLTREQVVAIASNNGGKQALETVQRLLPVLCQAHGLTPEQVVAIASHDGGKQALETVQRLLPVLCEQHGLTREQVVAIASHDGGKQALETLQRLLPVLCEQHGLTREQVVAIASNNGGKQALETVQRLLPVLCEQHGLTPAQVVAIASNNGGKQALETVQRLLPVLCEQHGLTPEQVVAIASHDGGKQALETVQRLLPVLCEQHGLTPDQVVAIASHDGGKQALETVQRLLPVLCEQHGLTREQVVAIASNIGGKQALETVQRLLPVLCEQHGLTPEQVVAIASHDGGKQALETVQRLLPVLCEQHGLTPEQVVAIASHDGGKQALETVQRLLPVLCQAHGLTPEQVVAIASHDGGKQALETVQRLLPVLCQAHGLTPEQVVAIASHDGGKQALETVQRLLPVLCQAHGLTPEQVVAIASNGGGKQALETVQRLLPVLCEQHGLTPDQVVAIASHDGGKQALESIFAQLSRPDQALAALTNDHLVALACLGGRPALEAVKKGLPHAPTLIKRTNRRLPERTSHRVADHAQVARVLGFFQCHSHPAQAFDEAMTQFGMSRHGLLQLFRRVGVTELEARSGTLPPAPQRWHRILQASGMKRAEPSGASAQTPDQASLHAFADALERELDAPSPIDRAGQALASSSRKRSRSESSVTGSFAQQAVEVRVPEQHEGLHFPPLSWGVKRPRTRIGGGLPDPGTPMDADLAASSTMIWEQDAAPFVGEADDFPAFNEEEMAWLMELFPQ |
| TAL18H_CFBP6164 |
| MDPIRPRAPSPAHELLAEPQPDRVQPQPTADRGGSPPAGSPLDGLPARRTMSRTRLPSPPAPLPAFSAGSFRDLLRQFDPSLLDTSLFDSMSAFGAPHTEAASGEGDEVQSGLRAADDPQATVQVAVTAARPPRAKPAPRRRAAHTSDASPAGQVDLCTLGYSQQQQEKIKLKARPIVAQHHEALIGHGFTRAHIVALSQHPAALGTVAVKYQAMIAALPEATHEDIVGVGKQWSGARALEALLTVSGELRGPPLQLDTGQLLKIAKRGGVTAVEAVHAWRNALTGVPLNLTPDQVVAIASNIGGKQALETVQLLLPVLCEQHGLTPDQVVAIASNGGGKQALETVQRLLPVLCKDHGLTPAQVVAIANHDGGKQALETVQRLLPVLCKDHGLTPAQVVAIASNGGGKQALETVEQLLPVLCKDHGLTPDQVVAIANHDGGKPALETVQRLLPVLCQELGLTPDQVVAIASNIGGKQALETVQRLLPVLCEQHGLTPDQVVAIASNGGAKQALETVQRLLPVLCQELGLTPAQVVAIASNIGGKQALETVQRLLPVLCEQHGLTPDQVVAIASHDGGKQALETVERLLPVLCQELGMTLAQVVAIASHYGGKQALEAVQRLLPVLCQELGLTPDQVVAIASNNGGKQALETVQRLLPVLCEQHGLTPDQVVAIASNGGKQALETVQRLLPVLCEQHGLTPDQVVAIASHDGGKQALETVERLLPVLCQELGMTLAQVVAIASNGGGKQALETVERLLPVLCQELGMTLAQVVAIASHYGGKQALEAVQRLLPVLCQDLGLTPDQVVAIASNNGGKQALETVQRLLPVLCEQHGLTPDQVVAIASHDGGKQALEAVQRLLPVLCQDHGLNPDQVVAIASNGGGRQALESIFAQLSRPDQALAALTNDHLVALACLGGCPALEAVKKGLPHALTLIKRTSRRLPERTSHRVADHAQVARVLGFFQCHSHPAQAFDEAMAQFAMSRHGLLQLFRRVGVTELEACNGTLPPASQRWHRILQASGVRTATPSRASAQTPDQASLDAFADALERGLDALSPIDQAVQAQASSRRKRSRSESSVTRSSAHYAVEVPVREQHAALDSLPPSWGAKRPRTRIGGGLADPGTPMHGDLAASSTVAWEHDAAPFAAAEAGDFPAFNDEEIAWLMELFPQ |
| TAL18H2_CFBP6164 |
| MRIRQGVEGPETFSLEATIFSIKRYARWIPFVRARQVLPTNFWPNPSRIGFSRSRLPDRGGSPPAGSPLDGLPARRTMSRTRLPSPPAPLPAFSAGSFRDLLRQFDPSLLDTSLFDSMSAFGAPHTEAASGEGDEVQSGLRAADDPQATVQVAVTAARPPRAKPAPRRRAAHTSDASPAGQVDLCTLGYSQQQQEKIKLKARPIVAQHHEALIGHGFTRAHIVALSQHPAALGTVAVKYQAMIAALPEATHEDIVGVGKQWSGARALEALLTVSGELRGPPLQLDTGQLLKIAKRGGVTAVEAVHAWRNALTGVPLNLTPDQVVAIASNIGGKQALETVQLLLPVLCEQHGLTPDQVVAIASNGGGKQALETVQRLLPVLCKDHGLTPAQVVAIANHDGGKQALETVQRLLPVLCKDHGLTPAQVVAIASNGGGKQALETVEQLLPVLCKDHGLTPDQVVAIANHDGGKPALETVQRLLPVLCQELGLTPDQVVAIASNIGGKQALETVQRLLPVLCEQHGLTPDQVVAIASNGGAKQALETVQRLLPVLCQELGLTPAQVVAIASNIGGKQALETVQRLLPVLCEQHGLTPDQVVAIASHDGGKQALETVERLLPVLCQELGMTLAQVVAIASHYGGKQALEAVQRLLPVLCQELGLTPDQVVAIASNNGGKQALETVQRLLPVLCEQHGLTPDQVVAIASNGGKQALETVQRLLPVLCEQHGLTPDQVVAIASHDGGKQALETVERLLPVLCQELGMTLAQVVAIASNGGGKQALETVERLLPVLCQELGMTLAQVVAIASHYGGKQALEAVQRLLPVLCQDLGLTPDQVVAIASNNGGKQALETVQRLLPVLCEQHGLTPDQVVAIASHDGGKQALEAVQRLLPVLCQDHGLNPDQVVAIASNGGGRQALESIFAQLSRPDQALAALTNDHLVALACLGGCPALEAVKKGLPHALTLIKRTSRRLPERTSHRVADHAQVARVLGFFQCHSHPAQAFDEAMAQFAMSRHGLLQLFRRVGVTELEACNGTLPPASQRWHRILQASGVRTATPSRASAQTPDQASLDAFADALERGLDALSPIDQAVQAQASSRRKRSRSESSVTRSSAHYAVEVPVREQHAALDSLPPSWGAKRPRTRIGGGLADPGTPMHGDLAASSTVAWEHDAAPFAAAEAGDFPAFNDEEIAWLMELFPQ |

| TAL19I_CFBP6546R |
| --- |
| MDPIRPRTPSPAHELLAGPQPDRVQPQPTADRGGAPPAGSPLDGLPARRTMSRTRLPSPPAPLPAFSAGSFSDLLRQFDPSLLDTSLFDSMSAFGAPHTEAASGEGDEVQSGLRAADDPQATVQVAVTAARPPRAKPVPRRRAAHTSDASPAGQVDLCTLGYSQQQQEKIKLKARSTVAQHHEALIGHGFTRAQIVALSQHPAALGTVAVKYQVMIAALPEATHEDIVGVGKQWSGARALEALLTVSGELRGPPLQLDTGQLLKIAKRGGVTAVEAVHAWRNALTGAPLNLTPDQVVAIASNIGGKQALETVQRLLPVLCEQHGLTPDQVVAIASNGGGKQALETVQRLLPVLCEQHGLTPDQVVAIASNIGGKQALETVQRLLPVLCEQHGLTPEQVVAIASNGGGKQALETVQRLLPVLCQAHGLTPDQVVAIASNIGGKQALETVQRLLPVLCEQHGLTPDQVVAIASNNGGKQALETVQRLLPVLCEQHGLTPDQVVAIASNGGGKQALETVQRLLPVLCEQHGLTREQVVAIASNNGGKQALETVQRLLPVLCQAHGLTPEQVVAIASHDGGKQALETVQRLLPVLCEQHGLTREQVVAIASHDGGKQALETLQRLLPVLCEQHGLTREQVVAIASNNGGKQALETVQRLLPVLCEQHGLTPAQVVAIASNNGGKQALETVQRLLPVLCEQHGLTPEQVVAIASHDGGKQALETVQRLLPVLCEQHGLTPDQVVAIASHDGGKQALETVQRLLPVLCEQHGLTREQVVAIASNIGGKQALETVQRLLPVLCEQHGLTPEQVVAIASHDGGKQALETVQRLLPVLCEQHGLTPEQVVAIASHDGGKQALETVQRLLPVLCQAHGLTPEQVVAIASNGGGKQALETVQRLLPVLCEQHGLTPDQVVAIASHDGGKQALESIFAQLSRPDQALAALTNDHLVALACLGGRPALEAVKKGLPHAPTLIKRTNRRLPERTSHRVADHAQVARVLGFFQCHSHPAQAFDEAMTQFGMSRHGLLQLFRRVGVTELEARSGTLPPAPQRWHRILQASGMKRAEPSGASAQTPDQASLHAFADALERELDAPSPIDRAGQALASSSRKRSRSESSVTGSFAQQAVEVRVPEQHEGLHFPPLSWGVKRPRTRIGGGLPDPGTPMDADLAASSTMIWEQDAAPFVGEADDFPAFNEEEMAWLMELFPQ |
| TAL18H_CFBP6546R |
| MDPIRPRAPSPAHELLAEPQPDRVQPQPTADRGGSPPAGSPLDGLPARRTMSRTRLPSPPAPLPAFSAGSFRDLLRQFDPSLLDTSLFDSMSAFGAPHTEAASGEGDEVQSGLRAADDPQATVQVAVTAARPPRAKPAPRRRAAHTSDASPAGQVDLCTLGYSQQQQEKIKLKARPIVAQHHEALIGHGFTRAHIVALSQHPAALGTVAVKYQAMIAALPEATHEDIVGVGKQWSGARALEALLTVSGELRGPPLQLDTGQLLKIAKRGGVTAVEAVHAWRNALTGVPLNLTPDQVVAIASNIGGKQALETVQLLLPVLCEQHGLTPDQVVAIASNGGGKQALETVQRLLPVLCKDHGLTPAQVVAIANHDGGKQALETVQRLLPVLCKDHGLTPAQVVAIASNGGGKQALETVEQLLPVLCKDHGLTPDQVVAIANHDGGKPALETVQRLLPVLCQELGLTPDQVVAIASNIGGKQALETVQRLLPVLCEQHGLTPDQVVAIASNGGAKQALETVQRLLPVLCQELGLTPAQVVAIASNIGGKQALETVQRLLPVLCEQHGLTPDQVVAIASHDGGKQALETVERLLPVLCQELGMTLAQVVAIASHYGGKQALEAVQRLLPVLCQELGLTPDQVVAIASNNGGKQALETVQRLLPVLCEQHGLTPDQVVAIASNGGKQALETVQRLLPVLCEQHGLTPDQVVAIASHDGGKQALETVERLLPVLCQELGMTLAQVVAIASNGGGKQALETVERLLPVLCQELGMTLAQVVAIASHYGGKQALEAVQRLLPVLCQDLGLTPDQVVAIASNNGGKQALETVQRLLPVLCEQHGLTPDQVVAIASHDGGKQALEAVQRLLPVLCQDHGLNPDQVVAIASNGGGRQALESIFAQLSRPDQALAALTNDHLVALACLGGCPALEAVKKGLPHALTLIKRTSRRLPERTSHRVADHAQVARVLGFFQCHSHPAQAFDEAMAQFAMSRHGLLQLFRRVGVTELEACNGTLPPASQRWHRILQASGVRTATPSRASAQTPDQASLDAFADALERGLDALSPIDQAVQAQASSRRKRSRSESSVTRSSAHYAVEVPVREQHAALDSLPPSWGAKRPRTRIGGGLADPGTPMHGDLAASSTVAWEHDAAPFAAAEAGDFPAFNDEEIAWLMELFPQ |

| TAL19E_CFBP6982 |
| --- |
| MDPIRPRTPSPAHELLAGPQPDRVQPQPTADRGGAPPAGSPLDGLPARRTMSRTRLPSPPAPLPAFSAGSFSDLLRQFDPSLLDTSLFDSMSAFGAPHTEAASGEGDEVQSGLRAADDPQATVQVAVTAARPPRAKPVPRRRAAHTSDASPAGQVDLCTLGYSQQQQEKIKLKARSTVAQHHEALIGHGFTRAQIVALSQHPAALGTVAVKYQVMIAALPEATHEDIVGVGKQWSGARALEALLTVSGELRGPPLQLDTGQLLKIAKRGGVTAVEAVHAWRNALTGAPLNLTPDQVVAIASNIGGKQALETVQRLLPVLCEQHGLTPDQVVAIASNGGGKQALETVQRLLPVLCEQHGLTPDQVVAIASNIGGKQALETVQRLLPVLCEQHGLTPEQVVAIASNGGGKQALETVQRLLPVLCQAHGLTPDQVVAIASNIGGKQALETVQRLLPVLCEQHGLTPDQVVAIASNNGGKQALETVQRLLPVLCEQHGLTPDQVVAIASNGGGKQALETVQRLLPVLCEQHGLTREQVVAIASNNGGKQALETVQRLLPVLCQAHGLTPEQVVAIASHDGGKQALETVQRLLPVLCEQHGLTREQVVAIASHDGGKQALETLQRLLPVLCEQHGLTREQVVAIASNNGGKQALETVQRLLPVLCEQHGLTPDQVVAIASNNGGKQALETVQRLLPVLCEQHGLTPDQVVAIASHDGGKQALETLQRLLPVLCEQHGLTREQVVAIASNIGGKQALETVQRLLPVLCEQHGLTPEQVVAIASHDGGKQALETVQRLLPVLCEQHGLTPEQVVAIASHDGGKQALETVQRLLPVLCQAHGLTPEQVVAIASHDGGKQALETVQRLLPVLCQAHGLTPEQVVAIASHDGGKQALETVQRLLPVLCEQHGLTPAQVVAIASNGGGKQALESIFAQLSRPDQALAALTNDHLVALACLGGRPALEAVKKGLPHAPTLIKRTNRRLPERTSHRVADHAQVARVLGFFQCHSHPAQAFDEAMTQFGMSRHGLLQLFRRVGVTELEARSGTLPPAPQRWHRILQASGMKRAEPSGASAQTPDQASLHAFADALERELDAPSPIDRAGQALASSSRKRSRSESSVTGSFAQQAVEVRVPEQHEGLHFPPLSWGVKRPRTRIGGGLPDPGTPMDADLAASSTMIWEQDAAPFVGEADDFPAFNEEEMAWLMELFPQ |
| TAL18H_CFBP6982 |
| MDPIRPRAPSPAHELLAEPQPDRVQPQPTADRGGSPPAGSPLDGLPARRTMSRTRLPSPPAPLPAFSAGSFRDLLRQFDPSLLDTSLFDSMSAFGAPHTEAASGEGDEVQSGLRAADDPQATVQVAVTAARPPRAKPAPRRRAAHTSDASPAGQVDLCTLGYSQQQQEKIKLKARPIVAQHHEALIGHGFTRAHIVALSQHPAALGTVAVKYQAMIAALPEATHEDIVGVGKQWSGARALEALLTVSGELRGPPLQLDTGQLLKIAKRGGVTAVEAVHAWRNALTGVPLNLTPDQVVAIASNIGGKQALETVQLLLPVLCEQHGLTPDQVVAIASNGGGKQALETVQRLLPVLCKDHGLTPAQVVAIANHDGGKQALETVQRLLPVLCKDHGLTPAQVVAIASNGGGKQALETVQRLLPVLCKDHGLTPDQVVAIANHDGGKPALETVQRLLPVLCQELGLTPDQVVAIASNIGGKQALETVQRLLPVLCEQHGLTPDQVVAIASNGGAKQALETVQRLLPVLCQELGLTPAQVVAIASNIGGKQALETVQRLLPVLCEQHGLTPDQVVAIASHDGGKQALETVERLLPVLCQELGMTLAQVVAIASHYGGKQALEAVQRLLPVLCQELGLTPDQVVAIASNNGGKQALETVQRLLPVLCEQHGLTPDQVVAIASNGGKQALETVQRLLPVLCEQHGLTPDQVVAIASHDGGKQALETVERLLPVLCQELGMTLAQVVAIASNGGGKQALETVERLLPVLCQELGMTLAQVVAIASHYGGKQALEAVQRLLPVLCQDLGLTPDQVVAIASNNGGKQALETVQRLLPVLCEQHGLTPDQVVAIASHDGGKQALEAVQRLLPVLCQDHGLNPDQVVAIASNGGGRQALESIFAQLSRPDQALAALTNDHLVALACLGGCPALEAVKKGLPHALTLIKRTSRRLPERTSHRVADHAQVARVLGFFQCHSHPAQAFDEAMAQFAMSRHGLLQLFRRVGVTELEACNGTLPPASQRWHRILQASGVRTATPSRASAQTPDQASLDAFADALERGLDALSPIDQAVQAQASSRRKRSRSESSVTRSSAHYAVEVPVREQHAALDSLPPSWGAKRPRTRIGGGLADPGTPMHGDLAASSTVAWEHDAAPFAAAEAGDFPAFNDEEIAWLMELFPQ |
