# Supplementary material for: Xanthomonas adaptation to common bean is associated with horizontal transfers of genes encoding TAL effectors
Source: BMC Genomics. 2017 Aug 30;18:670. doi: 10.1186/s12864-017-4087-6 (PMC5577687; doi:10.1186/s12864-017-4087-6)
Supplement: Supplementary file 3 — Gel photograph showing the sizes obtained for tal23A_CFBP4885 (Xfutal1) and tal18H_CFBP4885 (Xfutal2) after PCR amplification. Amplification was performed using XapF2 and XapR primers on plasmidic DNA from the CFBP4885 strain (Additional file 18: Table S11). Expected PCR product sizes for tal23A_CFBP4885 and tal18H_CFBP4885 were 4604 bp and 3074 bp for Sanger sequencing of the entire genome, respectively, and 3686 bp and 3176 bp for SMRT sequencing of the entire genome, respectively. (DOCX 2788 kb) [file 12864_2017_4087_MOESM3_ESM.docx]

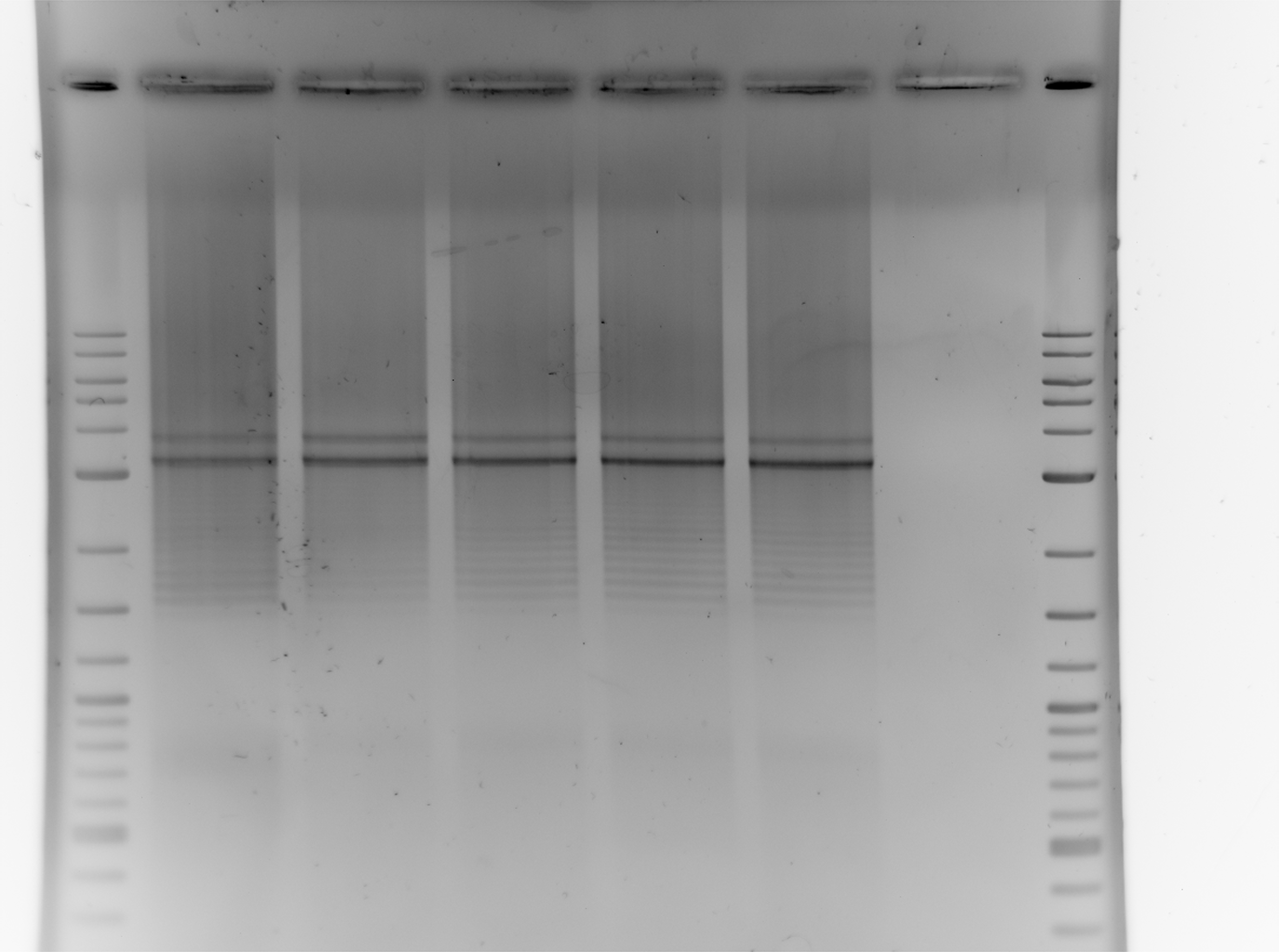


*tal18H_CFBP4885*

*tal23A_CFBP4885*

3 kbp

4 kbp

**2-Log DNA Ladder**

Negative control

**2-Log DNA Ladder**

CFBP4885

**Additional file 3: Figure S1.** Gel photograph showing the sizes obtained for *tal23A_CFBP4885* (*Xfutal1*) and *tal18H_CFBP4885* (*Xfutal2*) after PCR amplification. Amplification was performed using XapF2 and XapR primers on plasmidic DNA from the CFBP4885 strain (Additional file 18: Table S11). Expected PCR product sizes for *tal23A_CFBP4885* and *tal18H_CFBP4885* were 4604 bp and 3074 bp after Sanger genome sequencing, respectively, and 3686 bp and 3176 bp for SMRT sequencing, respectively.
