# Supplementary material for: Xanthomonas adaptation to common bean is associated with horizontal transfers of genes encoding TAL effectors
Source: BMC Genomics. 2017 Aug 30;18:670. doi: 10.1186/s12864-017-4087-6 (PMC5577687; doi:10.1186/s12864-017-4087-6)
Supplement: Supplementary file 4 — RVD frequencies in TAL effectors from the 17 X. citri pv. fuscans and X. phaseoli pv. phaseoli strains used in this study. (DOCX 20 kb) [file 12864_2017_4087_MOESM4_ESM.docx]

**Additional file 4: Figure S2.** RVD frequencies in TAL effectors from the 17 *X. citri* pv. *fuscans* and *X. phaseoli* pv. *phaseoli* strains used in this study.
