# Supplementary material for: Xanthomonas adaptation to common bean is associated with horizontal transfers of genes encoding TAL effectors
Source: BMC Genomics. 2017 Aug 30;18:670. doi: 10.1186/s12864-017-4087-6 (PMC5577687; doi:10.1186/s12864-017-4087-6)
Supplement: Supplementary file 7 — Phylogenetic trees of N-ter and C-ter-encoding regions of tal genes with sequences published by Aritua et al. (2015). Bootstrap values greater than 50% (100 replicates) are shown and horizontal scale bars represent the number of nucleotide substitutions per site. The tal genes from the X. citri pv. fuscans genetic lineages fuscans, NF2 and NF3 are indicated in red, X. phaseoli pv. phaseoli NF1 lineage in blue, X. citri pv. aurantifolii in pink and X. phaseoli pv. manihotis in purple. The sequences published by Aritua et al. (2015) are in bold. Both ML trees were constructed using tal genes from X. translucens pv. undulosa XT4699 as outgroups. a ML tree constructed on a nucleotide alignment of the N-ter-encoding region of tal genes. b ML tree constructed on a nucleotide alignment of the C-ter-encoding region of tal genes. (PPTX 137 kb) [file 12864_2017_4087_MOESM7_ESM.pptx]

## Slide 1
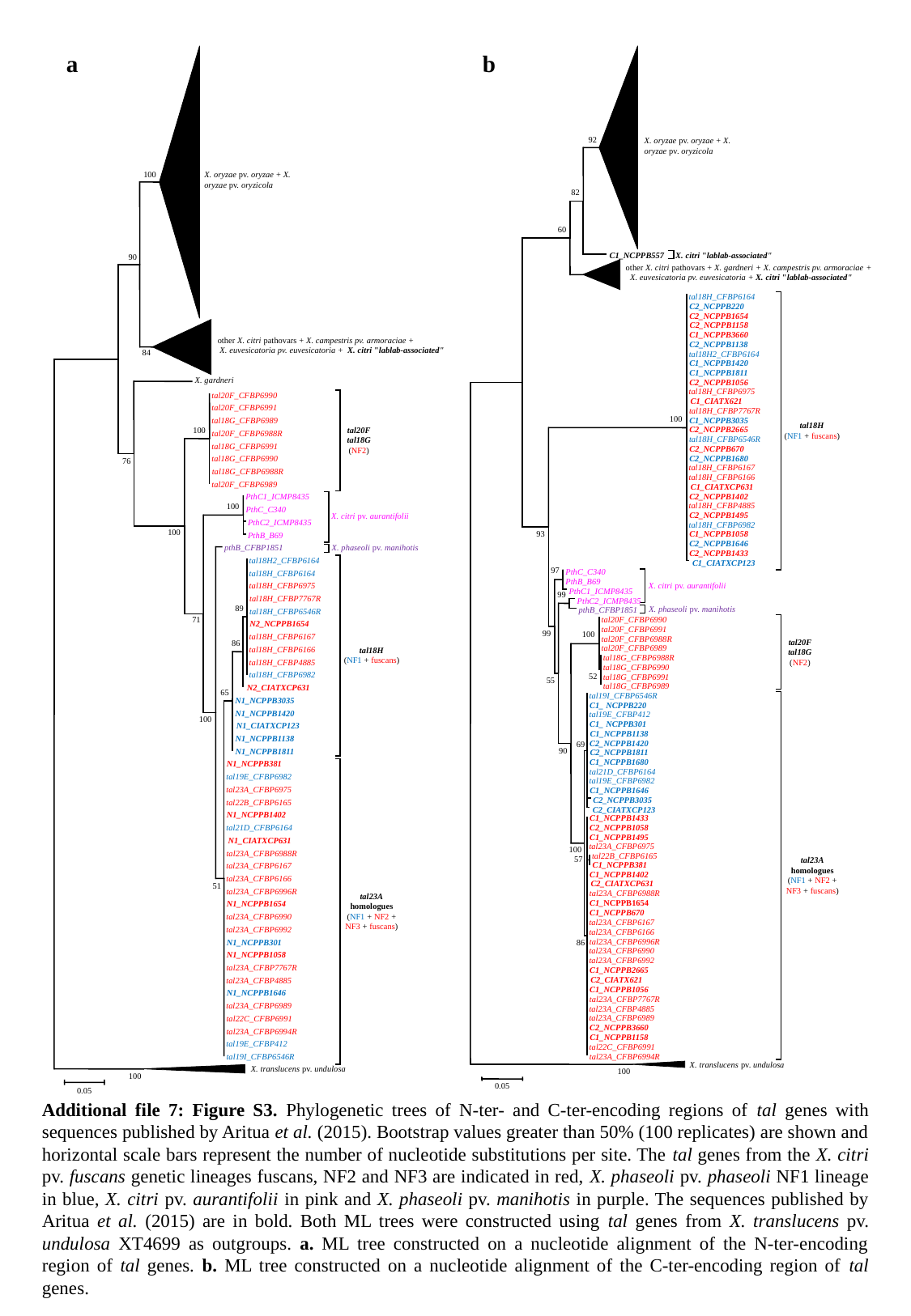

a
100
X. oryzae pv. oryzae + X. oryzae pv. oryzicola
90
 other X. citri pathovars + X. campestris pv. armoraciae +
 X. euvesicatoria pv. euvesicatoria + X. citri "lablab-associated"
84
 X. gardneri
 tal20F_CFBP6990
 tal20F_CFBP6991
 tal18G_CFBP6989
100
 tal20F
tal18G
(NF2)
 tal20F_CFBP6988R
 tal18G_CFBP6991
 tal18G_CFBP6990
76
 tal18G_CFBP6988R
 tal20F_CFBP6989
 PthC1_ICMP8435
100
 PthC_C340
X. citri pv. aurantifolii
 PthC2_ICMP8435
100
 PthB_B69
X. phaseoli pv. manihotis
 pthB_CFBP1851
 tal18H2_CFBP6164
 tal18H_CFBP6164
 tal18H_CFBP6975
 tal18H_CFBP7767R
89
 tal18H_CFBP6546R
71
 N2_NCPPB1654
 tal18H_CFBP6167
86
 tal18H_CFBP6166
tal18H
(NF1 + fuscans)
 tal18H_CFBP4885
 tal18H_CFBP6982
 N2_CIATXCP631
65
 N1_NCPPB3035
 N1_NCPPB1420
100
 N1_CIATXCP123
 N1_NCPPB1138
 N1_NCPPB1811
 N1_NCPPB381
 tal19E_CFBP6982
 tal23A_CFBP6975
 tal22B_CFBP6165
 N1_NCPPB1402
 tal21D_CFBP6164
 N1_CIATXCP631
 tal23A_CFBP6988R
 tal23A_CFBP6167
 tal23A_CFBP6166
51
 tal23A_CFBP6996R
tal23A homologues
(NF1 + NF2 + NF3 + fuscans)
 N1_NCPPB1654
 tal23A_CFBP6990
 tal23A_CFBP6992
 N1_NCPPB301
 N1_NCPPB1058
 tal23A_CFBP7767R
 tal23A_CFBP4885
 N1_NCPPB1646
 tal23A_CFBP6989
 tal22C_CFBP6991
 tal23A_CFBP6994R
 tal19E_CFBP412
 tal19I_CFBP6546R
 X. translucens pv. undulosa
100
0.05
b
92
X. oryzae pv. oryzae + X. oryzae pv. oryzicola
82
60
 C1_NCPPB557
X. citri "lablab-associated"
other X. citri pathovars + X. gardneri + X. campestris pv. armoraciae +
 X. euvesicatoria pv. euvesicatoria + X. citri "lablab-associated"
 tal18H_CFBP6164
 C2_NCPPB220
 C2_NCPPB1654
 C2_NCPPB1158
 C1_NCPPB3660
 C2_NCPPB1138
 tal18H2_CFBP6164
 C1_NCPPB1420
 C1_NCPPB1811
 C2_NCPPB1056
 tal18H_CFBP6975
 C1_CIATX621
 tal18H_CFBP7767R
100
 C1_NCPPB3035
tal18H
(NF1 + fuscans)
 C2_NCPPB2665
 tal18H_CFBP6546R
 C2_NCPPB670
 C2_NCPPB1680
 tal18H_CFBP6167
 tal18H_CFBP6166
 C1_CIATXCP631
 C2_NCPPB1402
 tal18H_CFBP4885
 C2_NCPPB1495
 tal18H_CFBP6982
93
 C1_NCPPB1058
 C2_NCPPB1646
 C2_NCPPB1433
 C1_CIATXCP123
97
 PthC_C340
 PthB_B69
X. citri pv. aurantifolii
 PthC1_ICMP8435
99
 PthC2_ICMP8435
X. phaseoli pv. manihotis
 pthB_CFBP1851
 tal20F_CFBP6990
 tal20F_CFBP6991
99
100
 tal20F_CFBP6988R
 tal20F
tal18G
(NF2)
 tal20F_CFBP6989
 tal18G_CFBP6988R
 tal18G_CFBP6990
52
 tal18G_CFBP6991
55
 tal18G_CFBP6989
 tal19I_CFBP6546R
 C1_ NCPPB220
 tal19E_CFBP412
 C1_ NCPPB301
 C1_NCPPB1138
 C2_NCPPB1420
69
90
 C2_NCPPB1811
 C1_NCPPB1680
 tal21D_CFBP6164
 tal19E_CFBP6982
 C1_NCPPB1646
 C2_NCPPB3035
 C2_CIATXCP123
 C1_NCPPB1433
 C2_NCPPB1058
 C1_NCPPB1495
 tal23A_CFBP6975
100
 tal22B_CFBP6165
57
tal23A homologues
(NF1 + NF2 + NF3 + fuscans)
 C1_NCPPB381
 C1_NCPPB1402
 C2_CIATXCP631
 tal23A_CFBP6988R
 C1_NCPPB1654
 C1_NCPPB670
 tal23A_CFBP6167
 tal23A_CFBP6166
 tal23A_CFBP6996R
86
 tal23A_CFBP6990
 tal23A_CFBP6992
 C1_NCPPB2665
 C2_CIATX621
 C1_NCPPB1056
 tal23A_CFBP7767R
 tal23A_CFBP4885
 tal23A_CFBP6989
 C2_NCPPB3660
 C1_NCPPB1158
 tal22C_CFBP6991
 tal23A_CFBP6994R
 X. translucens pv. undulosa
100
0.05
Additional file 7: Figure S3. Phylogenetic trees of N-ter- and C-ter-encoding regions of tal genes with sequences published by Aritua et al. (2015). Bootstrap values greater than 50% (100 replicates) are shown and horizontal scale bars represent the number of nucleotide substitutions per site. The tal genes from the X. citri pv. fuscans genetic lineages fuscans, NF2 and NF3 are indicated in red, X. phaseoli pv. phaseoli NF1 lineage in blue, X. citri pv. aurantifolii in pink and X. phaseoli pv. manihotis in purple. The sequences published by Aritua et al. (2015) are in bold. Both ML trees were constructed using tal genes from X. translucens pv. undulosa XT4699 as outgroups. a. ML tree constructed on a nucleotide alignment of the N-ter-encoding region of tal genes. b. ML tree constructed on a nucleotide alignment of the C-ter-encoding region of tal genes.
