# Supplementary material for: Xanthomonas adaptation to common bean is associated with horizontal transfers of genes encoding TAL effectors
Source: BMC Genomics. 2017 Aug 30;18:670. doi: 10.1186/s12864-017-4087-6 (PMC5577687; doi:10.1186/s12864-017-4087-6)
Supplement: Supplementary file 8 — Circular representation of plasmids A and C. Genomic sequences were compared and converted in a graphical map using CGView (Grant et al., 2012) with strain CFBP4885 as reference. Colours differ according to identity percentage (see legend). Strains order, ANIb and alignment percentages are indicated in the center of each graphical map. Localisation of tal genes are highlighted by green zones. Absence of tal genes in plasmid A from strains CFBP6164 and CFBP6546R is indicated by a black cross. (PDF 444 kb) [file 12864_2017_4087_MOESM8_ESM.pdf]

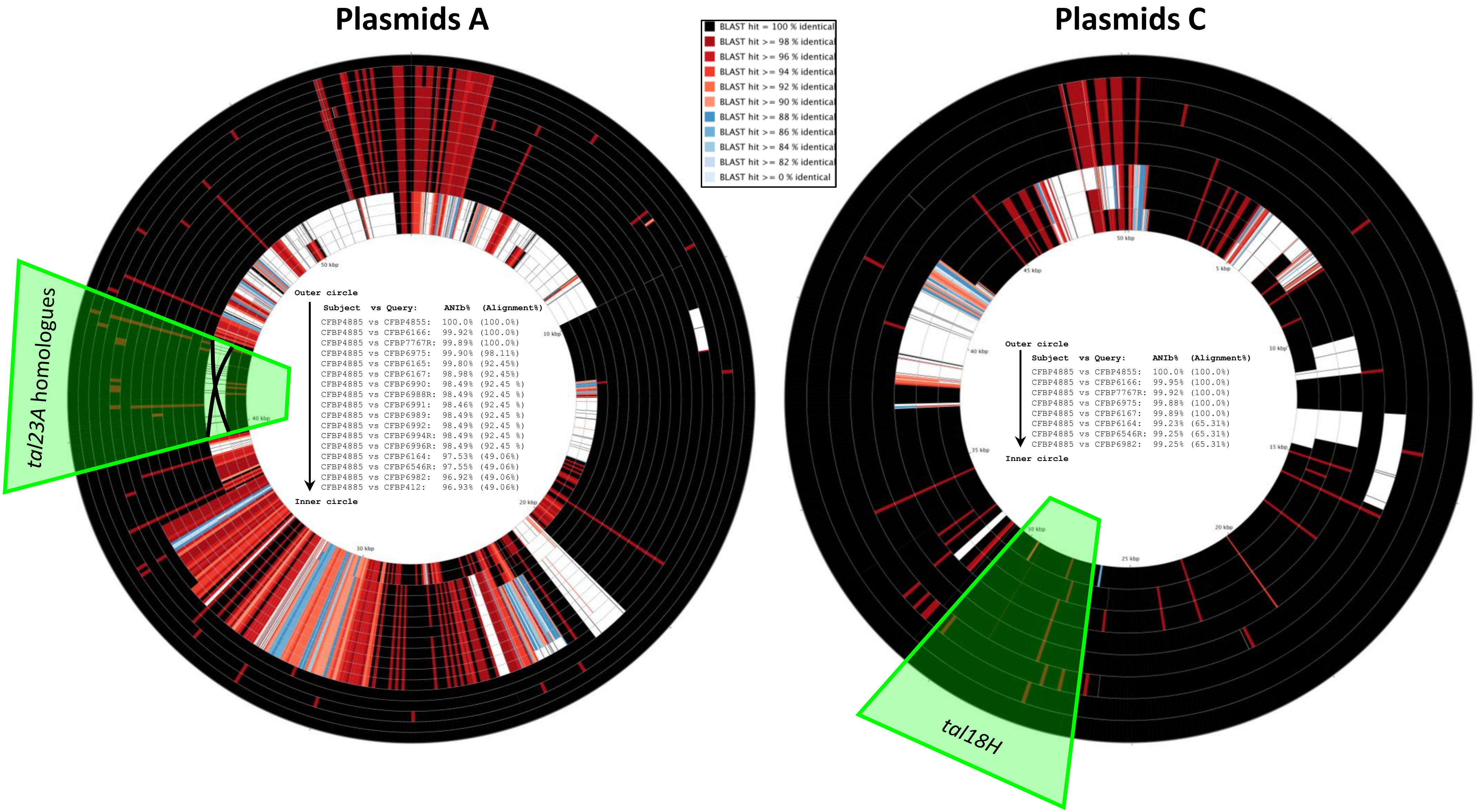

Additional file 8: Figure S4. Circular representation of plasmids A and C. Genomic sequences were compared and converted in a graphical map using CGView (Grant *et al.*, 2012) with strain CFBP4885 as reference. Colours differ according to identity percentage (see legend). Strains order, ANIb and alignment percentages are indicated in the center of each graphical map. Localisation of *tal* genes are highlighted by green zones. Absence of *tal* genes in plasmid A from strains CFBP6164 and CFBP6546R is indicated by a black cross.
