# Supplementary material for: Xanthomonas adaptation to common bean is associated with horizontal transfers of genes encoding TAL effectors
Source: BMC Genomics. 2017 Aug 30;18:670. doi: 10.1186/s12864-017-4087-6 (PMC5577687; doi:10.1186/s12864-017-4087-6)
Supplement: Supplementary file 10 — Circular representation of the regions shared by phylogenetically distant lineages in plasmids A and C. Genomic sequences were compared and converted in a graphical map using CGView (Grant et al., 2012). Colours differ according to identity percentage (see legend). Regions shared by both strains are surrounded by a black line. Numbers represent genes conserved in both strains (see Additional files 11 and 13 for more details). a Plasmids A from strains CFBP6988R (outer circle = reference) and CFBP6546R (inner circle). b Plasmids C from strains CFBP6166 (outer circle = reference) and CFBP6982 (inner circle). (PDF 168 kb) [file 12864_2017_4087_MOESM10_ESM.pdf]

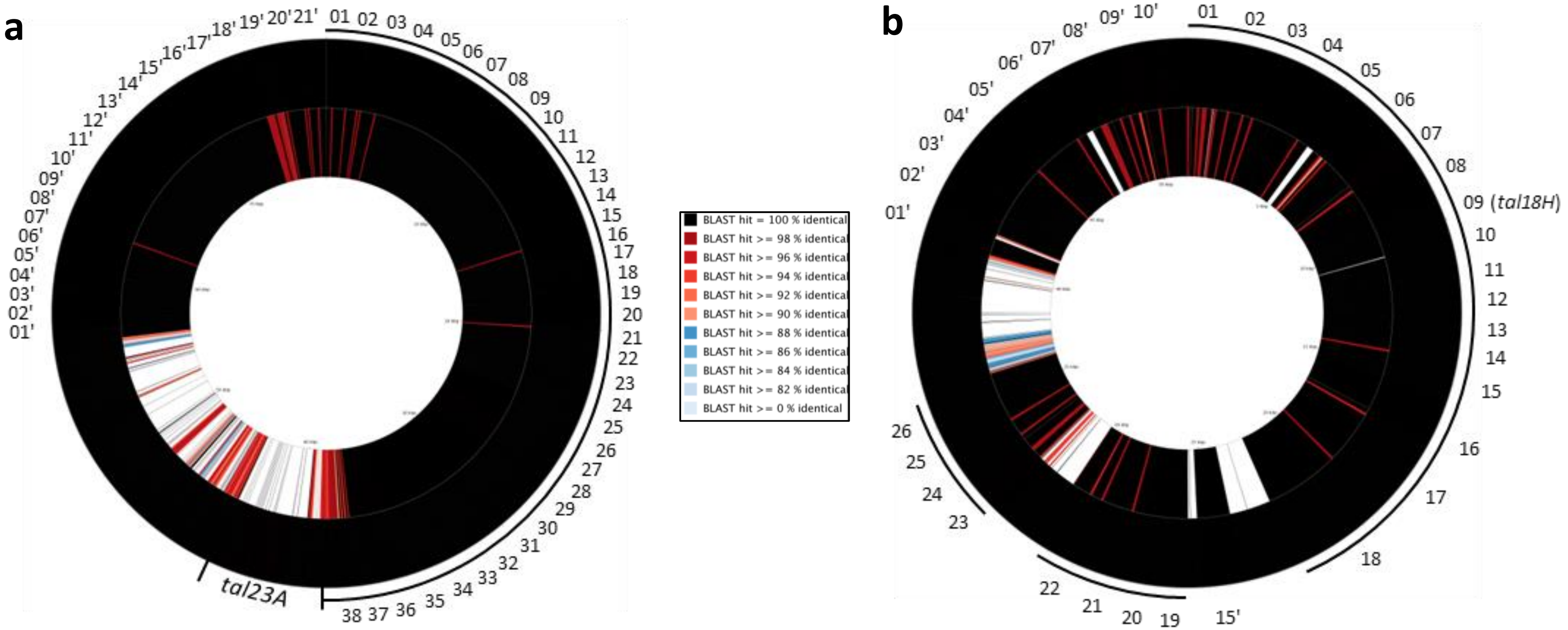

Additional file 10: Figure S5. Circular representation of the regions shared by phylogenetically distant lineages in plasmids A and C. Genomic sequences were compared and converted in a graphical map using CGView (Grant *et al*., 2012). Colours differ according to identity percentage (see legend). Non-redundant regions shared by both strains are surrounded by a black line. Numbers represent genes conserved in both strains (see Additional files 11 and 13 for more details). **a**. Plasmids A from strains CFBP6988R (outer circle = reference) and CFBP6546R (inner circle). **b**. Plasmids C from strains CFBP6166 (outer circle = reference) and CFBP6982 (inner circle).
