# Supplementary material for: Xanthomonas adaptation to common bean is associated with horizontal transfers of genes encoding TAL effectors
Source: BMC Genomics. 2017 Aug 30;18:670. doi: 10.1186/s12864-017-4087-6 (PMC5577687; doi:10.1186/s12864-017-4087-6)
Supplement: Supplementary file 14 — Duplications and movements of tal genes associated with IS3 and Tn3-like transposons. Arrows represent tal genes (yellow), IS3 sequences (orange) or Tn3-like transposons (dark blue). Arrowheads correspond to truncated IS3 (orange) or Tn3-like transposon (dark blue). Striped areas correspond to conserved regions. Dashed lines represent the borders of duplication events between different regions of a single molecule. a Movement of tal23A homologues from the plasmid A to the chromosome associated with IS3 sequences. b Duplication of tal20F and tal18G associated with Tn3-like transposons. c. Duplication of tal18H_CFBP6164 associated with IS3 sequences in strain CFBP6164. (PPTX 74 kb) [file 12864_2017_4087_MOESM14_ESM.pptx]

## Slide 1
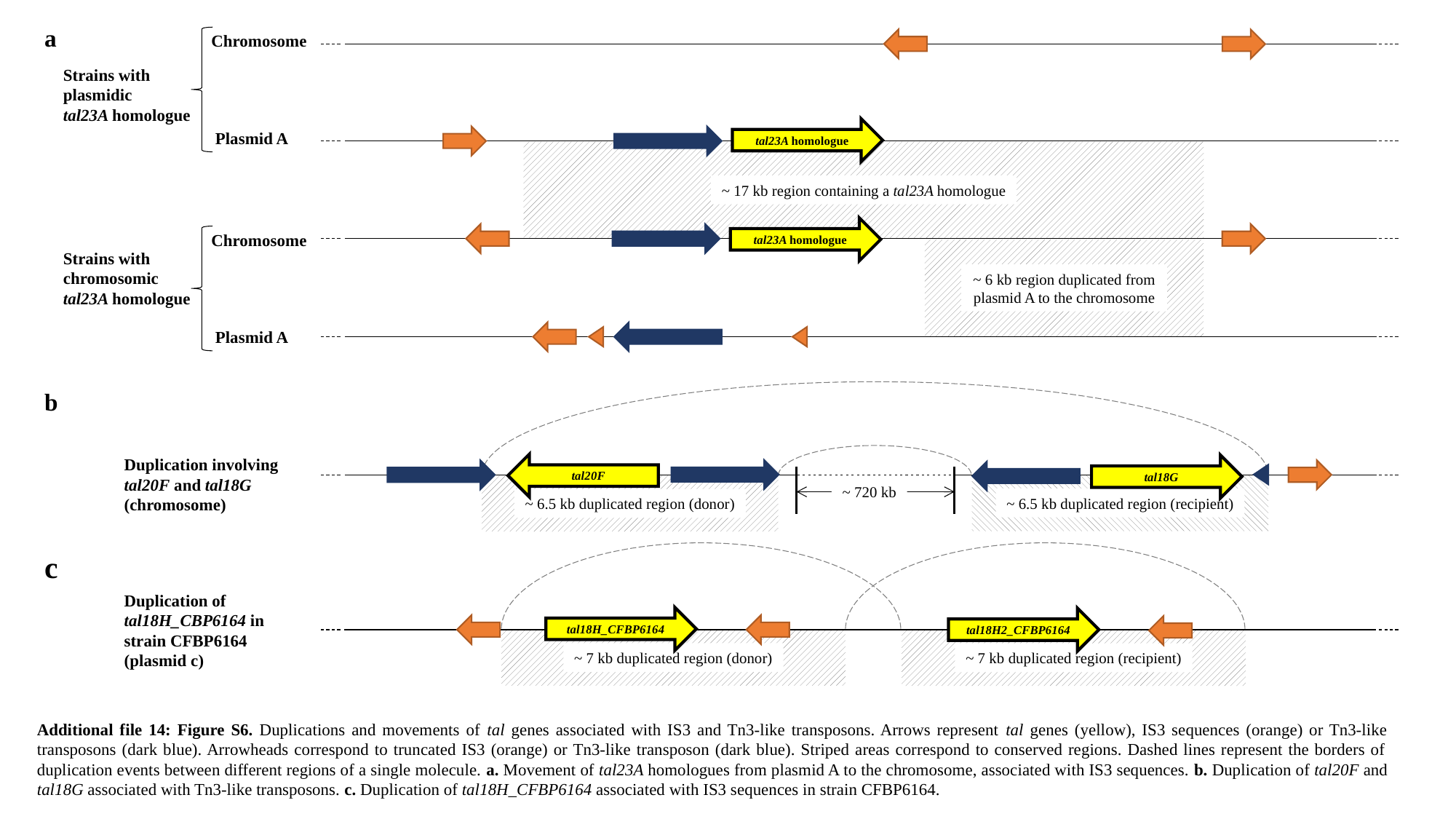

a
Chromosome
Strains with
plasmidic
tal23A homologue
Plasmid A
tal23A homologue
~ 17 kb region containing a tal23A homologue
tal23A homologue
Chromosome
Strains with
chromosomic
tal23A homologue
~ 6 kb region duplicated from plasmid A to the chromosome
Plasmid A
b
Duplication involving tal20F and tal18G (chromosome)
tal20F
tal18G
~ 720 kb
~ 6.5 kb duplicated region (recipient)
~ 6.5 kb duplicated region (donor)
c
Duplication of tal18H_CBP6164 in strain CFBP6164 (plasmid c)
tal18H_CFBP6164
tal18H2_CFBP6164
~ 7 kb duplicated region (donor)
~ 7 kb duplicated region (recipient)
Additional file 14: Figure S6. Duplications and movements of tal genes associated with IS3 and Tn3-like transposons. Arrows represent tal genes (yellow), IS3 sequences (orange) or Tn3-like transposons (dark blue). Arrowheads correspond to truncated IS3 (orange) or Tn3-like transposon (dark blue). Striped areas correspond to conserved regions. Dashed lines represent the borders of duplication events between different regions of a single molecule. a. Movement of tal23A homologues from plasmid A to the chromosome, associated with IS3 sequences. b. Duplication of tal20F and tal18G associated with Tn3-like transposons. c. Duplication of tal18H_CFBP6164 associated with IS3 sequences in strain CFBP6164.
