# Supplementary material for: Xanthomonas adaptation to common bean is associated with horizontal transfers of genes encoding TAL effectors
Source: BMC Genomics. 2017 Aug 30;18:670. doi: 10.1186/s12864-017-4087-6 (PMC5577687; doi:10.1186/s12864-017-4087-6)
Supplement: Supplementary file 16 — Repeats conserved and shared between different TAL effectors from X. citri pv. fuscans, X. phaseoli pv. phaseoli, X. citri pv. aurantifolii and X. phaseoli pv. manihotis strains. Repeats conserved between TAL effectors from different groups are indicated by the same colour (red, purple, green, blue or yellow). Half-coloured boxes indicate that polymorphism exist among TAL effectors homologues from different strains. Please see Additional file 15: Table S9 for more detailed information. (PPTX 69 kb) [file 12864_2017_4087_MOESM16_ESM.pptx]

## Slide 1
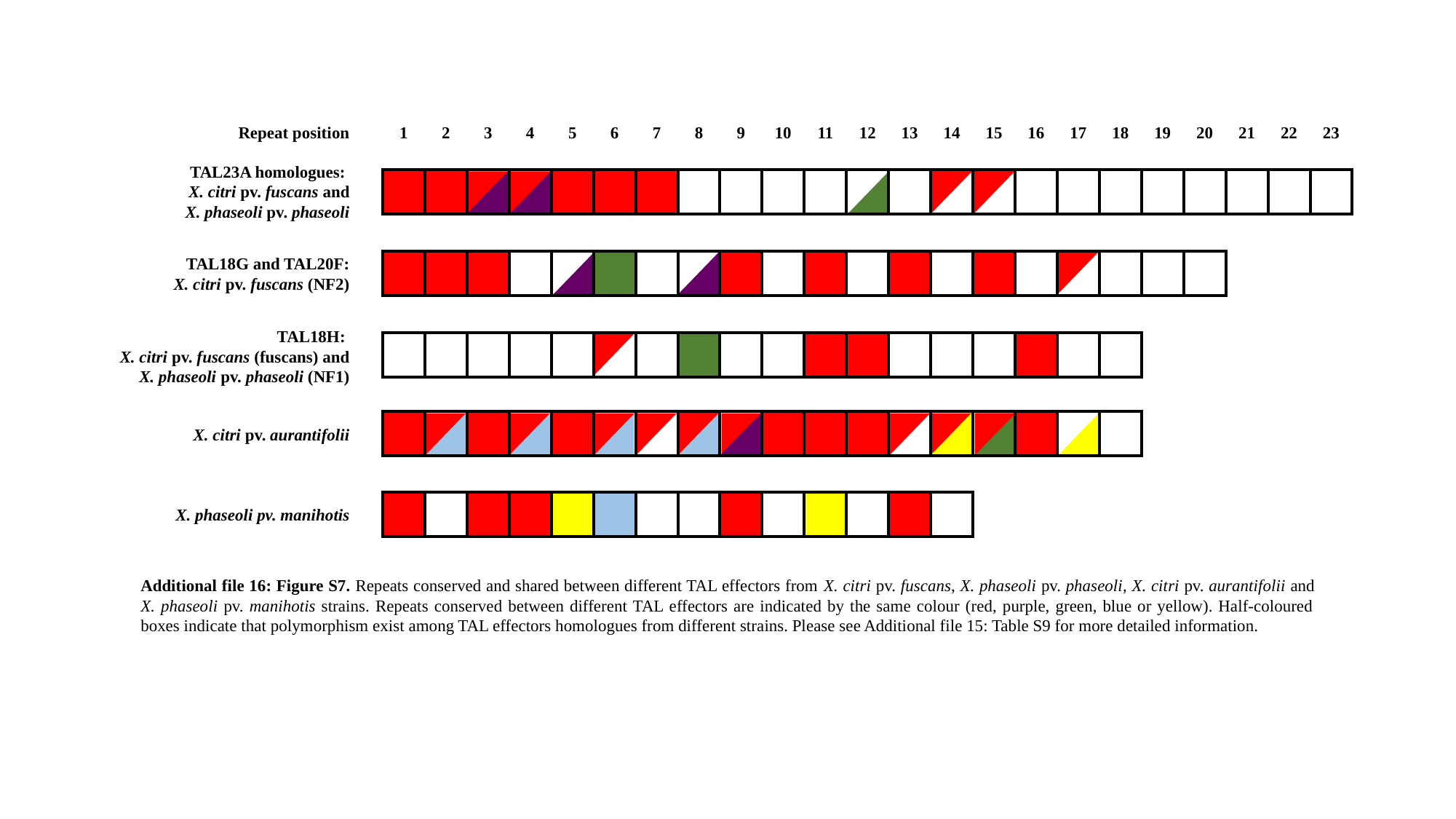

| 1 | 2 | 3 | 4 | 5 | 6 | 7 | 8 | 9 | 10 | 11 | 12 | 13 | 14 | 15 | 16 | 17 | 18 | 19 | 20 | 21 | 22 | 23 |
| --- | --- | --- | --- | --- | --- | --- | --- | --- | --- | --- | --- | --- | --- | --- | --- | --- | --- | --- | --- | --- | --- | --- |
Repeat position
TAL23A homologues:
X. citri pv. fuscans and X. phaseoli pv. phaseoli
| | | | | | | | | | | | | | | | | | | | | | | |
| --- | --- | --- | --- | --- | --- | --- | --- | --- | --- | --- | --- | --- | --- | --- | --- | --- | --- | --- | --- | --- | --- | --- |
TAL18G and TAL20F:
 X. citri pv. fuscans (NF2)
| | | | | | | | | | | | | | | | | | | | |
| --- | --- | --- | --- | --- | --- | --- | --- | --- | --- | --- | --- | --- | --- | --- | --- | --- | --- | --- | --- |
TAL18H:
X. citri pv. fuscans (fuscans) and X. phaseoli pv. phaseoli (NF1)
| | | | | | | | | | | | | | | | | | |
| --- | --- | --- | --- | --- | --- | --- | --- | --- | --- | --- | --- | --- | --- | --- | --- | --- | --- |
| | | | | | | | | | | | | | | | | | |
| --- | --- | --- | --- | --- | --- | --- | --- | --- | --- | --- | --- | --- | --- | --- | --- | --- | --- |
X. citri pv. aurantifolii
| | | | | | | | | | | | | | |
| --- | --- | --- | --- | --- | --- | --- | --- | --- | --- | --- | --- | --- | --- |
X. phaseoli pv. manihotis
Additional file 16: Figure S7. Repeats conserved and shared between different TAL effectors from X. citri pv. fuscans, X. phaseoli pv. phaseoli, X. citri pv. aurantifolii and X. phaseoli pv. manihotis strains. Repeats conserved between different TAL effectors are indicated by the same colour (red, purple, green, blue or yellow). Half-coloured boxes indicate that polymorphism exist among TAL effectors homologues from different strains. Please see Additional file 15: Table S9 for more detailed information.
