# Supplementary material for: Xanthomonas adaptation to common bean is associated with horizontal transfers of genes encoding TAL effectors
Source: BMC Genomics. 2017 Aug 30;18:670. doi: 10.1186/s12864-017-4087-6 (PMC5577687; doi:10.1186/s12864-017-4087-6)
Supplement: Supplementary file 18 — Primers used in this study. (DOCX 19 kb) [file 12864_2017_4087_MOESM18_ESM.docx]

**Additional file 18: Table S11.** Primers used in this study*.*

| Primer name | Designed on strain | *tal* genes | Localisation | Sequence | Product size | Used in strains |
| --- | --- | --- | --- | --- | --- | --- |
| Xap-F2 | CFBP4885 | *tal23A_CFBP4885* and *tal18H_CFBP4885* | N-ter | CATACAGAGGCTGCCTCAG | 3686 bp or 3176 bp | CFBP4885 |
| Xap-R | CFBP4885 | *tal23A_CFBP4885* and *tal18H_CFBP4885* | C-ter | TCGTTGAATGCCGGGAAATC |  |  |
| F7767C23 | CFBP7767R | *tal23A_CFBP7767R* | C-ter | GCAGGCACGGGTTGTTACAGC | 479 bp | CFBP4885; CFBP7767R; CFBP6546R; CFBP6994R |
| R7767C23 | CFBP7767R | *tal23A_CFBP7767R* | C-ter | GGCCGCATCTTGTTCCCAGATC |  |  |
| F7767C18 | CFBP7767R | *tal18H_CFBP7767R* | C-ter | CGGCACGGTTTGTTACAACTCTTTC | 469 bp | CFBP4885; CFBP6546R; CFBP7767R |
| R7767C18 | CFBP7767R | *tal18H_CFBP7767R* | C-ter | CGTGTTCCCAAGCCACGGTG |  |  |
| F7767N | CFBP7767R | *tal18H_CFBP7767R* | upstream of the gene | GATGGGTTCAGGATCGCC | 691 bp | CFBP6546R; CFBP7767R |
| R7767N | CFBP7767R | *tal18H_CFBP7767R* | N-ter | CTATCGGACGAGCCTTCAG |  |  |
| F6988N18 | CFBP6988R | *tal18G_CFBP6988R* | N-ter | GGTGAAGAATTGCAGCCTCCGAC | 2740 bp | CFBP6988R |
| R6988N | CFBP6988R | *tal18G_CFBP6988R* | upstream of the gene | GAGCGCCGAAGGCAGGC |  |  |
