# Supplementary material for: Xanthomonas adaptation to common bean is associated with horizontal transfers of genes encoding TAL effectors
Source: BMC Genomics. 2017 Aug 30;18:670. doi: 10.1186/s12864-017-4087-6 (PMC5577687; doi:10.1186/s12864-017-4087-6)
Supplement: Supplementary file 19 — Phylogenetic trees of N-ter- and C-ter-encoding regions of tal genes used for the Kishino-Hasegawa-Templeton test. Bootstrap values greater than 50% are shown for 1000 replicates and horizontal scale bars represent the number of nucleotide substitutions per site. a ML tree constructed on a nucleotide alignment of the N-ter-encoding region of tal genes from X. citri pv. fuscans, X. citri pv. aurantifolii, X. phaseoli pv. phaseoli and X. phaseoli pv. manihotis strains using a tal gene from X. translucens pv. undulosa XT4699 as outgroup. b ML tree constructed on a nucleotide alignment of the C-ter-encoding region of tal genes from X. citri pv. fuscans, X. citri pv. aurantifolii, X. phaseoli pv. phaseoli and X. phaseoli pv. manihotis strains using a tal gene from X. translucens pv. undulosa XT4699 as outgroup. (PPTX 56 kb) [file 12864_2017_4087_MOESM19_ESM.pptx]

## Slide 1
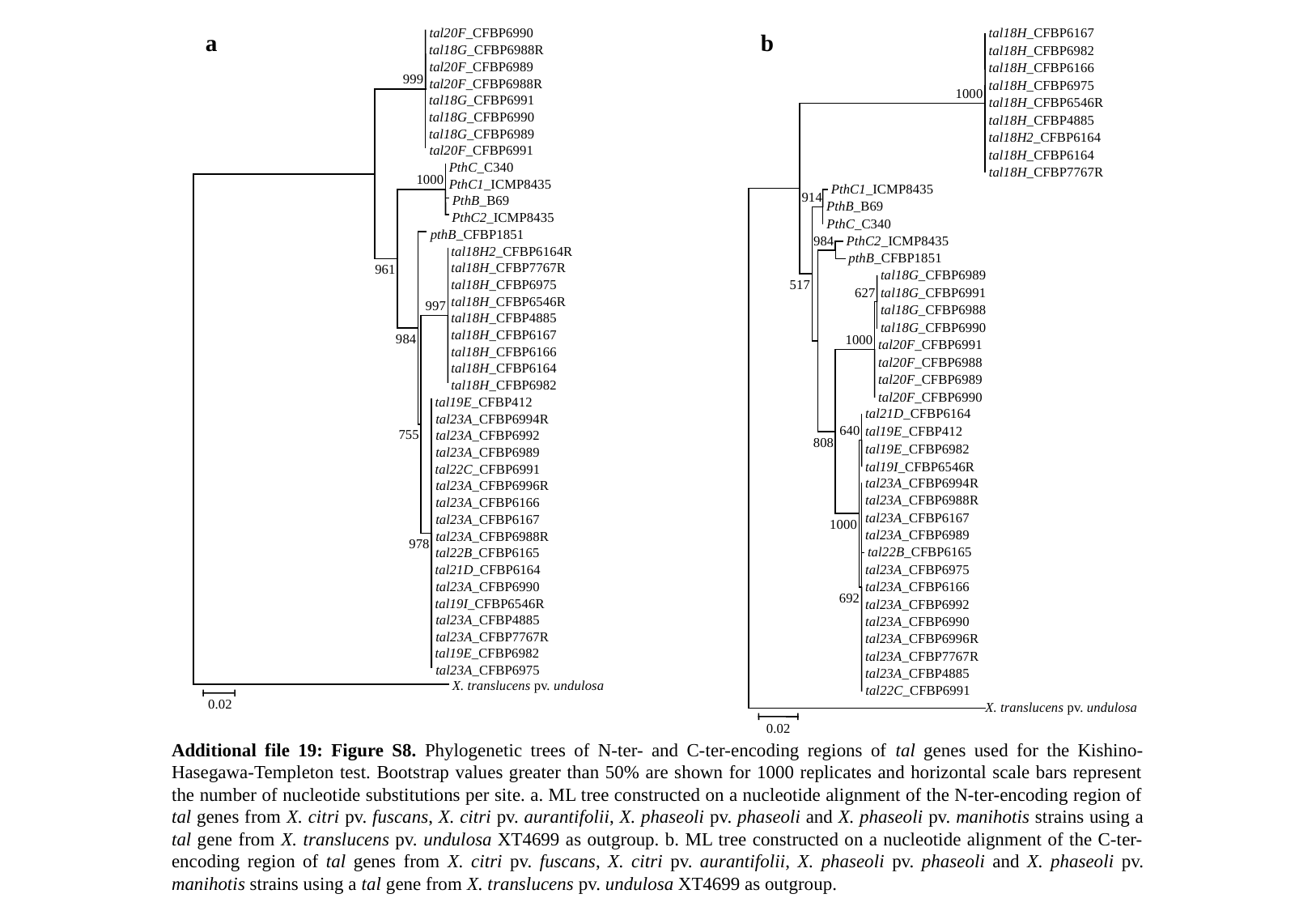

a
 tal20F_CFBP6990
 tal18G_CFBP6988R
 tal20F_CFBP6989
999
 tal20F_CFBP6988R
 tal18G_CFBP6991
 tal18G_CFBP6990
 tal18G_CFBP6989
 tal20F_CFBP6991
 PthC_C340
1000
 PthC1_ICMP8435
 PthB_B69
 PthC2_ICMP8435
 pthB_CFBP1851
 tal18H2_CFBP6164R
 tal18H_CFBP7767R
961
 tal18H_CFBP6975
 tal18H_CFBP6546R
997
 tal18H_CFBP4885
 tal18H_CFBP6167
984
 tal18H_CFBP6166
 tal18H_CFBP6164
 tal18H_CFBP6982
 tal19E_CFBP412
 tal23A_CFBP6994R
755
 tal23A_CFBP6992
 tal23A_CFBP6989
 tal22C_CFBP6991
 tal23A_CFBP6996R
 tal23A_CFBP6166
 tal23A_CFBP6167
 tal23A_CFBP6988R
978
 tal22B_CFBP6165
 tal21D_CFBP6164
 tal23A_CFBP6990
 tal19I_CFBP6546R
 tal23A_CFBP4885
 tal23A_CFBP7767R
 tal19E_CFBP6982
 tal23A_CFBP6975
 X. translucens pv. undulosa
0.02
b
 tal18H_CFBP6167
 tal18H_CFBP6982
 tal18H_CFBP6166
 tal18H_CFBP6975
1000
 tal18H_CFBP6546R
 tal18H_CFBP4885
 tal18H2_CFBP6164
 tal18H_CFBP6164
 tal18H_CFBP7767R
 PthC1_ICMP8435
914
 PthB_B69
 PthC_C340
984
 PthC2_ICMP8435
 pthB_CFBP1851
 tal18G_CFBP6989
517
 tal18G_CFBP6991
627
 tal18G_CFBP6988
 tal18G_CFBP6990
1000
 tal20F_CFBP6991
 tal20F_CFBP6988
 tal20F_CFBP6989
 tal20F_CFBP6990
 tal21D_CFBP6164
640
 tal19E_CFBP412
808
 tal19E_CFBP6982
 tal19I_CFBP6546R
 tal23A_CFBP6994R
 tal23A_CFBP6988R
 tal23A_CFBP6167
1000
 tal23A_CFBP6989
 tal22B_CFBP6165
 tal23A_CFBP6975
 tal23A_CFBP6166
692
 tal23A_CFBP6992
 tal23A_CFBP6990
 tal23A_CFBP6996R
 tal23A_CFBP7767R
 tal23A_CFBP4885
 tal22C_CFBP6991
X. translucens pv. undulosa
0.02
Additional file 19: Figure S8. Phylogenetic trees of N-ter- and C-ter-encoding regions of tal genes used for the Kishino-Hasegawa-Templeton test. Bootstrap values greater than 50% are shown for 1000 replicates and horizontal scale bars represent the number of nucleotide substitutions per site. a. ML tree constructed on a nucleotide alignment of the N-ter-encoding region of tal genes from X. citri pv. fuscans, X. citri pv. aurantifolii, X. phaseoli pv. phaseoli and X. phaseoli pv. manihotis strains using a tal gene from X. translucens pv. undulosa XT4699 as outgroup. b. ML tree constructed on a nucleotide alignment of the C-ter-encoding region of tal genes from X. citri pv. fuscans, X. citri pv. aurantifolii, X. phaseoli pv. phaseoli and X. phaseoli pv. manihotis strains using a tal gene from X. translucens pv. undulosa XT4699 as outgroup.
